# SCML2 inhibits PrimPol-mediated DNA replication repriming and regulates replication gap-associated cancer cell vulnerability

**DOI:** 10.1126/sciadv.aee4738

**Published:** 2026-10-23

**Authors:** Xin Shi, Qianqian Peng, Jing Guo, Wei Wu, Ping Wang, Xin Zhou, Qiang Chen

**Affiliations:** ^1^ Department of Gastrointestinal Surgery, Frontier Science Center for Immunology and Metabolism, Medical Research Institute, Zhongnan Hospital of Wuhan University, Wuhan University, Wuhan 430071, China.; ^2^ State Key Laboratory of Virology, Wuhan Institute of Virology, Chinese Academy of Sciences, Wuhan, China.; ^3^ Clinical Medical Research Center of Peritoneal Cancer of Wuhan, Wuhan, China.; ^4^ Hubei Key Laboratory of Tumor Biological Behaviors, Zhongnan Hospital of Wuhan University, Wuhan, China.; ^5^ Hubei Province Cancer Clinical Study Center, Zhongnan Hospital of Wuhan University, Wuhan, China.

## Abstract

PrimPol-mediated fork repriming generates single-stranded DNA (ssDNA) gaps during DNA damage tolerance (DDT), which, if not properly repaired, can threaten genomic stability. PrimPol is a highly error-prone polymerase. Thus, PrimPol activity should be tightly regulated to fit into DDT and remain low during unstressed DNA replication. Human sex comb on midleg like-2 (SCML2), a Polycomb protein containing MBT repeats, has been identified as a component of the noncanonical PRC1 complex and a regulator of p53 and CHK1 stability. Here, we show that SCML2A is a suppressor of PrimPol. SCML2A directly binds two domains of PrimPol: Its RBR domain binds the C terminus of PrimPol, and its SAM domain binds the amino-terminal AEP domain, thereby inhibiting ssDNA-binding capacity of the AEP domain. SCML2 depletion leads to increased nascent DNA synthesis and accumulation of PrimPol-mediated ssDNA gaps under replication stress. SCML2 depletion also enhances PrimPol engagement during unstressed DNA replication, leading to subsequent accumulation of ssDNA gaps, which are filled by REV1/POLζ-mediated translesion synthesis (TLS) and RAD51-mediated template switching (TS). SCML2 depletion confers cellular hypersensitivity to RAD51 or REV1/POLζ inhibition, which is associated with increased accumulation of ssDNA gaps. Furthermore, CDK2 phosphorylates SCML2A at Ser^267^, which suppresses RBR-mediated PrimPol interaction. Cyclin E1 overexpression leads to CDK2-mediated SCML2A Ser^267^ hyperphosphorylation, which releases the inhibitory effect of SCML2A on PrimPol and subsequently induces ssDNA gap accumulation. Collectively, we identify SCML2A as a negative regulator of PrimPol.

## INTRODUCTION

Accurate and timely DNA replication is crucial for maintaining genome stability. However, this process is constantly threatened by obstacles of both endogenous and exogenous origin, which can impede the progression of the replication machinery and induce DNA replication stress. The slowing or stalling of replication fork progression, commonly observed upon DNA replication stress, is a major source of genome instability in cancer (*1*, *2*). In response to DNA replication stress, evolutionarily conserved DNA damage tolerance (DDT) pathways have evolved to facilitate the bypass of DNA lesions and sustain DNA synthesis. For instance, stalled replication forks can undergo fork reversal, generating four-way junctions that stabilize stalled forks and promote their recovery (*3*). In addition to fork reversal, replication forks can bypass DNA lesions via PrimPol-mediated repriming (*4*–*6*). Specifically, PrimPol catalyzes de novo primer synthesis following various DNA lesions, including canonical base damage and interstrand crosslinks, thereby restarting DNA synthesis downstream of lesion sites (*7*–*9*). Notably, PrimPol-mediated repriming generates single-stranded DNA (ssDNA) gaps behind the replication forks. Recent studies have shown that these ssDNA gaps can be filled through postreplicative translesion synthesis (TLS) mediated by REV1/POLζ, as well as through RAD51-mediated template switching (TS) followed by homologous recombination (*9*–*11*).

The human *PrimPol* gene belongs to the archaea-eukaryotic primases (AEP) superfamily. It was first predicted by the in silico classification of AEP superfamily members and localizes to both mitochondria and the nucleus of human cells (*12*). Although PrimPol functions as both a DNA primase and a TLS DNA polymerase (*4*, *6*, *13*, *14*), its primase activity—rather than its TLS activity—constitutes its major role in mitigating DNA replication stress in vivo (*6*, *15*–*17*). Specifically, the primase activity enables PrimPol to synthesize a primer downstream of a DNA lesion, thereby reinitiating DNA replication. These DNA primers are subsequently extended by the replicative polymerase POLε shortly after repriming (*13*). PrimPol comprises an N-terminal AEP domain, a C-terminal replication protein A (RPA)–binding domain (RBD), and a zinc finger domain (*17*, *18*). The catalytic AEP core of PrimPol contains two conserved modules: ModN and ModC. ModN directly interacts with the ssDNA template, whereas ModC engages incoming nucleotides (*18*). The RBD domain of PrimPol interacts with the ssDNA-binding protein RPA, which recruits PrimPol to chromatin (*19*).

Like other TLS polymerases, PrimPol is highly error prone (*20*, *21*). A crucial question, therefore, is which factors determine when PrimPol-mediated repriming should be restrained or stimulated to prevent fork stalling or collapse. PrimPol is regulated at multiple levels. ATR positively regulates PrimPol mRNA expression (*15*). In addition, USP36 and WRNIP1 modulate PrimPol stability (*22*, *23*). Both CHK1 and PLK1 phosphorylate PrimPol at distinct sites to regulate its activity (*24*, *25*). Notably, several factors—including the BRCA1-MCM10 complex (*26*), the CST (CTC1-STN1-TEN1) complex (*27*), and the POLι-p53 complex (*28*)—modulate PrimPol activity by inhibiting its recruitment to stressed replication forks. Furthermore, fork reversal and PrimPol-mediated repriming appear to compete with each other at stressed replication forks, with fork reversal being primarily engaged (*29*). Several factors regulate PrimPol activity by balancing fork reversal and repriming (*30*–*32*). However, several questions remain unanswered regarding the role of PrimPol in DNA replication. For example, which factors restrain PrimPol-mediated repriming to promote DNA replication fidelity in cells not exposed to exogenous stress? In this study, we identified SCML2A as a direct binding partner of PrimPol and elucidated the mechanism whereby SCML2A inhibits PrimPol activity.

Human sex comb on midleg-like 2 (SCML2) is a component of the noncanonical Polycomb repressive complex 1 (PRC1) (*33*). The human *SCML2* gene encodes two isoforms, SCML2A and SCML2B, which perform distinct biological functions. SCML2A binds PRC1 via its sterile α motif (SAM) domain and directly interacts with RNA via its RNA binding region (RBR) (*33*, *34*). SCML2B stabilize p21 and regulate CDK2/cyclin E1 activation (*35*). In mice, SCML2 is exclusively expressed in the testes and is essential for male germline development (*36*, *37*). It regulates mouse spermatogenesis by modulating epigenetic programming (*36*, *38*). In addition, high expression of SCML2 has been found in multiple tumor types (*39*, *40*). In our previous work, we characterized the relationship between SCML2 and the DNA damage response (*41*). We showed that DNA damage stabilizes SCML2 via CHK1-mediated phosphorylation at Ser^570^, which enhances cellular resistance to DNA-damaging agents. We identified SCML2 as a regulator of chemotherapy resistance and uncovered a positive feedback loop between SCML2 and CHK1 after DNA damage, which promotes chemoresistance to DNA-damaging agents.

In this study, we established SCML2A as a suppressor of PrimPol through direct binding. SCML2A inhibited PrimPol activity via preventing its ssDNA binding capacity. SCML2 deficiency resulted in PrimPol hyperactivity, even during unstressed DNA replication. SCML2 depletion exhibited synthetic lethality with genotoxic agents, such as cisplatin, RAD51 or REV1 inhibitors. Thus, we identified SCML2A as a potential therapeutic target, as SCML2A depletion leads to ssDNA gap–associated therapeutic vulnerability.

## RESULTS

### SCML2 depletion accelerates nascent DNA synthesis upon DNA replication stress

To determine whether SCML2 plays a role in DNA replication stress, we assessed the speed of ongoing DNA replication in the presence or absence of hydroxyurea (HU) using DNA fiber analysis. HCT116 cells were pulse-labeled with 5-chloro-2′-deoxyuridine (CldU) for 20 min, followed by 5-iodo-2′-deoxyuridine (IdU) labeling for 60 min with or without 50 μM HU (Fig. 1A). IdU tract length was measured to quantify replication fork progression under replication stress by DNA fiber stretching on glass slides. HU treatment markedly reduced IdU tract length, indicating replication fork stalling. Notably, SCML2-depleted cells exhibited longer IdU tracts following HU treatment compared to control cells (Fig. 1A). This effect was also observed in SCML2 knockout (KO) cells and was further confirmed using two independent SCML2-targeting small interfering RNAs (siRNAs). Moreover, SCML2 depletion did not affect normal DNA replication progression without HU treatment (fig. S1A). We also calculated the IdU/CldU ratio to normalize for experimental variations. Consistently, SCML2 depletion increased the IdU/CldU ratio after HU treatment (fig. S1B). Furthermore, we analyzed the effect of SCML2 depletion on IdU tract length after short-time HU treatment. The SCML2 depletion–induced increase of IdU length was observed following only 10 min of HU treatment (Fig. 1B). Thus, these results suggest that SCML2 deficiency enhances replication fork recovery or restart after HU-induced stalling.

**Fig. 1.**
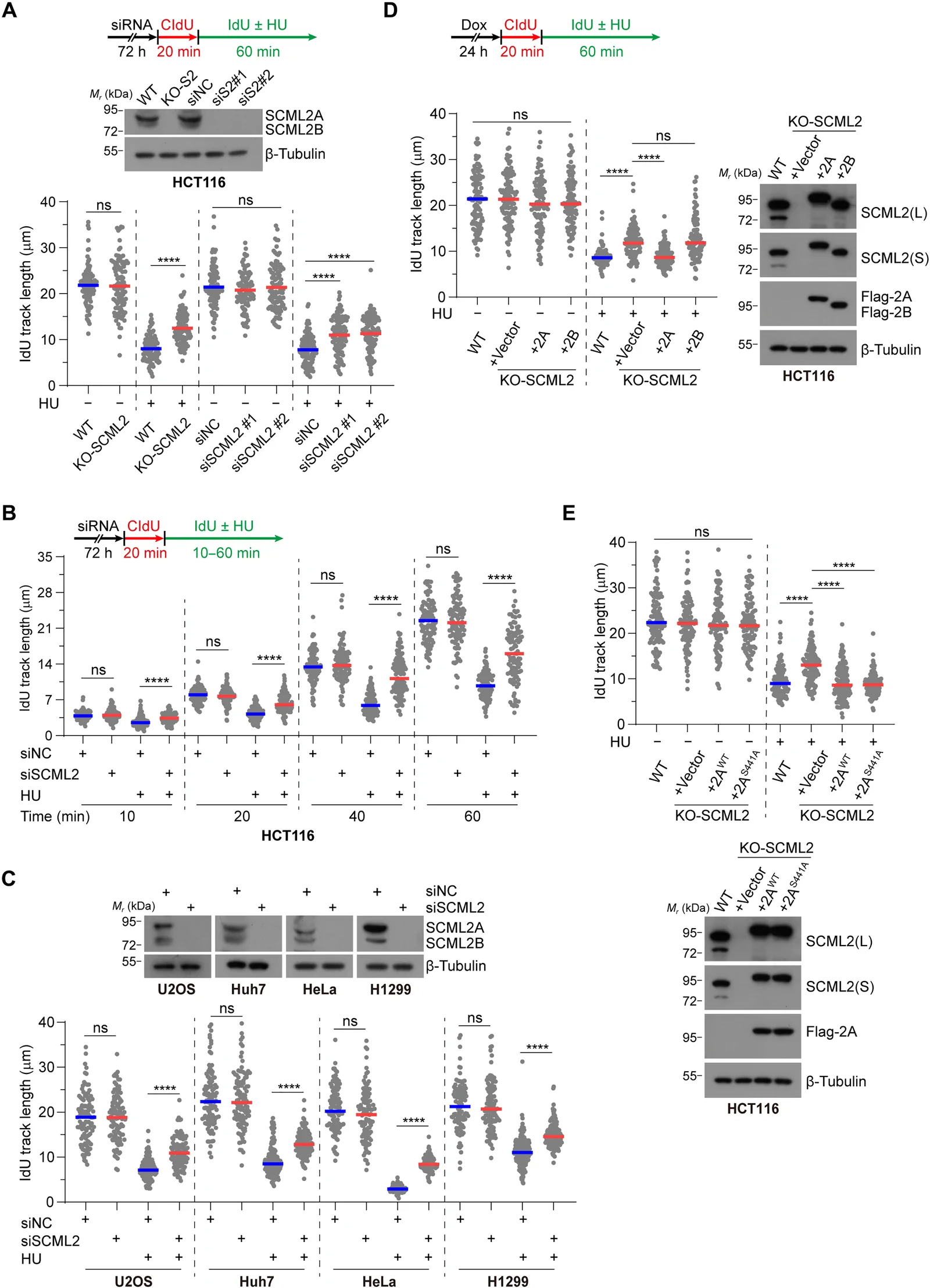
SCML2 depletion accelerates nascent DNA synthesis upon DNA replication stress. (**A**) Schematic of DNA fiber tract analysis. HCT116 cells were transfected with control siRNA or SCML2 siRNA before CldU labeling. Red tracts, CldU; green tracts, IdU, with or without 50 μM HU (top). Levels of SCML2 were analyzed by Western blot (middle). Dot plot and median of ldU track lengths in HCT116 cells subjected to the indicated experimental conditions (bottom). *n* = 3. (**B**) Schematic of DNA fiber tract analysis (top). Dot plot and median of ldU track lengths in HCT116 cells transfected with siNC or siSCML2, with or without treatment of 50 μM HU for 0 to 60 min (bottom). *n* = 3. (**C**) Levels of SCML2 were analyzed by Western blot (top). Dot plot and median of ldU track lengths in U2OS, Huh7, HeLa, and H1299 cells with SCML2 deficiency with or without 50 μM HU for 60 min (bottom). *n* = 3. (**D**) Schematic of DNA fiber tract analysis (top). Dot plot and median of ldU track lengths in HCT116 wild-type (WT) cells and SCML2 KO cells overexpressing SCML2A or SCML2B treated with or without 50 μM HU for 60 min (left). Levels of SCML2A and SCML2B were analyzed by Western blot. S, short exposure; L, long exposure (right). *n* = 3. (**E**) Dot plot and median of ldU track lengths in HCT116 WT cells and SCML2 KO cells overexpressing SCML2A^WT^ or SCML2A^S441A^ treated with or without 50 μM HU for 60 min (top). Levels of SCML2A^WT^ and SCML2A^S441A^ were analyzed by Western blot (bottom). *n* = 3. A minimum of 100 DNA fibers was analyzed for each condition (*n* = 3). Bar: median. *P* values were calculated by the Mann-Whitney test (****P < 0.0001). ns, nonsignificant.

Previously, we and others reported that SCML2 regulates p53 and p21 stability via its association with USP7 (*33*, *35*, *41*). Given that p53, p21, and USP7 are known to regulate DNA replication or participate in DNA replication stress tolerance (*42*–*46*), we investigated whether these proteins contribute to the enhanced replication fork recovery induced by SCML2 depletion. We first analyzed IdU tract length following HU treatment in multiple cell lines, including U2OS [p53 wild type (WT)], Huh7 (p53 Y220C mutant), HeLa (p53 mutant), and H1299 (p53 null) cells. SCML2 depletion increased IdU tract length after HU treatment in all four cell lines (Fig. 1C), indicating that SCML2’s role in replication stress is independent of p53. We next repeated similar experiments in HCT116 p53-KO cells. Consistent with previous studies, the normal DNA replication speed decreased in p53-KO cells (fig. S1C) (*42*, *44*, *46*). SCML2 depletion increased IdU tract length after HU treatment in p53-KO cells (fig. S1D), confirming that p53 is dispensable for SCML2 depletion induced DNA replication fork recovery after HU treatment.

We next tested the effect of SCML2 depletion in p21-KO cells. Although p21 deficiency exerted opposing effects on DNA replication speed among three selected clones due to variable extents of p21 depletion (fig. S1E) (*45*), SCML2 depletion consistently increased IdU tract length after HU treatment in all clones (fig. S1F). In addition, we reproduced the findings of a previous study using the same siRNAs against p21 (*45*). Consistently, we found that distinct siRNAs, or the same siRNA at varying concentrations, reduced p21 to different degrees (fig. S1I) and exerted opposing effects on DNA replication speed (fig. S1, G and H, top). Notably, under both conditions, SCML2 depletion induced an increase in IdU tract length after HU treatment (fig. S1, G and H, bottom). Last, since SCML2B (but not SCML2A) binds to and regulates p21 (*35*, *41*), we reintroduced either isoform into HCT116 SCML2-KO cells. Expression of SCML2A, but not SCML2B, diminished the SCML2 deficiency–induced increase of IdU tract length after HU treatment (Fig. 1D). Collectively, these results demonstrate that the effect of SCML2 on replication fork recovery is p21 independent.

Our previous work showed that SCML2 Ser^441^ is the key residue mediating USP7 association and that the S441A mutant lacks USP7-binding capacity (*41*). Overexpression of SCML2A S441A in SCML2-KO cells exerted the same effect on the IdU tract length as WT SCML2A (Fig. 1E). Thus, SCML2-mediated regulation of replication restart following HU treatment is independent of its association with USP7.

### SCML2 depletion promotes PrimPol-mediated increase of DNA synthesis

One mechanism of fork recovery under replication stress involves the repriming polymerase PrimPol (*29*, *47*). We therefore tested whether PrimPol is required for the increased fork speed after HU treatment observed in SCML2-depleted cells. PrimPol depletion abolished the increase in replication fork recovery caused by SCML2 depletion [Fig. 2A and figs. S1F (lanes 5 to 8) and S2A]. Since PrimPol is also involved in the DNA replication stress induced by the DNA polymerase α inhibitor CD437 under CLASPIN overexpression (*24*), we further examined SCML2’s role in CD437-induced DNA replication stress. SCML2 depletion increased IdU tract length in CD437-treated HeLa cells, and this effect was again reversed by PrimPol knockdown (Fig. 2B). The effect of SCML2 depletion on IdU tract length after CD437 treatment was the same as CLASPIN overexpression (fig. S2, B and C), although SCML2 depletion did not affect CHK1 activation after 30-min CD437 treatment (fig. S2D). Our previous study revealed that SCML2 depletion reduced CHK1 stability after long-time replication stress, for example, that induced by 12-hour treatment with a high dose of HU (3 mM) (fig. S2E) (*41*). However, the effect of SCML2 depletion on DNA replication fork recovery was evident within 1 hour of exposure to replication stress (either low dose HU or CD437) with no detectable change in CHK1 stability or activation at this time point (fig. S2, D and F). Collectively, these results indicate that the enhanced DNA replication recovery caused by SCML2 depletion under replication stress depends on PrimPol and that this effect is unrelated to its function in CHK1 stability. Notably, while SCML2 depletion and CLASPIN overexpression produced identical effects on PrimPol activation after CD437 treatment, the potential link between SCML2 depletion and CHK1-mediated PrimPol S255 phosphorylation has yet to be established and will require additional studies to resolve.

**Fig. 2.**
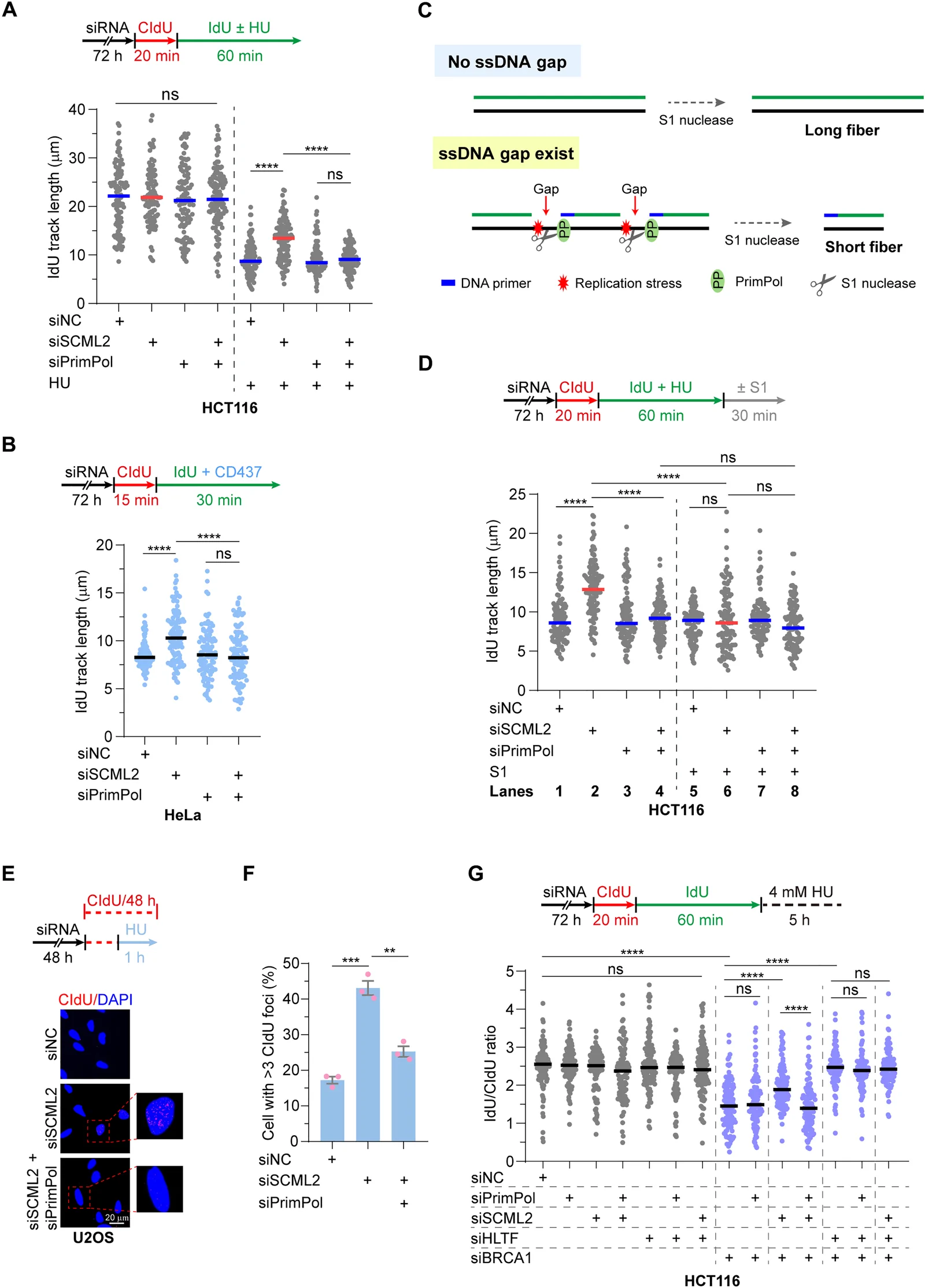
SCML2 depletion promotes PrimPol-mediated increase of DNA synthesis after DNA replication stress. (**A**) Schematic of DNA fiber tract analysis (top). Dot plot and median of ldU track lengths in HCT116 cells exposed to 50 μM HU for 60 min after treatment with the indicated siRNAs (bottom). *n* = 3. (**B**) Dot plot and median of ldU track lengths in HeLa cells exposed to 5 μM CD437 for 30 min after treatment with siRNA targeting SCML2. *n* = 3. (**C**) Schematic for detection of ssDNA gaps by the ssDNA-specific S1 nuclease. (**D**) Schematic of the IdU/CldU pulse-labeling protocol with 50 μM HU treatment followed by S1 nuclease (20 U/ml) digestion (top). Dot plot and median of ldU tract lengths (in micrometers) in HCT116 cells subjected to the indicated experimental conditions (bottom). *n* = 3. (**E**) Schematic of nondenaturing CldU staining analysis (top). Representative images of CldU staining (red) and 4′,6-diamidino-2-phenylindole (DAPI) staining (blue) in U2OS cells exposed to 50 μM HU after treatment with the indicated siRNAs. Scale bar, 20 μm (bottom). (**F**) Percentage of U2OS cells containing >3 CldU foci per nucleus, as shown in (E). Values from individual experiments are indicated as dots. Columns represent the mean ± SEMs of independent biological replicates (*n* = 3). Statistical analysis was conducted by an unpaired *t* test (**P < 0.01 and ***P < 0.001). (**G**) Schematic of DNA fiber tract analysis with 4 mM HU for 5 hours (top). Dot plot and median of ldU/CldU ratio in HCT116 cells subjected to the indicated experimental conditions (bottom). *n* = 3. A minimum of 100 DNA fibers was analyzed for each condition (*n* = 3). Bar: median. *P* values were calculated by the Mann-Whitney test (****P < 0.0001).

PrimPol-mediated de novo priming facilitates fork progression by allowing the replisome to skip over barriers, leaving an ssDNA gap behind the fork (*4*, *13*, *14*). To detect such gaps, we treated cells with 50 μM HU and then incubated permeabilized cells with or without the ssDNA-specific S1 nuclease, which cleaves replication intermediates containing ssDNA gaps or DNA secondary structures (Fig. 2C) (*48*, *49*). S1 nuclease treatment specifically shortened IdU tracts in SCML2-depleted cells under HU-induced replication stress (Fig. 2D, lane 6 versus lane 2), suggesting that replication acceleration in these cells occurs discontinuously and is accompanied by increased ssDNA gaps. To test whether PrimPol mediates this effect, we knocked down PrimPol and assessed fork progression. In HU-treated SCML2-depleted cells, PrimPol knockdown shortened IdU tracts (Fig. 2D, lane 4 versus lane 2), but S1 nuclease treatment did not further reduce tract length (Fig. 2D, lane 8 versus lane 4), demonstrating that SCML2 depletion–induced replication acceleration after replication stress was PrimPol dependent and led to ssDNA gap accumulation. Next, to directly assess ssDNA accumulation, we performed nondenaturing immunostaining for CldU in U2OS cells. SCML2 depletion resulted in a more than twofold increase in CldU foci–positive cells after HU treatment, and this increase was reduced by PrimPol knockdown (Fig. 2, E and F). Furthermore, SCML2 depletion led to PrimPol-dependent micronucleus formation upon HU-induced DNA replication stress (fig. S2, G and H). Last, as cisplatin treatment can also induce PrimPol engagement, we also tested the effect of SCML2 depletion on DNA replication after cisplatin treatment and found that SCML2 depletion induced PrimPol-dependent ssDNA gap accumulation in cisplatin-treated cells (fig. S2I). Together, our results demonstrate that PrimPol drives accelerated replication fork progression after replication stress in SCML2-depleted cells, thereby inducing induces ssDNA gap accumulation.

Several reports have indicated that PrimPol expression levels are positively correlated with its engagement under replication stress. We examined PrimPol mRNA and protein levels and found that neither SCML2 depletion nor SCML2A overexpression affected PrimPol expression (fig. S2, J and K). Another mechanism for increased PrimPol activity after replication stress is the suppression of fork reversal (*9*, *24*, *26*, *32*). We compared the effect of SCML2 depletion on ssDNA gap accumulation after HU treatment with that of fork reversal suppressing. The effect of SCML2 depletion on replication fork recovery and ssDNA accumulation was the same as depletion of HLTF or SMARCAL1, key factors promoting fork reversal (fig. S2, L and M). Codepletion of SCML2 with HLTF or SMARCAL1 exhibited the same effect on ssDNA accumulation as SCML2 depletion alone. To further investigate the relationship between SCML2 and fork reversal, we tested nascent DNA stability after 4 mM HU treatment in BRCA1-depleted cells. BRCA deficiency leads to degradation of nascent DNA, which can be rescued by fork reversal suppression (*50*). Consistent with this report, HLTF depletion efficiently rescued nascent DNA degradation caused by BRCA1 depletion (Fig. 2G). In contrast, SCML2 depletion exhibited only a weak effect on preventing nascent DNA degradation compared to HLTF depletion. The rescue of nascent DNA degradation induced by SCML2 depletion, but not by HLTF depletion, was dependent on PrimPol (Fig. 2G and fig. S2N). We speculate that SCML2 depletion partially rescues nascent DNA degradation in BRCA-deficient conditions through promoting PrimPol engagement, as has been proposed in a previous study (*15*). Notably, codepletion of SCML2 and HLTF conferred protection against BRCA1 loss–induced nascent DNA degradation comparable to that observed with HLTF depletion alone. Thus, we conclude that the role of SCML2 in regulating PrimPol may be not related to the DNA replication fork reversal process.

### SCML2 interacts with PrimPol through RBR domain

To study how SCML2 regulates PrimPol activity, we first examined whether SCML2 interacts with PrimPol. Coimmunoprecipitation (Co-IP) assays in human embryonic kidney (HEK) 293T cells showed that both SCML2A and SCML2B bound to PrimPol (Fig. 3A). To map the interaction domain, we generated truncated SCML2 mutants (Fig. 3B). Notably, only mutants containing the RBR region retained PrimPol binding, whereas the ΔRBR mutant did not (Fig. 3C), indicating that SCML2 interacts with PrimPol via its RBR domain.

**Fig. 3.**
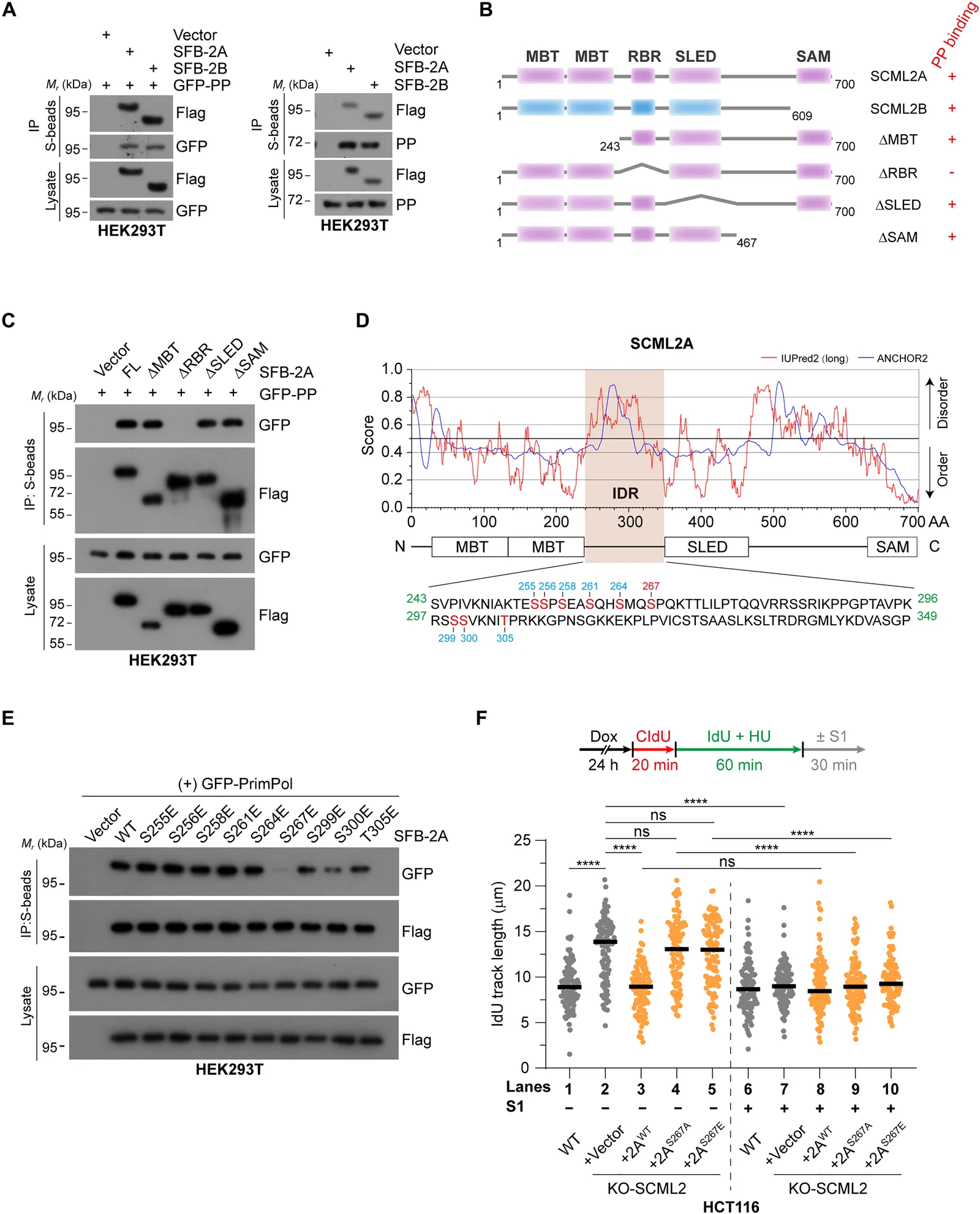
SCML2 inhibits PrimPol through RBR domain–mediated interaction. (**A**) The interaction of SFB-SCML2A (SFB-2A), SFB-SCML2B (SFB-2B) with exogenous green fluorescent protein–PrimPol (GFP-PP), or endogenous PrimPol (PP) was detected by Co-IP in HEK293T cells. 2A is the abbreviation of SCML2A, 2B is the abbreviation of SCML2B, and PP is the abbreviation of PrimPol in all figures, unless otherwise specified. *n* = 4. (**B**) A schematic representation of SCML2 truncations. (**C**) The interaction of GFP-PrimPol with SFB-SCML2A truncations was detected by Co-IP in HEK293T cells. Results indicated that the RBR domain was responsible for mediating this binding. *n* = 4. (**D**) Diagram of predicted SCML2A IDRs. The orange area represents the RBR region, which is also the IDR region. The red and blue amino acid represents the nine reported phosphorylation sites in the RBR region (bottom). (**E**) The interaction of GFP-PrimPol with SFB-SCML2A mutation as shown was detected by Co-IP in HEK293T cells. *n* = 4. (**F**) Schematic of DNA fiber tract analysis with S1 nuclease digestion (top). Dot plot and median of ldU tract lengths (in micrometers) in HCT116 WT cells and KO-SCML2 cells overexpressing SCML2A^WT^, SCML2A^S267A^, or SCML2A^S267E^ subjected to the indicated experimental conditions (bottom). *n* = 3. ****P < 0.0001.

Bioinformatic analysis using IUPred2A predicted that the RBR region is highly disordered (Fig. 3D) (*51*), a finding supported by AlphaFold3 predictions (fig. S3A). Intrinsically disordered regions (IDRs) often facilitate protein-protein interactions in signal transduction and can be regulated by posttranslational modifications such as phosphorylation (*52*, *53*). Since the RBR region contains nine reported phosphorylation sites (Fig. 3D) (*54*, *55*), we investigated whether phosphorylation modulates SCML2-PrimPol binding. Mutating these sites to glutamic acid (E) to mimic phosphorylation revealed that only the S267E mutant reduced SCML2-PrimPol binding (Fig. 3E). The S267A mutant also blocked this binding (fig. S3B), indicating that SCML2 Ser^267^ is essential for the SCML2-PrimPol interaction.

We further examined the functional impact of SCML2 S267 mutations on PrimPol-mediated repriming under replication stress. SCML2A WT, but not the S267E or S267A mutant, inhibited PrimPol-mediated repriming and ssDNA gap accumulation (Fig. 3F and fig. S3, C and D). These results demonstrate that SCML2A inhibits PrimPol-mediated repriming through an RBR domain–dependent interaction with PrimPol.

### SAM domain of SCML2A is essential for its inhibition on PrimPol

We have shown that SCML2A inhibits PrimPol through RBR-mediated binding. Two questions remain to be addressed: (i) Why SCML2A, but not SCML2B, suppresses PrimPol activity; and (ii) how SCML2A inhibits PrimPol activity. To elucidate the molecular mechanism by which SCML2A inhibits PrimPol, we generated truncated PrimPol mutants (Fig. 4A) to map the domains mediating its interaction with SCML2. To determine whether SCML2 directly associates with PrimPol, we performed Biacore assays using purified proteins (fig. S4A). Both SCML2A and SCML2B bound directly to PrimPol (Fig. 4B and fig. S4B). ΔAEP (372-end) of PrimPol tightly interacted with both SCML2A and SCML2B. In contrast, neither the ZF (372-510) nor the RBD domain (488-end) alone interacted with SCML2, indicating that both domains are required for this interaction. Unexpectedly, 1 to 354 amino acids (AEP) and 1 to 487 amino acids of PrimPol only interacted with SCML2A, but not with SCML2B (Fig. 4B), revealing a distinct binding mode between the PrimPol AEP domain and SCML2A. These observations were consistent with Co-IP results in HEK293T cells (Fig. 4C), confirming that PrimPol directly binds to SCML2A through multiple domains and that only SCML2A contains the capacity to bind the AEP domain of PrimPol.

**Fig. 4.**
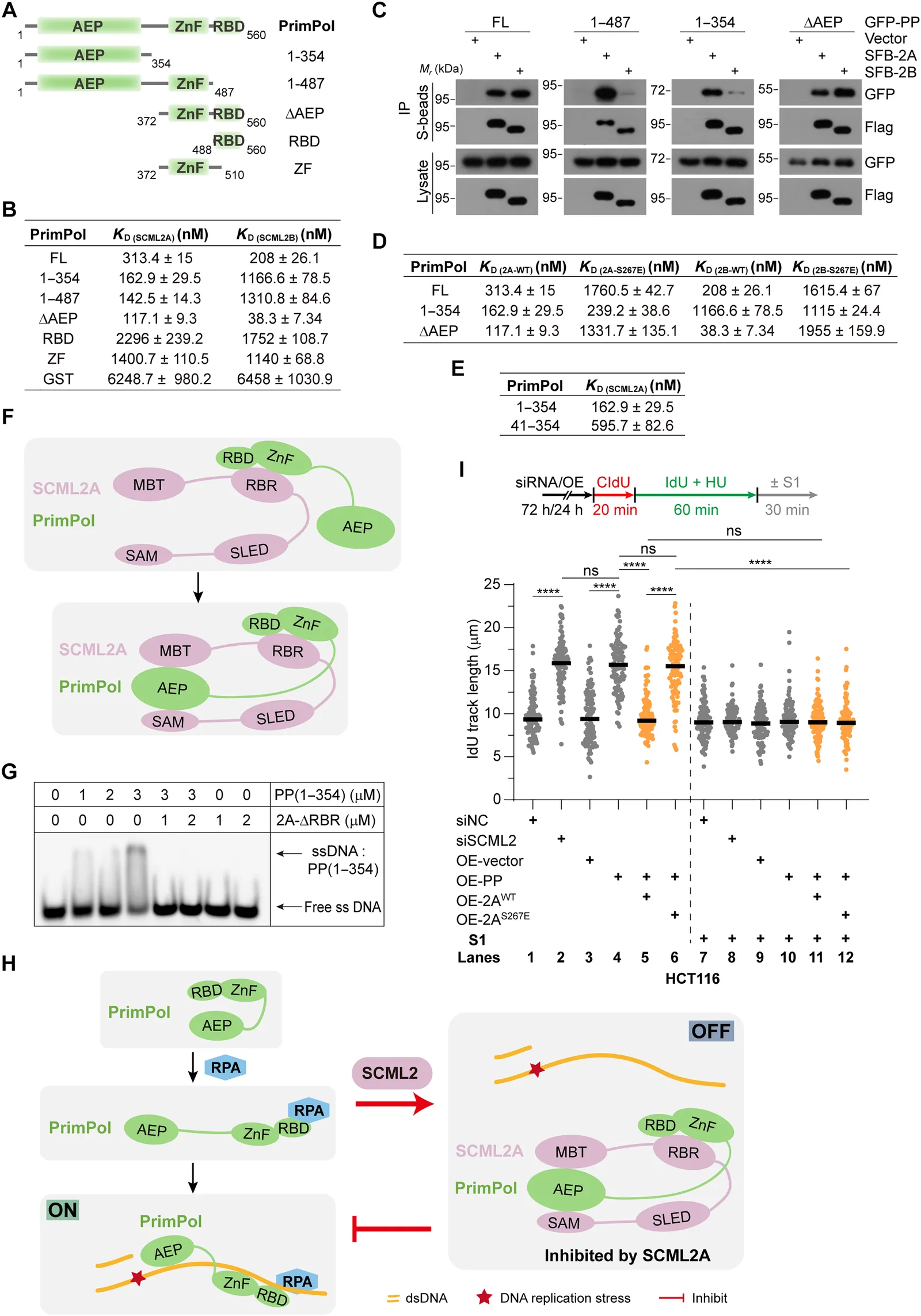
SAM domain of SCML2A is essential for its inhibition on PrimPol. (**A**) A schematic representation of PrimPol truncations. (**B**) Biacore analysis of dissociation constant (*K*_D_) between SCML2A or SCML2B and PrimPol truncations. The *K*_D_ value was calculated using Biacore Evaluation Software Version 3.2.1 (GE Healthcare). Data are presented as mean ± SD from three independent experiments. (**C**) Co-IP of SCML2A or SCML2B with PrimPol truncations in HEK293T cells. *n* = 4. (**D**) Biacore analysis of dissociation constant (*K*_D_) between SCML2A^WT^, SCML2A^S267E^, SCML2B^WT^, or SCML2B^S267E^ and PrimPol truncations. (**E**) Biacore analysis of SCML2A binding to PrimPol (1 to 354) or (41 to 354). (**F**) Schematic diagram of the combination of SCML2A and PrimPol. Pink, SCML2A; green, PrimPol. (**G**) EMSA results showed that SCML2A^ΔRBR^ inhibited the binding of PrimPol (1 to 354) and ssDNA. *n* = 5. (**H**) Schematic diagram of SCML2A inhibiting the function of PrimPol. (**I**) Schematic of DNA fiber tract analysis (top). Dot plot and median of ldU tract lengths (in micrometers) in HCT116 cells subjected to the indicated experimental conditions (bottom). *n* = 3. ****P < 0.0001.

Next, we determined which domain of PrimPol was responsible for SCML2 RBR domain–mediated binding. Glutathione *S*-transferase (GST) pull-down assays demonstrated that SCML2B WT tightly interacts with PrimPol, but not SCML2B S267E (fig. S4C). Biacore assays showed that the S267E mutant reduced interaction with the PrimPol ΔAEP (372-end) but not with the 1 to 354 amino acids (Fig. 4D and fig. S4D), suggesting that the RBR domain of SCML2 binds to the C-terminal region (ΔAEP, 372-end) of PrimPol. We predicted the structures of SCML2 and PrimPol using AlphaFold3 (fig. S4E). The RBR domain contains abundant positively charged amino acids, and the surface of the C-terminal region of PrimPol is strongly negatively charged, indicating that electrostatic affinity between these two domains may drive this interaction.

Furthermore, we focused on the difference between SCML2A and SCML2B in the interaction with PrimPol. SCML2A, but not SCML2B, interacted with 1 to 354 amino acids and 1 to 487 amino acids of PrimPol (Fig. 4, B and C), indicating that SCML2A can bind the AEP domain (1 to 354 amino acids) of PrimPol. The key structural difference between SCML2A and SCML2B is the C-terminal SAM domain (608 to 700 amino acids) in SCML2A (Fig. 3B). We hypothesized that the SAM domain might mediate its binding to the AEP domain of PrimPol. However, Co-IP assays failed to detect this interaction (fig. S4F). We used AlphaFold3 to predict the association model between SCML2A and the AEP (1 to 354 amino acids) domain. One predicted model showed that both the SAM and MBT domains of SCML2A contribute to binding the AEP domain, with the N-terminal α helix (1 to 40 amino acids) of the AEP domain clamped by these two domains (fig. S4G). To validate this model, we compared the binding capacity of the 1 to 354 amino acids and 41 to 354 amino acids to SCML2A using Biacore assays. Consistent with our prediction, 41 to 354 amino acids lost binding to SCML2A (Fig. 4E and fig. S4H). Thus, we revealed the binding mode between SCML2A and PrimPol (Fig. 4F). The RBR domain of both SCML2A and SCML2B is essential for their interaction with the C-terminal of PrimPol. However, the existence of SAM domain in SCML2A endows it with specific capacity to interact with the AEP domain.

We previously showed that SCML2A (not SCML2B) inhibits PrimPol-mediated repriming (Fig. 1D). Given that only SCML2A binds the AEP domain (1 to 354 amino acids), this interaction likely mediates the specific inhibitory function of SCML2A. The AEP domain of PrimPol is responsible for its enzymatic activity, particularly for ssDNA binding, which is an essential step in PrimPol-mediated repriming (*56*, *57*). Thus, we examined whether SCML2A could influence the ssDNA-binding capacity of the AEP domain. Since the RBR domain itself binds ssDNA (fig. S4I), we used the SCML2A ΔRBR mutant in the following experiments. This mutant disrupted ssDNA binding of the AEP domain (Fig. 4G), indicating that SCML2A can efficiently disrupt the ssDNA-binding capacity of the AEP domain. Although SCML2B interacts with the C-terminal of PrimPol, SCML2B cannot bind the AEP domain and lacks the inhibitory effect on PrimPol activity. Consistent with our finding that SCML2A prevents the ssDNA binding of PrimPol, SCML2A overexpression blocked the increased chromatin binding of PrimPol induced by HU treatment (fig. S4J). Collectively, we speculated that SCML2A inhibits PrimPol activity by blocking the AEP domain’s ssDNA recognition capacity via its SAM domain and that both the RBR and SAM domains are essential for SCML2A-mediated suppression of PrimPol (Fig. 4H).

It has been reported that PrimPol overexpression leads to increased PrimPol activity (*10*, *58*). We hypothesize that the balance between SCML2A and PrimPol may determine PrimPol engagement during DNA replication and that PrimPol overexpression may overcome the inhibitory effect of endogenous SCML2A on PrimPol. This hypothesis was confirmed by the following observations. SCML2 depletion exhibited the same effect as PrimPol overexpression on fork recovery upon replication stress. Overexpression of SCML2A WT, but not the S267E mutant, reversed the ssDNA gap accumulation caused by PrimPol overexpression (Fig. 4I and fig. S4K). Thus, the balance between PrimPol and SCML2A levels may determine PrimPol activity during DNA replication.

### SCML2 depletion promotes PrimPol engagement in unstressed cells

Given that SCML2A inhibits PrimPol activity via direct interaction, we hypothesized that SCML2 depletion may increase PrimPol engagement during DNA replication in the absence of exogenous stresses. To test this hypothesis, we measured DNA fiber lengths following SCML2 depletion in unstressed cells. Although SCML2 depletion did not alter global DNA replication speed, DNA fiber length in SCML2-depleted cells decreased following S1 nuclease treatment, and this decrease was rescued by PrimPol depletion (Fig. 5A, lanes 5 to 8). This result implied that SCML2 depletion may induce PrimPol-mediated formation of ssDNA gaps under unstressed conditions. Recent studies have shown that the REV1/POLζ complex and RAD51-mediated TS can repair ssDNA gaps generated by PrimPol (*10*, *11*). We confirmed that the filling of ssDNA gaps caused by SCML2 depletion after replication stress was delayed when cells were treated with the REV1 inhibitor JH-RE-06 (*59*) or the RAD51 inhibitor (B02) (fig. S5A) (*9*). Although JH-RE-06 alone did not affect global DNA replication speed or induce ssDNA gap formation in unstressed siNC cells, JH-RE-06 treatment revealed a pronounced accumulation of ssDNA gaps in SCML2-depleted cells as evidenced by a decrease in fiber length after S1 nuclease treatment (Fig. 5A, lanes 9 to 14). This effect was abolished by codepletion of PrimPol (Fig. 5A). The same results were observed in nontransformed hTERT-RPE1 cells after JH-RE-06 treatment (fig. S5B). Similarly, the RAD51 inhibitor B02 also induced PrimPol-dependent ssDNA accumulation in unstressed SCML2-depleted cells (Fig. 5A, lanes 15 to 18) (*9*). A similar phenomenon was also observed when RAD51 was codepleted with SCML2 in unstressed cells (fig. S5C), indicating that RAD51-mediated TS also contributes to ssDNA gap filling in SCML2-depleted cells. We further visualized nuclear ssDNA gaps using nondenaturing CldU immunostaining. The number of native CldU foci was increased in unstressed siSCML2 cells relative to siNC control cells, and this increase was reversed by codepletion of PrimPol. Moreover, JH-RE-06 treatment enhanced the SCML2 depletion–induced increase in ssDNA, and this enhancement was also rescued by PrimPol depletion (Fig. 5B). Thus, SCML2 depletion leads to unrestrained PrimPol engagement in the absence of exogenous stresses.

**Fig. 5.**
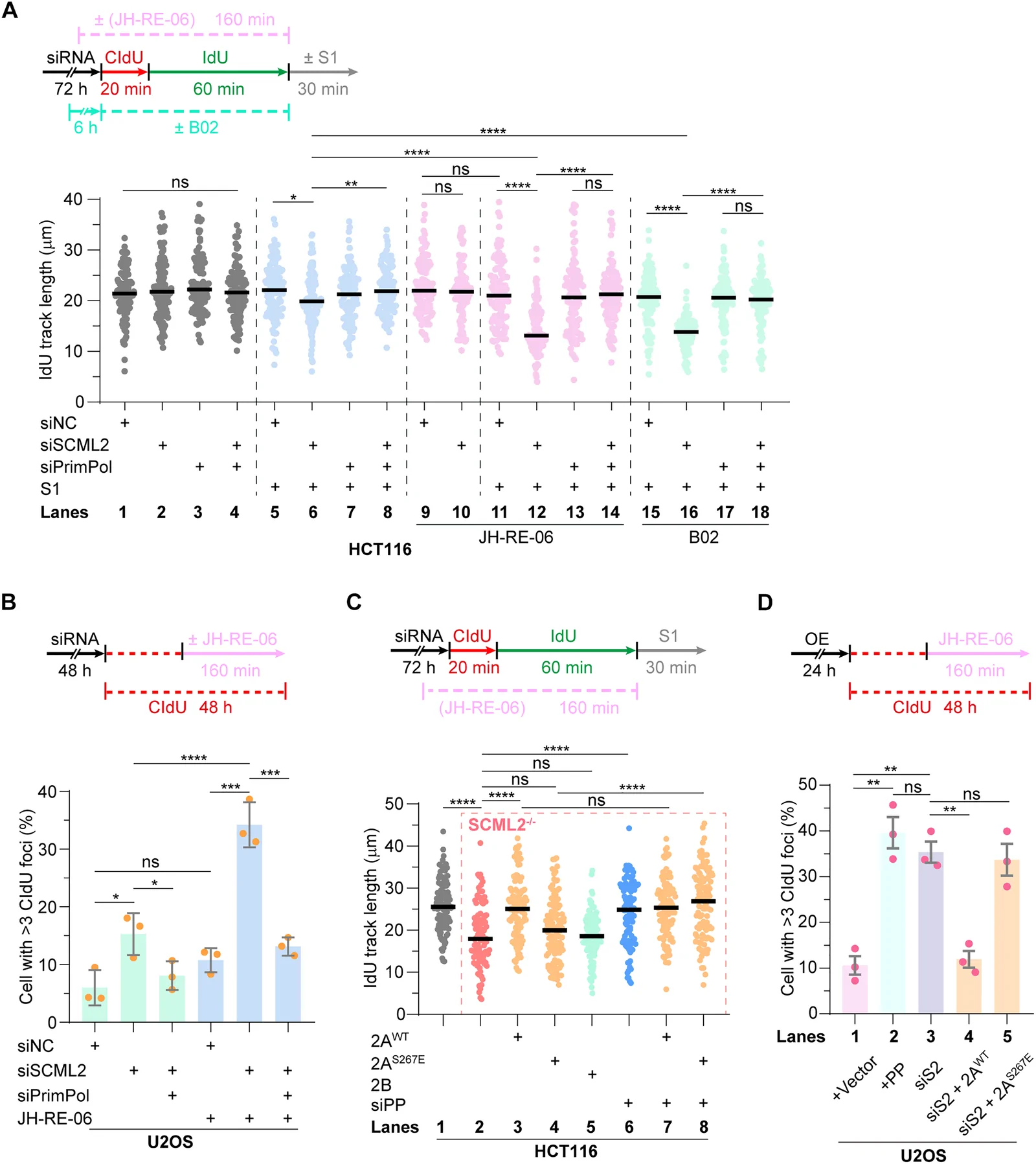
SCML2 depletion promotes PrimPol engagement in unstressed cells. (**A**) Schematic of DNA fiber tract analysis with 2.5 μM JH-RE-06 or 25 μM B02 treatment followed by S1 nuclease digestion (top). Dot plot and median of ldU tract lengths (in micrometers) in HCT116 cells subjected to the indicated experimental conditions (bottom). *n* = 3. (**B**) Schematic of nondenaturing CldU staining analysis with or without 2.5 μM JH-RE-06 (top). Percentage of U2OS cells containing >3 CldU foci per nucleus after treatment with the indicated siRNAs (bottom). Values from individual experiments are indicated as dots. Columns represent the mean ± SEMs of independent biological replicates (*n* = 3). Statistical analysis was conducted by an unpaired *t* test. (**C**) Schematic of DNA fiber tract analysis with 2.5 μM JH-RE-06 followed by S1 nuclease digestion (top). Dot plot and median of ldU tract lengths (in micrometers) in SCML2 KO and codepleted PrimPol HCT116 cells complemented with SCML2A^WT^, SCML2A^S267E^, or SCML2B^WT^ and subjected to the indicated experimental conditions (bottom). *n* = 3. (**D**) Schematic of nondenaturing CldU staining analysis with 2.5 μM JH-RE-06 (top). Percentage of U2OS cells containing >3 CldU foci per nucleus after overexpressing PrimPol or treatment with the indicated siRNAs complemented or not with SCML2A^WT^ or SCML2A^S267E^ (bottom). *n* = 3. *P < 0.05, **P < 0.01, ***P < 0.001, and ****P < 0.0001.

Consistently, ssDNA gap accumulation was also observed in JH-RE-06–treated SCML2-KO cells, and this accumulation was rescued by PrimPol depletion (Fig. 5C, lanes 1, 2, and 6). Overexpression of SCML2A WT in SCML2-KO cells, but not SCML2A S267E or SCML2B, inhibited this ssDNA gap accumulation (Fig. 5C, lanes 1 to 5). The same results were also observed by native ssDNA staining (Fig. 5D). Furthermore, the effect of SCML2 depletion on ssDNA formation was comparable to that of PrimPol overexpression after JH-RE-06 treatment (Fig. 5D, lanes 1 to 3, and fig. S5D). SCML2 WT, but not the SCML2 S267E mutant, effectively suppressed ssDNA formation in JH-RE-06–treated SCML2-depleted cells (Fig. 5D, lanes 3 to 5). These results demonstrate that loss of SCML2A-mediated inhibition of PrimPol enhances PrimPol engagement during unstressed DNA replication, thereby increasing ssDNA gaps formation in SCML2-depleted cells. These ssDNA gaps are subsequently filled by the REV1/POLζ complex and RAD51-mediated TS.

### SCML2 depletion potentiates JH-RE-06 or B02 cytotoxicity through PrimPol-mediated ssDNA gap accumulation

On the basis of the data described above, treatment with JH-RE-06 or B02 led to increased ssDNA gaps in SCML2-depleted cells, suggesting that these agents may exhibit preferential toxicity toward SCML2-deficient cells. To test this hypothesis, we assessed the effects of JH-RE-06 or B02 on HeLa and U2OS cells. SCML2-depleted cells displayed increased dose-dependent sensitivity to JH-RE-06 or B02 (Fig. 6, A and B). Codepletion of PrimPol attenuated the enhanced cytotoxicity induced by SCML2 knockdown (Fig. 6, A and B). Furthermore, we analyzed the effect of cotreatment with JH-RE-06 and B02 in SCML2-depleted cells, as dual inhibition of TLS and TS impairs both pathways involved in ssDNA gap filling. SCML2 depletion led to hypersensitivity to cotreatment with JH-RE-06 and B02, and this hypersensitivity was rescued by PrimPol depletion (Fig. 6C). Last, overexpression of SCML2A WT, but not the S267E mutant, rescued SCML2 deficiency–induced sensitivity to JH-RE-06 or B02 (Fig. 6D). These results indicate that the enhanced toxicity of JH-RE-06 or B02 toward SCML2-depleted cells arises from ssDNA gaps generated by PrimPol during DNA replication.

**Fig. 6.**
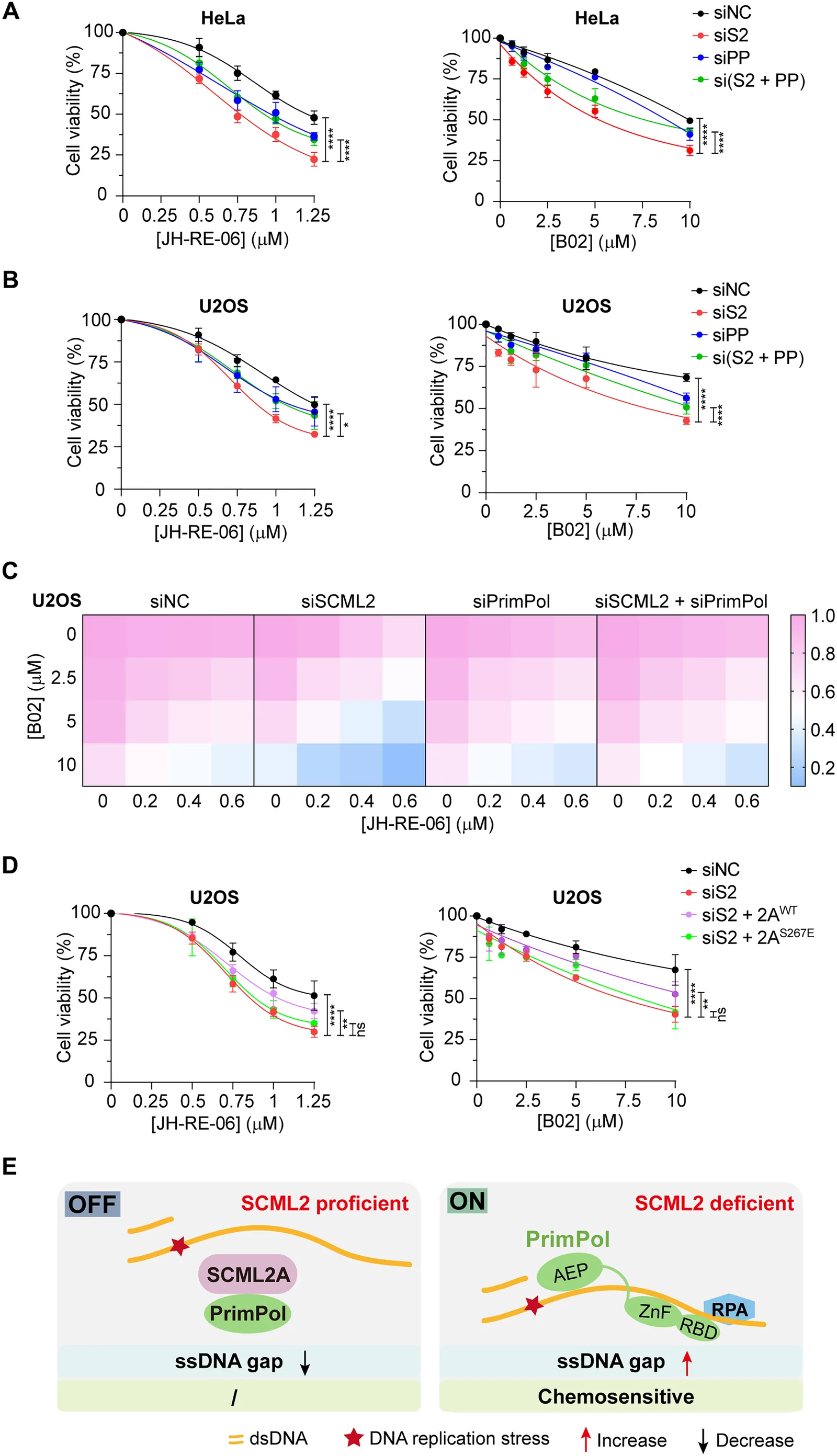
SCML2 depletion potentiates JH-RE-06 or B02 cytotoxicity through PrimPol-mediated ssDNA gap accumulation. (**A** and **B**) Viability of HeLa (A) and U2OS (B) cells transfected with the indicated siRNAs targeting SCML2 and PrimPol after treatment with the JH-RE-06 or B02. The data in panels siNC (A) and siS2 (A) are the same as those in panels siNC and siS2 of fig. S6D. (**C**) Heatmap of the viability of U2OS cells transfected with the indicated SCML2 and PrimPol siRNAs, following combined treatment with JH-RE-06 and B02 at the indicated concentrations. (**D**) Viability of SCML2-depleted U2OS cells complemented with SCML2A^WT^ or SCML2A^S267E^ after treatment with the JH-RE-06 or B02. (**E**) Working model for the mechanisms of SCML2A inhibiting the function of PrimPol. Data are shown as mean ± SD. Significance was calculated by unpaired *t* test. *n* = 3. *P < 0.05, **P < 0.01, and ****P < 0.0001.

Next, we evaluated whether SCML2 depletion influences the cytotoxicity of other agents known to elicit PrimPol-mediated repriming. SCML2 depletion enhanced the cytotoxicity of cisplatin, and this enhancement was rescued by PrimPol depletion (fig. S6A). However, SCML2 depletion had no effect on the cytotoxicity of PARP1 inhibitors (veliparib and talazoparib) (fig. S6, B and C). Accumulation of ssDNA gaps has been observed in BRCA1-deficient cells, which accounts for induced the increased cytotoxicity of REV1 inhibitor (*11*). We compared the cytotoxicity of JH-RE-06 in SCML2-depleted cells with that in BRCA1-depleted cells. SCML2 depletion exerted an effect similar to that of BRCA1 depletion on JH-RE-06 sensitivity, although codepletion of SCML2 and BRCA1 did not further enhance the cytotoxicity (fig. S6, D and E). Thus, SCML2 is a promising therapeutic target for potentiating the cytotoxic effects of ssDNA gap–related compounds (Fig. 6E).

### CDK2-mediated phosphorylation of SCML2A Ser^267^ disrupts its inhibitory effect on PrimPol

Last, we examined how SCML2A-mediated inhibition of PrimPol is modulated. SCML2 Ser^267^ is essential for the SCML2-PrimPol interaction and has been reported as a major phosphorylation site (*35*), indicating that SCML2A S267 phosphorylation may be involved in the regulation of SCML2A-mediated inhibition of PrimPol. Thus, we set out to identify the kinase responsible for SCML2 Ser^267^ phosphorylation. A phospho-specific antibody against SCML2 Ser^267^ phosphorylation was generated. This antibody specifically recognized Ser^267^ phosphorylation on both SCML2A and SCML2B, and this recognition was abolished by Lambda phosphatase treatment (fig. S7A). Sequence analysis showed that Ser^267^ resides within a motif predicted to be a CDK substrate (Fig. 7A) (*60*). Treatment with the CDK inhibitor Roscovitine or K03861, but not RO3306, decreased Ser^267^ phosphorylation (Fig. 7A). Given that Roscovitine and K03861 inhibit CDK2, while low-dose RO3306 selectively inhibits CDK1 (*61*–*64*), these results indicate that CDK2 phosphorylates SCML2 on Ser^267^. As CDK2 forms functional complexes with cyclin A and cyclin E, we evaluated the effects of CDK2/cyclin E1 and CDK2/cyclin A2 on SCML2 Ser^267^ phosphorylation. Overexpression of cyclin E1 or cyclin A2 increased Ser^267^ phosphorylation, and this increase was inhibited by K03861 treatment (Fig. 7B). We also conducted in vitro kinase assays using the CDK2–cyclin E complex. CDK2-cyclin E kinase phosphorylated SCML2A WT but not the S267A mutant (Fig. 7C). Furthermore, the Wee1 inhibitor MK-1775 up-regulates CDK2 and CDK1 activity (*65*) and increases SCML2A S267 phosphorylation. This effect was blocked by the CDK2 inhibitors Roscovitine and K03861, but not CDK1 inhibitor RO3306 (fig. S7B). These data demonstrate that CDK2 is responsible for SCML2 Ser^267^ phosphorylation. Notably, cyclin E1 overexpression decreased the interaction between SCML2 and PrimPol (Fig. 7D). Together, we identified CDK2 as the specific kinase responsible for SCML2 Ser^267^ phosphorylation, which regulates the interaction between SCML2A and PrimPol. Notably, both S267E (phosphomimetic) and S267A (nonphosphorylatable) disrupt SCML2A-PrimPol binding (Fig. 3E and fig. S3B), suggesting that Ser^267^ is essential for this interaction; however, the mechanistic basis for the convergent phenotype of these two structurally opposing mutations remains unresolved and is addressed in Discussion.

**Fig. 7.**
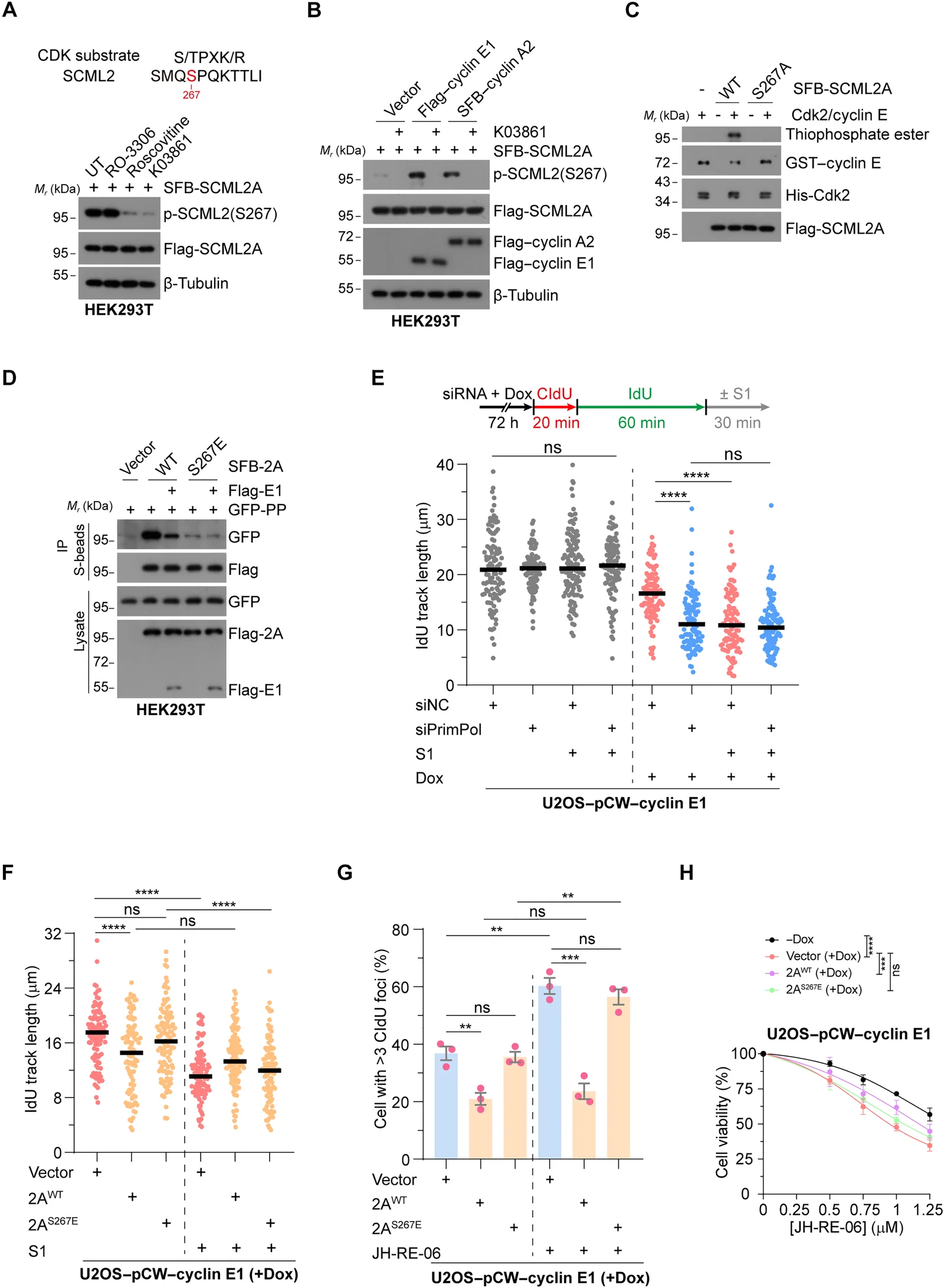
CDK2-mediated phosphorylation of SCML2A Ser^267^ disrupts its inhibitory effect on PrimPol. (**A**) The CDK active site phosphorylates full consensus motifs (S/TPXK/R) and diagram of predicted SCML2 motifs phosphorylated by CDK (top). Following transient transfection of HEK293T cells with plasmid SFB-SCML2A for 24 hours and subsequent treatment with 1 μM RO-3306, 20 μM Roscovitine, or 10 μM K03861 for 24 hours, the phosphorylation level at site Ser^267^ of SCML2A was analyzed by Western blotting (bottom). *n* = 4. (**B**) Western blot analysis of SCML2A phosphorylation at the Ser^267^ site in cells overexpressing cyclin A2 or cyclin E1, with or without 10 μM K03861 treatment for 24 hours. *n* = 3. (**C**) In vitro kinase assay of Cdk2/cyclin E. SFB-SCML2A-WT or SFB-SCML2A(S267A) protein was pulled down through S-beads from HEK293T cells. These beads were incubated with Cdk2/cyclin E proteins as indicated. The phosphorylation of SCML2A was then determined by Western blot analysis. *n* = 3. (**D**) Co-IP analysis of the effect of cyclin E1 on the interaction between SCML2A^WT^ or SCML2A^S267E^ and PrimPol in HEK293T cells. *n* = 3. (**E**) Schematic of DNA fiber tract analysis (top). Dot plot and median of ldU tract lengths (in micrometers) in U2OS–cyclin E1 Tet-On cell lines subjected to the indicated experimental conditions (bottom). *n* = 3. (**F**) Dot plot and median of ldU tract lengths (in micrometers) in U2OS–cyclin E1 Tet-On cell lines overexpressed SCML2A^WT^ or SCML2A^S267E^ after treatment with or without S1. *n* = 3. (**G**) Percentage of U2OS–cyclin E1 Tet-On cell lines overexpressed SCML2A^WT^ or SCML2A^S267E^ containing >3 CldU foci per nucleus after treatment with or without 2.5 μM JH-RE-06. *n* = 3. (**H**) Viability of the U2OS–cyclin E1 Tet-On cell lines overexpressed SCML2A^WT^ or SCML2A^S267E^ after treatment with the JH-RE-06. *n* = 3. **P < 0.01, ***P < 0.001, and ****P < 0.0001.

While CCNE1 amplification has been detected in a subset of tumors and causes ssDNA gap accumulation, the mechanism underlying the formation of these gaps remains unclear (*66*). Cyclin E1 overexpression did not affect the mRNA levels of PrimPol or other DDT-related genes (fig. S7C). As CDK2/cyclin E1 can disrupt the interaction between SCML2A and PrimPol, we speculated that ssDNA gap formation in cyclin E1–overexpressing cells was due to the loss of SCML2A-mediated inhibition of PrimPol. To test this, we used a Tet-on inducible system to overexpress cyclin E1 in U2OS cells (fig. S7D). Cyclin E1 overexpression decreased DNA replication speed (fig. S7E) and led to accumulation of ssDNA gaps, and this accumulation was mitigated by PrimPol depletion (Fig. 7E). The same results were observed in HCC1569 cells, a CCNE1-amplified cancer cell line (fig. S7F). SCML2 depletion did not further increase ssDNA gap formation in cyclin E1–overexpressing cells (fig. S7, F and G), implying that cyclin E1 overexpression induced the loss of SCML2A-mediated inhibition of PrimPol. This effect was corroborated by native ssDNA staining (fig. S7H). Furthermore, JH-RE-06 treatment enhanced ssDNA accumulation in cyclin E1–overexpressing cells, and this enhancement was rescued by PrimPol depletion (fig. S7H). Cyclin E1 overexpression also increased the cytotoxicity of JH-RE-06, and this effect was PrimPol dependent (fig. S7I). PrimPol-mediated ssDNA accumulation was also observed in HCC1569 cells (fig. S7J). Thus, we conclude that up-regulation of PrimPol-mediated repriming accounts for ssDNA accumulation in cyclin E1–overexpressing cells. This phenomenon may result from reduced SCML2A-mediated inhibition of PrimPol upon cyclin E1 overexpression.

To further test the hypothesis that SCML2A S267 hyperphosphorylation in cyclin E1–overexpressing cells induces up-regulation of PrimPol activity, we compared the effect of SCML2A WT and SCML2A S267E overexpression on ssDNA gap formation. Compared with SCML2A S267E, SCML2A WT overexpression partially retained the inhibition of PrimPol in cyclin E1 overexpression cells, as determined by the interaction between SCML2A and PrimPol (Fig. 7D). Overexpression of SCML2A WT, but not S267E, reduced both DNA replication speed and ssDNA accumulation in cyclin E1–overexpressing cells (Fig. 7, F and G). Similarly, SCML2A WT, but not S267E, rescued the enhanced cytotoxicity induced by cyclin E1 overexpression (Fig. 7H). These results demonstrate that cyclin E1 overexpression disrupts SCML2A-mediated inhibition of PrimPol, thereby contributing to ssDNA gap formation.

## DISCUSSION

Over the past decade, numerous studies have established the critical role of PrimPol-mediated repriming in DDT. In this study, we identify SCML2A as a negative regulator of PrimPol through direct interaction. SCML2A inhibits PrimPol-mediated repriming under both DNA replication stress and unstressed DNA replication process. We reveal that SCML2A plays a critical role in balancing PrimPol engagement during unstressed DNA replication. Under unperturbed DNA replication conditions, the increase in PrimPol activity caused by SCML2 depletion drives ssDNA gap accumulation, which is exacerbated upon exposure to REV1 or RAD51 inhibitors. CDK2-mediated phosphorylation of SCML2A at Ser^267^, which is augmented in cyclin E1–overexpressing cells, negatively regulates the interaction between SCML2A and PrimPol. Hyperphosphorylation of SCML2A at Ser^267^ induces up-regulation of PrimPol activity, which, in turn, leads to ssDNA gap accumulation and susceptibility to REV1 or RAD51 inhibitors.

### How SCML2 inhibit PrimPol

PrimPol is regulated through multiple mechanisms, including phosphorylation (*24*, *25*), gene expression (*11*, *15*, *67*), protein stability (*22*), and context-specific chromatin recruitment (*68*). In addition, factors that regulate PrimPol activity via direct binding have been reported, such as p21 (*27*) and PolDIP2 (*69*). For p21, both its direct association with PrimPol and the underlying regulatory mechanism remain unclear (*27*, *45*). PolDIP2 interacts with the AEP domain of PrimPol and enhances nucleotide binding and DNA synthesis by PrimPol (*69*, *70*). Furthermore, fork reversal restricts PrimPol-mediated repriming in response to multiple genotoxic treatments such as UV-C, cisplatin, and HU. Several factors, such as BRCA2-MCM10, can indirectly regulate PrimPol through fork reversal. However, direct suppressors of PrimPol remain poorly characterized.

In the present study, we identify SCML2A as an inhibitory factor of PrimPol, and this prevents the ssDNA-binding capacity of the PrimPol AEP domain through directly binding to PrimPol. SCML2A associates with PrimPol through two distinct regions: The RBR domain of SCML2A binds the C-terminal ZF and RBD domain of PrimPol, while the SAM domain is essential for the association between SCML2A and the N-terminal AEP domain of PrimPol. Both interactions are indispensable for the inhibitory effect of SCML2A on PrimPol. In vitro binding assays confirmed a strong interaction between the C terminus of PrimPol and the RBR domain of SCML2. The RBR domain was first reported as a disordered RBR enriched in positively charged residues, whereas the C-terminal ZF and RBD of PrimPol carry negative charges. Thus, the flexible, positively charged RBR domain may wrap around the negatively charged C-terminal region of PrimPol, mediating their strong interaction (fig. S4E). Our data further demonstrate that Ser^267^ within the RBR domain is essential for this interaction. Notably, both the S267E phosphomimetic mutant and the S267A nonphosphorylatable mutant disrupt the association between SCML2A and PrimPol. Given that the RBR domain is a positively charged IDR, we speculate that both the structural flexibility and positive electrostatic potential of the RBR domain are required for its binding to the negatively charged C-terminal region of PrimPol. Phosphorylation at Ser^267^, as well as the S267E mutation, may alter the positively charged surface of the RBR domain, whereas the S267A mutation likely impairs the structural flexibility of this domain. Alternatively, the hydroxyl group of Ser^267^ may be critical for maintaining the proper RBR domain conformation that facilitates binding to PrimPol. Mutation of Ser^267^ to either Glu or Ala may disrupt this optimal conformation.

The existence of the SAM domain in SCML2A enables direct inhibition of PrimPol catalytic activity through preventing ssDNA binding by the AEP domain. This unique interaction pattern between SCML2A and PrimPol fulfills its full inhibitory function on PrimPol, as manifested by the loss of the inhibitory effect of SCML2B. We speculate that RBR-mediated association with PrimPol may be responsible for recognizing PrimPol, which constructs a platform for SAM-mediated direct suppression of AEP activity (Fig. 4F). Furthermore, both the N-terminal AEP domain and the C-terminal of PrimPol exhibited increased interaction with SCML2A compared with full-length PrimPol (Fig. 4D). As predicted by AlphaFold3, free PrimPol may exist in a closed conformation that can be opened upon RPA binding. The open conformation allows efficient recognition by the RBR domain of SCML2A and subsequent SAM domain–mediated interaction with and suppression of the AEP domain.

On the basis of these findings, we proposed a model for SCML2A-mediated inhibition of PrimPol (Fig. 4H). Free PrimPol may be in a closed conformation with its N- and C-terminal staying near with each other. This closed form exhibits weak affinity for SCML2A. When PrimPol interacts with RPA, the closed conformation undergoes a conformational change to an open state, allowing the AEP domain to bind ssDNA. At this stage, SCML2A recognizes the open PrimPol through the RBR domain. The robust interaction between the RBR and the C terminus of PrimPol facilitates SAM domain–mediated interaction with the AEP domain, which inhibits the ssDNA-binding capacity of AEP domain and impairs priming activity. Our model proposes that a balance exists between PrimPol and SCML2A. PrimPol overexpression may overcome the inhibitory effect of endogenous SCML2 on PrimPol, and this effect can be reversed by SCML2A overexpression. Actually, increased repriming activity and ssDNA gap formation induced by PrimPol overexpression have been observed in our data and other reports (*10*, *58*). Furthermore, cisplatin treatment increases PrimPol expression potentially through ATR, while BRCA1 has also been reported to regulate PrimPol levels (*15*, *67*). This up-regulation of PrimPol protein may disrupt the balance between PrimPol and SCML2A and promote PrimPol activity. Thus, the balance between PrimPol and SCML2A is important for PrimPol engagement during DNA replication.

### SCML2 inhibits PrimPol during unstressed DNA replication

PrimPol exhibits biphasic functions in maintaining genome stability. During replication stress, PrimPol facilitates replication fork recovery, which helps complete DNA replication in a timely manner to avoid severe DNA damage (*71*). However, given that the primase activity of PrimPol exhibits low fidelity (*17*, *20*, *21*), tight regulation of PrimPol activity is essential during unstressed DNA replication to maintain replication fidelity. For example, a compacted heterochromatin structure can limit PrimPol activity during replication of heterochromatin regions, which is important for genome stability (*72*). Our study reveals SCML2A as an inhibitor of PrimPol that suppresses PrimPol engagement during unstressed DNA replication process. As replication forks may encounter specific regions that are hard to go through, an important question is how to determine where and when SCML2-mediated PrimPol inhibition should be released in cells without encountering extra stresses. For example, it has been reported that PrimPol is critical for DNA replication when replisomes encounter DNA secondary structures, such as R-loops and telomeres (*73*, *74*). As the SCML2A RBR region has been reported to bind RNA (*34*), the existence of RNA in R-loop or telomeric regions may suppress the recognition of PrimPol by the RBR, which allows increased PrimPol engagement in these regions. Thus, we speculate that SCML2A suppresses PrimPol engagement during unperturbed DNA replication and that this inhibition can be relieved when replication fork encounters specific DNA structures, such as R-loops or telomeres.

During replication stress, SCML2 depletion promotes replication fork recovery by enhancing the activity of PrimPol. However, SCML2 depletion does not further accelerate DNA replication progression under unperturbed DNA replication in cells lacking extra exogenous stresses. We speculate that enhanced PrimPol engagement upon SCML2 depletion does not affect global DNA replication speed in unstressed cells. Consistent with this hypothesis, previous study reported that PrimPol overexpression does not alter DNA replication speed in unstressed DNA replication (*10*).

### Role of SCML2 during DNA replication stress

Our previous report (*41*) showed that SCML2 depletion reduced CHK1 stability at 12 hours after severe DNA replication stress (3 mM HU) (fig. S2E). This effect was not observed following low-dose HU (50 μM) treatment (fig. S2F) or short-term replication stress (fig. S2, D and E). The effect of SCML2 depletion on replication fork progression happened shortly after replication stress (within 1 hour) (Fig. 1B), suggesting that the role of SCML2 in regulating PrimPol may be not related to its function on CHK1. Combining our previous findings with the present study, we speculate that SCML2 maintains genome stability upon replication stress through distinct mechanisms: (i) SCML2 helps sustain the fidelity of genome duplication by restricting PrimPol activity under short-term or low-level replication stress; (ii) SCML2 promotes genome stability via enhancing CHK1 stability under high-level replication stress. Severe replication stress triggers ssDNA accumulation, which may abrogate the inhibitory effect of SCML2 on PrimPol through competing with PrimPol for binding to the RBR region. In parallel, we speculate that SCML2-mediated stabilization of CHK1 after long-term replication stress may also enhance PrimPol activity via CHK1-mediated S255 phosphorylation. However, this speculation was not validated in this study and needs to be studied in the future. Thus, SCML2 may exert biphasic effects on replication stress and genome stability.

### SCML2 is a promising target for cancer therapy

Replication stress and the accumulation of ssDNA gaps are increasingly emerging as key predictors of genome stability and chemotherapy response in cancer. Overexpression or increased engagement of PrimPol during DNA replication stress can lead to ssDNA gap accumulation. In BRCA-deficient tumors, failure to properly repair ssDNA gaps through RAD51-mediated TS has been associated with increased chemosensitivity to S-phase–targeting cancer therapies. PARP1 inhibitor treatment can further increase ssDNA gaps through disrupting lagging strand maturation, trapping PARP on DNA, and decreasing fork reversal (*10*, *71*, *75*). Here, we found that SCML2 depletion elicits the formation of PrimPol-mediated ssDNA gaps, establishing SCML2 as another promising ssDNA gap–related therapeutic target. We found that SCML2 depletion increased the cytotoxicity of the REV1 inhibitor JH-RE-06 or the RAD51 inhibitor B02, both of which inhibit the ssDNA gap–filling process. The enhanced cytotoxicity of JH-RE-06 upon SCML2 depletion is fully dependent on PrimPol, whereas this is not observed for B02. This discrepancy may result from the multiple roles of RAD51 in replication stress, including ssDNA gap filling via TS (*71*), maintenance of reversed replication fork stability (*76*, *77*), abasic site protection (*78*), and prevention of DNA overreplication (*79*). Meanwhile, SCML2 depletion also leads to hypersensitivity to cisplatin, a chemical reported to induce cell death through repriming-associated ssDNA gap accumulation (*10*). Furthermore, the REV1 inhibitor exhibited comparable cytotoxicity in both BRCA1-depleted and SCML2-depleted cells, confirming that SCML2 is a promising therapeutic target that may exhibit synthetic lethal effect with the REV1 inhibitor. Our previous study also indicated that SCML2 depletion can increase cellular sensitivity to other DNA damage agents (*41*), further supporting SCML2 as a promising target for cancer therapy.

## MATERIALS AND METHODS

### Cell culture

The cell lines used in this study are listed in table S3. All cell lines were cultured in Dulbecco’s modified Eagle’s medium (Gibco) except for HCC1569 cells, which were maintained in RPMI medium, supplemented with 10% fetal bovine serum (Gibco) and 1% penicillin-streptomycin (100 U/ml, Gibco), and maintained at 37°C in a humidified atmosphere containing 5% CO_2_ (*41*).

### Transient transfection and siRNA transfection

HEK293T cells were transfected using polyethylenimine (1 mg/ml; PEI) with plasmid/transfection reagent at a 1:3 ratio when cells reached 40 to 60% confluency. HCT116 and U2OS cells were transfected with Lipofectamine 2000 or Lipofectamine 3000 according to the manufacturer’s instructions. The SFB vector used in this study contains three tags: S, Flag, and SBP tags (*41*). The pEGFP-C1 vector contains a green fluorescent protein tag. The pGEX-4 T-1 vector contains a GST tag. The pET-28a vector contains a His tag.

For siRNA knockdown, cells were transfected with 20 nM siRNA using Lipofectamine RNAiMAX reagent following the manufacturer’s protocol when cells reached ∼30 to 40% confluency. After 24 hours of siRNA incubation, the cells were replaced with fresh medium (*41*). Specific siRNA sequences are detailed in table S1.

### Western blot analysis

Cells were harvested, washed with phosphate-buffered saline (PBS), and then lysed in NETN420 buffer [20 mM tris-HCl (pH 8.0), 420 mM NaCl, 0.5% NP-40, 50 mM NaF, 1.5 mM KH_2_PO_4_, 1 mM EDTA, and protease inhibitors] with phosphatase inhibitors on ice for 20 min. The supernatant was then collected by centrifugation (15 min, 15,000 rpm, 4°C). Protein concentrations were measured using the BCA protein assay kit. For immunoprecipitation, cell lysates were incubated with 10 μl of S agarose beads (2 hours, 4°C). The beads were washed four times with wash buffer [20 mM tris-HCl (pH 8.0), 150 mM NaCl, 0.5% NP-40, 50 mM NaF, 1 mM EDTA, and protease inhibitors]. After boiling the samples in 3× SDS loading buffer at 95°C for 10 min, equal amounts of protein samples were separated by SDS–polyacrylamide gel electrophoresis (SDS-PAGE), transferred onto polyvinylidene difluoride membranes, and visualized with the antibodies listed in table S3 (*41*). All uncropped original Western blots used in the paper are in the Supplementary Materials.

### Quantitative real-time PCR

Total RNA was extracted with RNAiso Plus reagent (TaKaRa). RNA was reverse-transcribed to cDNA using the HiScript cDNA synthesis kit. The synthesized cDNA was used for quantitative real-time polymerase chain reaction (PCR). Real-time PCR was performed using SYBR quantitative PCR (qPCR) mix on a CFX96 instrument (Bio-Rad) (*41*). For sequences of primer pairs used for reverse transcription (RT)–qPCR in this study, see table S2.

### DNA fiber analysis and S1 nuclease treatment

Cells were labeled first with 25 μM CldU followed by 250 μM IdU for the indicated time, with or without 5 μM CD437, 50 μM HU, 2.5 μM JH-RE-06, or 25 μM B02. DNA fiber assays were carried out as described previously (*80*). Labeled cells were collected and lysed on glass slides in spreading buffer [200 mM tris (pH 7.5), 0.5% SDS, and 50 mM EDTA]. The DNA was denatured and incubated with primary antibodies for 1 hour, followed by fluorescence-conjugated secondary antibodies for 1 hour at 37°C. Fibers were visualized using an Olympus IX83 microscope and analyzed using Fiji ImageJ software, with at least 100 fibers examined per condition. The bars on top of the distribution clouds indicate the median.

S1 nuclease digestion was conducted as previously described (*49*). Immediately after an IdU pulse, cells were treated with CSK100 buffer (100 mM NaCl, 3 mM MgCl_2_, 300 mM sucrose, 10 mM Mops, and 0.5% Triton X-100) for 10 min at RT, followed by incubation in S1 nuclease buffer [50 mM NaCl, 5% glycerol, 30 mM sodium acetate (pH 4.6), and 10 mM zinc acetate] with or without S1 nuclease (20 U/ml) for 30 min at 37°C. Cells were then scraped into cold PBS and centrifuged for 5 min at 7000 rpm. Subsequent steps were carried out as described above. All drugs are detailed in table S3.

### EMSA

Primase reactions were performed as described previously (*56*) with slight modifications. Each reaction (10 μl) contained 40 mM Hepes (pH 7.4), 50 mM KCI, 1 mM MnCl_2_, 1 mM dithiothreitol (DTT), bovine serum albumin (BSA; 0.1 μg*/*μl), 5% glycerol, 200 nM FAM-labeled DNA substrate (FAM-5′-AAAAAAAAAACCTGAAAAAAAAAA-3′), and 0.25 to 3 μM protein as indicated in the figure legends. The mixtures were incubated at 24°C for 20 min and immediately placed on ice. Samples were loaded onto a 4% native PAGE gel and electrophoresed at 110 V for 1 hour in 0.5× tris-glycine buffer [12.5 mM tris and 96 mM glycine (pH 8.3)].

### Biacore analysis

The binding affinity between SCML2 and PrimPol was detected using Biacore T200 (GE Healthcare) at 25°C. Briefly, purified SCML2 protein was diluted to 10 μg/ml in 10 mM sodium acetate buffer (pH 5.0) and immobilized on a CM5 chip (GE Healthcare) with the standard EDC/NHS coupling method at about 1000 response units. Purified PrimPol protein was diluted to various concentrations (from 0.117 to 30 μM) in the running buffer (10 mM Hepes at pH 7.4, 150 mM NaCl, 3 mM EDTA, and 0.05% Tween 20). Then, PrimPol was used to flow over the chip surface at 30 μl/min, and the real-time response was recorded. Regeneration was achieved using 10 mM glycine-HCl solution at pH 1.5. The binding kinetics were analyzed using a 1:1 binding model with the Biacore Evaluation Software Version 3.2.1 (GE Healthcare). All Biacore experiments were performed three times. The values presented are the mean ± SD of three independent experiments.

### Nondenaturing CldU staining

The cells were labeled with 10 μM CldU. After 48 hours, cells were permeabilized for 1 min, fixed for 20 min, and incubated in blocking solution (1% BSA in PBS) for 1 hour. Subsequently, cells were stained with anti-BrdU/CldU antibody diluted in blocking solution for 1 hour, followed by incubation with fluorescence-conjugated secondary antibodies for 1 hour and 4′,6-diamidino-2-phenylindole for 5 min at RT. CldU foci were visualized using an Olympus IX83 microscope. At least 300 cells per experimental condition were analyzed.

### Protein purification

The full-length PrimPol or its truncations were cloned into pGEX-4 T-1 vector with GST tags, while SCML2-WT, S267E, or the RBR domain deletion (ΔRBR) mutants were cloned into pET-28a vector with His tags. Protein expression of GST-tagged constructs was induced at 28°C for 4 hours with 0.2 mM isopropyl-β-d-thiogalactopyranoside, whereas His-tagged constructs were induced overnight at 16°C. Protein purification followed previously described methods (*41*).

### In vitro kinase assay

For in vitro kinase assay, SFB-SCML2A-WT or SFB-SCML2A(S267A) was transfected with PEI into HEK293T cells. After 24 hours, the cells were collected and lysed in NETN420 buffer with phosphatase inhibitors on ice for 20 min, followed by centrifugation at 4°C and 15,000 rpm for 15 min to collect the supernatant. Subsequently, the cell lysates were incubated with 25 μl of S agarose beads in 4°C for 3 hours. After washing with NETN100, the beads containing 1.2 μg of SFB-SCML2A-WT or SFB-SCML2A(S267A) were incubated with 0.1 μg of Cdk2/cyclin E protein (Sigma-Aldrich, 14-475), 1 mM DTT, and 0.5 mM ATP-γ-S in 20 μl of phosphate kinase buffer (25 mM tris-HCl at pH 7.5, 25 mM KCl, and 5 mM MgCl_2_) for 1 hour at 30°C. The kinase reaction was stopped by adding 0.5 M EDTA. Last, the mixture was incubated with 50 μM p-nitrobenzyl mesylate (PNBM) at room temperature for 2 hours. The samples were subjected to Western blot analysis.

### Chromatin fractionation

The cells were harvested, washed with PBS, and lysed in NETN100 buffer [20 mM tris-HCl (pH 8.0), 100 mM NaCl, 0.5% NP-40, 50 mM NaF, 1.5 mM KH_2_PO_4_, 1 mM EDTA, and protease inhibitors] supplemented with phosphatase inhibitors. The lysates were centrifuged, and the supernatant was collected as the soluble fraction. The remaining pellet was washed once with NETN100 buffer, and the nuclei were extracted using NETN420 buffer [20 mM tris-HCl (pH 8.0), 420 mM NaCl, 0.5% NP-40, 50 mM NaF, 1.5 mM KH_2_PO_4_, 1 mM EDTA, and protease inhibitors] containing phosphatase inhibitors. Samples were sonicated for 1 min (2 s on, 3 s off, 20 W). After heating at 95°C for 10 min in 3× SDS loading buffer, equal amounts of protein samples were separated by SDS-PAGE.

### Cell proliferation assays

Cells were seeded at 1000 cells per well per 100 μl in a 96-well plate with a black wall and clear bottom and allowed to adhere for 24 hours before treatment with indicated reagents for varying durations as required by the experiment. Following treatment 4 days, CCK8 (10 μl per well) was added to the medium and incubated at 37°C with 5% CO_2_ for 2 to 3 hours. Absorbance was measured at 450 nm using a multimode reader (Spectramax i3x, Molecular Devices).

### Quantification and statistical analysis

All experiments were performed at least three independent biological replicates in the manuscript. For nondenaturing CldU staining, data are shown as mean ± SEM. For RT-qPCR and cell proliferation assays, data were shown as mean ± SD. The significance level was calculated by an unpaired *t* test using GraphPad Prism. For DNA fiber assays, a minimum of 100 fibers was analyzed for each condition (*n* = 3). Bar indicates median. *P* values were calculated by the Mann-Whitney test. *P < 0.05, **P < 0.01, ***P < 0.001, and ****P < 0.0001; ns indicates nonsignificant.

## Supplementary Materials

### This PDF file includes:

Figs. S1 to S7 Tables S1 to S3 Uncropped Western blots

## References

[1] M. Macheret, T. D. Halazonetis, DNA replication stress as a hallmark of cancer. Annu. Rev. Pathol. 10, 425–448 (2015). 10.1146/annurev-pathol-012414-040424 25621662

[2] H. Gaillard, T. Garcia-Muse, A. Aguilera, Replication stress and cancer. Nat. Rev. Cancer 15, 276–289 (2015). 10.1038/nrc3916 25907220

[3] M. Berti, D. Cortez, M. Lopes, The plasticity of DNA replication forks in response to clinically relevant genotoxic stress. Nat. Rev. Mol. Cell Biol. 21, 633–651 (2020). 10.1038/s41580-020-0257-5 32612242

[4] L. Wan, J. Lou, Y. Xia, B. Su, T. Liu, J. Cui, Y. Sun, H. Lou, J. Huang, hPrimpol1/CCDC111 is a human DNA primase-polymerase required for the maintenance of genome integrity. EMBO Rep. 14, 1104–1112 (2013). 10.1038/embor.2013.159 PMC3981091 24126761

[5] S. G. Rudd, L. Glover, S. K. Jozwiakowski, D. Horn, A. J. Doherty, PPL2 translesion polymerase is essential for the completion of chromosomal DNA replication in the African trypanosome. Mol. Cell 52, 554–565 (2013). 10.1016/j.molcel.2013.10.034 PMC3898837 24267450

[6] S. Mourón, S. Rodriguez-Acebes, M. I. Martínez-Jiménez, S. García-Gómez, S. Chocrón, L. Blanco, J. Méndez, Repriming of DNA synthesis at stalled replication forks by human PrimPol. Nat. Struct. Mol. Biol. 20, 1383–1389 (2013). 10.1038/nsmb.2719 24240614

[7] D. Gonzalez-Acosta, E. Blanco-Romero, P. Ubieto-Capella, K. Mutreja, S. Miguez, S. Llanos, F. Garcia, J. Munoz, L. Blanco, M. Lopes, J. Mendez, PrimPol-mediated repriming facilitates replication traverse of DNA interstrand crosslinks. EMBO J. 40, e106355 (2021). 10.15252/embj.2020106355 PMC8280817 34128550

[8] M. J. Peña-Gómez, Y. Rodríguez-Martín, M. del Rio Oliva, Y. Wijesekara Hanthi, S. Berrada, R. Freire, J. Y. Masson, J. C. Reyes, V. Costanzo, I. V. Rosado, HMCES corrupts replication fork stability during base excision repair in homologous recombination-deficient cells. Sci. Adv. 11, eads3227 (2025). 10.1126/sciadv.ads3227 PMC11939059 40138423

[9] A. S. Kawale, X. Ran, P. S. Patel, S. Saxena, M. S. Lawrence, L. Zou, APOBEC3A induces DNA gaps through PRIMPOL and confers gap-associated therapeutic vulnerability. Sci. Adv. 10, eadk2771 (2024). 10.1126/sciadv.adk2771 PMC10798555 38241374

[10] S. Tirman, A. Quinet, M. Wood, A. Meroni, E. Cybulla, J. Jackson, S. Pegoraro, A. Simoneau, L. Zou, A. Vindigni, Temporally distinct post-replicative repair mechanisms fill PRIMPOL-dependent ssDNA gaps in human cells. Mol. Cell 81, 4026–4040.e8 (2021). 10.1016/j.molcel.2021.09.013 PMC8555837 34624216

[11] A. Taglialatela, G. Leuzzi, V. Sannino, R. Cuella-Martin, J. W. Huang, F. Wu-Baer, R. Baer, V. Costanzo, A. Ciccia, REV1-Polζ maintains the viability of homologous recombination-deficient cancer cells through mutagenic repair of PRIMPOL-dependent ssDNA gaps. Mol. Cell 81, 4008–4025.e7 (2021). 10.1016/j.molcel.2021.08.016 PMC8500949 34508659

[12] L. M. Iyer, E. V. Koonin, D. D. Leipe, L. Aravind, Origin and evolution of the archaeo-eukaryotic primase superfamily and related palm-domain proteins: Structural insights and new members. Nucleic Acids Res. 33, 3875–3896 (2005). 10.1093/nar/gki702 PMC1176014 16027112

[13] S. García-Gómez, A. Reyes, M. I. Martínez-Jiménez, E. S. Chocrón, S. Mourón, G. Terrados, C. Powell, E. Salido, J. Méndez, I. J. Holt, L. Blanco, PrimPol, an archaic primase/polymerase operating in human cells. Mol. Cell 52, 541–553 (2013). 10.1016/j.molcel.2013.09.025 PMC3899013 24207056

[14] J. Bianchi, S. G. Rudd, S. K. Jozwiakowski, L. J. Bailey, V. Soura, E. Taylor, I. Stevanovic, A. J. Green, T. H. Stracker, H. D. Lindsay, A. J. Doherty, PrimPol bypasses UV photoproducts during eukaryotic chromosomal DNA replication. Mol. Cell 52, 566–573 (2013). 10.1016/j.molcel.2013.10.035 PMC4228047 24267451

[15] A. Quinet, S. Tirman, J. Jackson, S. Svikovic, D. Lemacon, D. Carvajal-Maldonado, D. Gonzalez-Acosta, A. T. Vessoni, E. Cybulla, M. Wood, S. Tavis, L. F. Z. Batista, J. Mendez, J. E. Sale, A. Vindigni, PRIMPOL-mediated adaptive response suppresses replication fork reversal in BRCA-deficient cells. Mol. Cell 77, 461–474.e9 (2020). 10.1016/j.molcel.2019.10.008 PMC7007862 31676232

[16] L. J. Bailey, J. Bianchi, N. Hégarat, H. Hochegger, A. J. Doherty, PrimPol-deficient cells exhibit a pronounced G2 checkpoint response following UV damage. Cell Cycle 15, 908–918 (2016). 10.1080/15384101.2015.1128597 PMC4889237 26694751

[17] B. A. Keen, S. K. Jozwiakowski, L. J. Bailey, J. Bianchi, A. J. Doherty, Molecular dissection of the domain architecture and catalytic activities of human PrimPol. Nucleic Acids Res. 42, 5830–5845 (2014). 10.1093/nar/gku214 PMC4027207 24682820

[18] O. Rechkoblit, Y. K. Gupta, R. Malik, K. R. Rajashankar, R. E. Johnson, L. Prakash, S. Prakash, A. K. Aggarwal, Structure and mechanism of human PrimPol, a DNA polymerase with primase activity. Sci. Adv. 2, e1601317 (2016). 10.1126/sciadv.1601317 PMC5088642 27819052

[19] T. A. Guilliam, N. C. Brissett, A. Ehlinger, B. A. Keen, P. Kolesar, E. M. Taylor, L. J. Bailey, H. D. Lindsay, W. J. Chazin, A. J. Doherty, Molecular basis for PrimPol recruitment to replication forks by RPA. Nat. Commun. 8, 15222 (2017). 10.1038/ncomms15222 PMC5457501 28534480

[20] M. I. Martínez-Jiménez, S. García-Gómez, K. Bebenek, G. Sastre-Moreno, P. A. Calvo, A. Díaz-Talavera, T. A. Kunkel, L. Blanco, Alternative solutions and new scenarios for translesion DNA synthesis by human PrimPol. DNA Repair 29, 127–138 (2015). 10.1016/j.dnarep.2015.02.013 25746449

[21] T. A. Guilliam, S. K. Jozwiakowski, A. Ehlinger, R. P. Barnes, S. G. Rudd, L. J. Bailey, J. M. Skehel, K. A. Eckert, W. J. Chazin, A. J. Doherty, Human PrimPol is a highly error-prone polymerase regulated by single-stranded DNA binding proteins. Nucleic Acids Res. 43, 1056–1068 (2015). 10.1093/nar/gku1321 PMC4333378 25550423

[22] Y. Yan, Z. Xu, J. Huang, G. Guo, M. Gao, W. Kim, X. Zeng, J. A. Kloeber, Q. Zhu, F. Zhao, K. Luo, Z. Lou, The deubiquitinase USP36 Regulates DNA replication stress and confers therapeutic resistance through PrimPol stabilization. Nucleic Acids Res. 48, 12711–12726 (2020). 10.1093/nar/gkaa1090 PMC7736794 33237263

[23] A. Yoshimura, M. Oikawa, H. Jinbo, Y. Hasegawa, T. Enomoto, M. Seki, WRNIP1 controls the amount of PrimPol. Biol. Pharm. Bull. 42, 764–769 (2019). 10.1248/bpb.b18-00955 31061318

[24] K. P. M. Mehta, V. Thada, R. Zhao, A. Krishnamoorthy, M. Leser, K. Lindsey Rose, D. Cortez, CHK1 phosphorylates PRIMPOL to promote replication stress tolerance. Sci. Adv. 8, eabm0314 (2022). 10.1126/sciadv.abm0314 PMC8967226 35353580

[25] L. J. Bailey, R. Teague, P. Kolesar, L. J. Bainbridge, H. D. Lindsay, A. J. Doherty, PLK1 regulates the PrimPol damage tolerance pathway during the cell cycle. Sci. Adv. 7, eabh1004 (2021). 10.1126/sciadv.abh1004 PMC8641930 34860556

[26] Z. Kang, P. Fu, A. L. Alcivar, H. Fu, C. Redon, T. K. Foo, Y. Zuo, C. Ye, R. Baxley, A. Madireddy, R. Buisson, A. K. Bielinsky, L. Zou, Z. Shen, M. I. Aladjem, B. Xia, BRCA2 associates with MCM10 to suppress PRIMPOL-mediated repriming and single-stranded gap formation after DNA damage. Nat. Commun. 12, 5966 (2021). 10.1038/s41467-021-26227-6 PMC8514439 34645815

[27] P. B. Sang, R. K. Jaiswal, X. Lyu, W. Chai, Human CST complex restricts excessive PrimPol repriming upon UV induced replication stress by suppressing p21. Nucleic Acids Res. 52, 3778–3793 (2024). 10.1093/nar/gkae078 PMC11040158 38348929

[28] S. F. Mansilla, A. P. Bertolin, S. Venerus Arbilla, B. A. Castaño, T. Jahjah, J. K. Singh, S. O. Siri, M. V. Castro, M. B. de la Vega, A. Quinet, L. Wiesmüller, V. Gottifredi, Polymerase iota (Pol ι) prevents PrimPol-mediated nascent DNA synthesis and chromosome instability. Sci. Adv. 9, eade7997 (2023). 10.1126/sciadv.ade7997 PMC10104471 37058556

[29] A. Quinet, S. Tirman, E. Cybulla, A. Meroni, A. Vindigni, To skip or not to skip: Choosing repriming to tolerate DNA damage. Mol. Cell 81, 649–658 (2021). 10.1016/j.molcel.2021.01.012 PMC7935405 33515486

[30] G. Bai, T. Endres, U. Kuhbacher, V. Mengoli, B. H. Greer, E. M. Peacock, M. D. Newton, T. Stanage, M. R. Dello Stritto, R. Lungu, M. P. Crossley, A. Sathirachinda, D. Cortez, S. J. Boulton, P. Cejka, B. F. Eichman, K. A. Cimprich, HLTF resolves G4s and promotes G4-induced replication fork slowing to maintain genome stability. Mol. Cell 84, 3044–3060.e11 (2024). 10.1016/j.molcel.2024.07.018 PMC11366124 39142279

[31] M. D. Palumbieri, C. Merigliano, D. Gonzalez-Acosta, D. Kuster, J. Krietsch, H. Stoy, T. von Kanel, S. Ulferts, B. Welter, J. Frey, C. Doerdelmann, A. Sanchi, R. Grosse, I. Chiolo, M. Lopes, Nuclear actin polymerization rapidly mediates replication fork remodeling upon stress by limiting PrimPol activity. Nat. Commun. 14, 7819 (2023). 10.1038/s41467-023-43183-5 PMC10684888 38016948

[32] G. Bai, C. Kermi, H. Stoy, C. J. Schiltz, J. Bacal, A. M. Zaino, M. K. Hadden, B. F. Eichman, M. Lopes, K. A. Cimprich, HLTF promotes fork reversal, limiting replication stress resistance and preventing multiple mechanisms of unrestrained DNA synthesis. Mol. Cell 78, 1237–1251.e7 (2020). 10.1016/j.molcel.2020.04.031 PMC7305998 32442397

[33] E. Lecona, V. Narendra, D. Reinberg, USP7 cooperates with SCML2 to regulate the activity of PRC1. Mol. Cell. Biol. 35, 1157–1168 (2015). 10.1128/MCB.01197-14 PMC4355532 25605328

[34] R. Bonasio, E. Lecona, V. Narendra, P. Voigt, F. Parisi, Y. Kluger, D. Reinberg, Interactions with RNA direct the Polycomb group protein SCML2 to chromatin where it represses target genes. eLife 3, e02637 (2014). 10.7554/eLife.02637 PMC4074974 24986859

[35] E. Lecona, L. A. Rojas, R. Bonasio, A. Johnston, O. Fernandez-Capetillo, D. Reinberg, Polycomb protein SCML2 regulates the cell cycle by binding and modulating CDK/CYCLIN/p21 complexes. PLOS Biol. 11, e1001737 (2013). 10.1371/journal.pbio.1001737 PMC3866099 24358021

[36] K. Hasegawa, H. S. Sin, S. Maezawa, T. J. Broering, A. V. Kartashov, K. G. Alavattam, Y. Ichijima, F. Zhang, W. C. Bacon, K. D. Greis, P. R. Andreassen, A. Barski, S. H. Namekawa, SCML2 establishes the male germline epigenome through regulation of histone H2A ubiquitination. Dev. Cell 32, 574–588 (2015). 10.1016/j.devcel.2015.01.014 PMC4391279 25703348

[37] M. Luo, J. Zhou, N. A. Leu, C. M. Abreu, J. Wang, M. C. Anguera, D. G. de Rooij, M. Jasin, P. J. Wang, Polycomb protein SCML2 associates with USP7 and counteracts histone H2A ubiquitination in the XY chromatin during male meiosis. PLOS Genet. 11, e1004954 (2015). 10.1371/journal.pgen.1004954 PMC4310598 25634095

[38] S. Maezawa, K. Hasegawa, M. Yukawa, N. Kubo, A. Sakashita, K. G. Alavattam, H. S. Sin, A. V. Kartashov, H. Sasaki, A. Barski, S. H. Namekawa, Polycomb protein SCML2 facilitates H3K27me3 to establish bivalent domains in the male germline. Proc. Natl. Acad. Sci. U.S.A. 115, 4957–4962 (2018). 10.1073/pnas.1804512115 PMC5949012 29686098

[39] L. Du, L. Wang, H. Yang, J. Duan, J. Lai, W. Wu, S. Fan, X. Zhi, Sex comb on midleg Like-2 accelerates hepatocellular carcinoma cell proliferation and metastasis by activating Wnt/β-Catenin/EMT signaling. Yonsei Med. J. 62, 1073–1082 (2021). 10.3349/ymj.2021.62.12.1073 PMC8612862 34816637

[40] J. J. Yang, H. Huang, M. B. Xiao, F. Jiang, W. K. Ni, Y. F. Ji, C. H. Lu, R. Z. Ni, Sex comb on midleg like-2 is a novel specific marker for the diagnosis of gastroenteropancreatic neuroendocrine tumors. Exp. Ther. Med. 14, 1749–1755 (2017). 10.3892/etm.2017.4677 PMC5526161 28810646

[41] Q. Peng, X. Shi, D. Li, J. Guo, X. Zhang, X. Zhang, Q. Chen, SCML2 contributes to tumor cell resistance to DNA damage through regulating p53 and CHK1 stability. Cell Death Differ. 30, 1849–1867 (2023). 10.1038/s41418-023-01184-3 PMC10307790 37353627

[42] A. Maya-Mendoza, P. Moudry, J. M. Merchut-Maya, M. Lee, R. Strauss, J. Bartek, High speed of fork progression induces DNA replication stress and genomic instability. Nature 559, 279–284 (2018). 10.1038/s41586-018-0261-5 29950726

[43] E. Lecona, S. Rodriguez-Acebes, J. Specks, A. J. Lopez-Contreras, I. Ruppen, M. Murga, J. Muñoz, J. Mendez, O. Fernandez-Capetillo, USP7 is a SUMO deubiquitinase essential for DNA replication. Nat. Struct. Mol. Biol. 23, 270–277 (2016). 10.1038/nsmb.3185 PMC4869841 26950370

[44] S. F. Mansilla, A. P. Bertolin, V. Bergoglio, M.-J. Pillaire, M. A. González Besteiro, C. Luzzani, S. G. Miriuka, C. Cazaux, J. S. Hoffmann, V. Gottifredi, Cyclin kinase-independent role of p21^CDKN1A^ in the promotion of nascent DNA elongation in unstressed cells. eLife 5, e18020 (2016). 10.7554/eLife.18020 PMC5120883 27740454

[45] N. L. Calzetta, S. F. Mansilla, C. M. Ahlers, A. P. Bertolin, L. I. Caimi, S. V. Arbilla, J. Zeng, L. Wiesmüller, V. Gottifredi, Endogenous p21 levels protect genomic stability by suppressing both excess and restrained nascent DNA syntheses. Sci. Adv. 11, eadw4618 (2025). 10.1126/sciadv.adw4618 PMC12506965 41061054

[46] I. Klusmann, S. Rodewald, L. Muller, M. Friedrich, M. Wienken, Y. Li, R. Schulz-Heddergott, M. Dobbelstein, p53 activity results in DNA replication fork processivity. Cell Rep. 17, 1845–1857 (2016). 10.1016/j.celrep.2016.10.036 27829155

[47] S. Saxena, L. Zou, Hallmarks of DNA replication stress. Mol. Cell 82, 2298–2314 (2022). 10.1016/j.molcel.2022.05.004 PMC9219557 35714587

[48] A. Quinet, D. Carvajal-Maldonado, D. Lemacon, A. Vindigni, DNA fiber analysis: Mind the Gap! Methods Enzymol. 591, 55–82 (2017). 10.1016/bs.mie.2017.03.019 28645379

[49] A. Quinet, D. J. Martins, A. T. Vessoni, D. Biard, A. Sarasin, A. Stary, C. F. Menck, Translesion synthesis mechanisms depend on the nature of DNA damage in UV-irradiated human cells. Nucleic Acids Res. 44, 5717–5731 (2016). 10.1093/nar/gkw280 PMC4937316 27095204

[50] A. Taglialatela, S. Alvarez, G. Leuzzi, V. Sannino, L. Ranjha, J. W. Huang, C. Madubata, R. Anand, B. Levy, R. Rabadan, P. Cejka, V. Costanzo, A. Ciccia, Restoration of replication fork stability in BRCA1- and BRCA2-deficient cells by inactivation of SNF2-family fork remodelers. Mol. Cell 68, 414–430.e8 (2017). 10.1016/j.molcel.2017.09.036 PMC5720682 29053959

[51] B. Meszaros, G. Erdos, Z. Dosztanyi, IUPred2A: Context-dependent prediction of protein disorder as a function of redox state and protein binding. Nucleic Acids Res. 46, W329–W337 (2018). 10.1093/nar/gky384 PMC6030935 29860432

[52] C. J. Oldfield, A. K. Dunker, Intrinsically disordered proteins and intrinsically disordered protein regions. Annu. Rev. Biochem. 83, 553–584 (2014). 10.1146/annurev-biochem-072711-164947 24606139

[53] P. E. Wright, H. J. Dyson, Intrinsically disordered proteins in cellular signalling and regulation. Nat. Rev. Mol. Cell Biol. 16, 18–29 (2015). 10.1038/nrm3920 PMC4405151 25531225

[54] H. Zhou, S. Di Palma, C. Preisinger, M. Peng, A. N. Polat, A. J. Heck, S. Mohammed, Toward a comprehensive characterization of a human cancer cell phosphoproteome. J. Proteome Res. 12, 260–271 (2013). 10.1021/pr300630k 23186163

[55] N. Dephoure, C. Zhou, J. Villén, S. A. Beausoleil, C. E. Bakalarski, S. J. Elledge, S. P. Gygi, A quantitative atlas of mitotic phosphorylation. Proc. Natl. Acad. Sci. U.S.A. 105, 10762–10767 (2008). 10.1073/pnas.0805139105 PMC2504835 18669648

[56] E. O. Boldinova, A. G. Baranovskiy, D. I. Gagarinskaya, A. A. Manukyan, A. V. Makarova, T. H. Tahirov, The role of catalytic and regulatory domains of human PrimPol in DNA binding and synthesis. Nucleic Acids Res. 51, 7541–7551 (2023). 10.1093/nar/gkad507 PMC10415149 37326028

[57] L. J. Bainbridge, K. Zabrady, A. J. Doherty, Coordination of primer initiation within the catalytic domain of human PrimPol. J. Mol. Biol. 435, 168338 (2023). 10.1016/j.jmb.2023.168338 37923120

[58] A. Nusawardhana, L. M. Pale, C. M. Nicolae, G. L. Moldovan, USP1-dependent nucleolytic expansion of PRIMPOL-generated nascent DNA strand discontinuities during replication stress. Nucleic Acids Res. 52, 2340–2354 (2024). 10.1093/nar/gkad1237 PMC10954467 38180818

[59] J. L. Wojtaszek, N. Chatterjee, J. Najeeb, A. Ramos, M. Lee, K. Bian, J. Y. Xue, B. A. Fenton, H. Park, D. Li, M. T. Hemann, J. Hong, G. C. Walker, P. Zhou, A small molecule targeting mutagenic translesion synthesis improves chemotherapy. Cell 178, 152–159.e11 (2019). 10.1016/j.cell.2019.05.028 PMC6644000 31178121

[60] Z. Songyang, S. Blechner, N. Hoagland, M. F. Hoekstra, H. Piwnica-Worms, L. C. Cantley, Use of an oriented peptide library to determine the optimal substrates of protein kinases. Curr. Biol. 4, 973–982 (1994). 10.1016/s0960-9822(00)00221-9 7874496

[61] L. T. Alexander, H. Mobitz, P. Drueckes, P. Savitsky, O. Fedorov, J. M. Elkins, C. M. Deane, S. W. Cowan-Jacob, S. Knapp, Type II inhibitors targeting CDK2. ACS Chem. Biol. 10, 2116–2125 (2015). 10.1021/acschembio.5b00398 26158339

[62] L. T. Vassilev, C. Tovar, S. Chen, D. Knezevic, X. Zhao, H. Sun, D. C. Heimbrook, L. Chen, Selective small-molecule inhibitor reveals critical mitotic functions of human CDK1. Proc. Natl. Acad. Sci. U.S.A. 103, 10660–10665 (2006). 10.1073/pnas.0600447103 PMC1502288 16818887

[63] L. Meijer, A. Borgne, O. Mulner, J. P. Chong, J. J. Blow, N. Inagaki, M. Inagaki, J. G. Delcros, J. P. Moulinoux, Biochemical and cellular effects of roscovitine, a potent and selective inhibitor of the cyclin-dependent kinases cdc2, cdk2 and cdk5. Eur. J. Biochem. 243, 527–536 (1997). 10.1111/j.1432-1033.1997.t01-2-00527.x 9030781

[64] A. Bakshi, F. E. Iturra, A. Alamban, M. Rosas-Salvans, S. Dumont, M. G. Aydogan, Cytoplasmic division cycles without the nucleus and mitotic CDK/cyclin complexes. Cell 186, 4694–4709.e16 (2023). 10.1016/j.cell.2023.09.010 PMC10659773 37832525

[65] C. R. Elbaek, V. Petrosius, J. Benada, L. Erichsen, R. B. Damgaard, C. S. Sorensen, WEE1 kinase protects the stability of stalled DNA replication forks by limiting CDK2 activity. Cell Rep. 38, 110261 (2022). 10.1016/j.celrep.2021.110261 35045293

[66] S. Nayak, J. A. Calvo, K. Cong, M. Peng, E. Berthiaume, J. Jackson, A. M. Zaino, A. Vindigni, M. K. Hadden, S. B. Cantor, Inhibition of the translesion synthesis polymerase REV1 exploits replication gaps as a cancer vulnerability. Sci. Adv. 6, eaaz7808 (2020). 10.1126/sciadv.aaz7808 PMC7286678 32577513

[67] C. Mellor, J. Nassar, S. Svikovic, J. E. Sale, PRIMPOL ensures robust handoff between on-the-fly and post-replicative DNA lesion bypass. Nucleic Acids Res. 52, 243–258 (2024). 10.1093/nar/gkad1054 PMC10783524 37971291

[68] J. Straka, J. B. Khatib, L. Pale, C. M. Nicolae, G. L. Moldovan, CAF-1 promotes efficient PrimPol recruitment to nascent DNA for single-stranded DNA gap formation. Nucleic Acids Res. 52, 13865–13880 (2024). 10.1093/nar/gkae1068 PMC11662685 39558157

[69] T. A. Guilliam, L. J. Bailey, N. C. Brissett, A. J. Doherty, PolDIP2 interacts with human PrimPol and enhances its DNA polymerase activities. Nucleic Acids Res. 44, 3317–3329 (2016). 10.1093/nar/gkw175 PMC4838387 26984527

[70] K. Kasho, G. Stojkovič, C. Velázquez-Ruiz, M. I. Martínez-Jiménez, M. Doimo, T. Laurent, A. Berner, A. E. Pérez-Rivera, L. Jenninger, L. Blanco, S. Wanrooij, A unique arginine cluster in PolDIP2 enhances nucleotide binding and DNA synthesis by PrimPol. Nucleic Acids Res. 49, 2179–2191 (2021). 10.1093/nar/gkab049 PMC7913696 33533925

[71] L. Falbo, V. Costanzo, Replicative gaps in DNA damage tolerance, genome instability, and cancer therapy. Mol. Cell 86, 1200–1216 (2026). 10.1016/j.molcel.2026.02.018 41864203

[72] V. Gaggioli, C. S. Y. Lo, N. Reverón-Gómez, Z. Jasencakova, H. Domenech, H. Nguyen, S. Sidoli, A. Tvardovskiy, S. Uruci, J. A. Slotman, Y. Chai, J. G. S. C. S. Gonçalves, E. M. Manolika, O. N. Jensen, D. Wheeler, S. Sridharan, S. Chakrabarty, J. Demmers, R. Kanaar, A. Groth, N. Taneja, Dynamic de novo heterochromatin assembly and disassembly at replication forks ensures fork stability. Nat. Cell Biol. 25, 1017–1032 (2023). 10.1038/s41556-023-01167-z PMC10344782 37414849

[73] T. Igarashi, M. Mazevet, T. Yasuhara, K. Yano, A. Mochizuki, M. Nishino, T. Yoshida, Y. Yoshida, N. Takamatsu, A. Yoshimi, K. Shiraishi, H. Horinouchi, T. Kohno, R. Hamamoto, J. Adachi, L. Zou, B. Shiotani, An ATR-PrimPol pathway confers tolerance to oncogenic KRAS-induced and heterochromatin-associated replication stress. Nat. Commun. 14, 4991 (2023). 10.1038/s41467-023-40578-2 PMC10435487 37591859

[74] S. In, P. Renck Nunes, R. Valador Fernandes, J. Lingner, TERRA R-loops trigger a switch in telomere maintenance towards break-induced replication and PRIMPOL-dependent repair. EMBO J. 44, 4525–4551 (2025). 10.1038/s44318-025-00502-4 PMC12361433 40624280

[75] M.-M. Genois, J.-P. Gagne, T. Yasuhara, J. Jackson, S. Saxena, M.-F. Langelier, I. Ahel, M. T. Bedford, J. M. Pascal, A. Vindigni, G. G. Poirier, L. Zou, CARM1 regulates replication fork speed and stress response by stimulating PARP1. Mol. Cell 81, 784–800.e8 (2021). 10.1016/j.molcel.2020.12.010 PMC7897296 33412112

[76] M. Daza-Martin, K. Starowicz, M. Jamshad, S. Tye, G. E. Ronson, H. L. MacKay, A. S. Chauhan, A. K. Walker, H. R. Stone, J. F. J. Beesley, J. L. Coles, A. J. Garvin, G. S. Stewart, T. J. McCorvie, X. Zhang, R. M. Densham, J. R. Morris, Isomerization of BRCA1-BARD1 promotes replication fork protection. Nature 571, 521–527 (2019). 10.1038/s41586-019-1363-4 31270457

[77] Y. Hashimoto, A. Ray Chaudhuri, M. Lopes, V. Costanzo, Rad51 protects nascent DNA from Mre11-dependent degradation and promotes continuous DNA synthesis. Nat. Struct. Mol. Biol. 17, 1305–1311 (2010). 10.1038/nsmb.1927 PMC4306207 20935632

[78] Y. W. Hanthi, M. A. Ramirez-Otero, R. Appleby, A. De Antoni, L. Joudeh, V. Sannino, S. Waked, A. Ardizzoia, V. Barra, D. Fachinetti, L. Pellegrini, V. Costanzo, RAD51 protects abasic sites to prevent replication fork breakage. Mol. Cell 84, 3026–3043.e11 (2024). 10.1016/j.molcel.2024.07.004 39178838

[79] S. Munoz, E. Blanco-Romero, D. González-Acosta, S. Rodriguez-Acebes, D. Megías, M. Lopes, J. Méndez, RAD51 restricts DNA over-replication from re-activated origins. EMBO J. 43, 1043–1064 (2024). 10.1038/s44318-024-00038-z PMC10942984 38360996

[80] J. A. Halliwell, P. Gravells, H. E. Bryant, DNA fiber assay for the analysis of DNA replication progression in human pluripotent stem cells. Curr. Protoc. Stem Cell Biol. 54, e115 (2020). 10.1002/cpsc.115 32584505

