# Supplementary material for: SCML2 inhibits PrimPol-mediated DNA replication repriming and regulates replication gap-associated cancer cell vulnerability
Source: Sci Adv. 2026 Oct 23;12(43):eaee4738. doi: 10.1126/sciadv.aee4738 (PMC13693694; doi:10.1126/sciadv.aee4738)

Science Advances
AAAS

Supplementary Materials for

SCML2 inhibits PrimPol-mediated DNA replication repriming and regulates replication gap-associated cancer cell vulnerability

Xin Shi *et al.*

Corresponding author: Qiang Chen, Chenq124@whu.edu.cn; Xin Shi, shi_xin@whu.edu.cn

Sci. Adv. **12**, eaee4738 (2026)
DOI: 10.1126/sciadv.aee4738

This PDF file includes:

Figs. S1 to S7
Tables S1 to S3
Uncropped Western Blots

Supplemental Figure 1

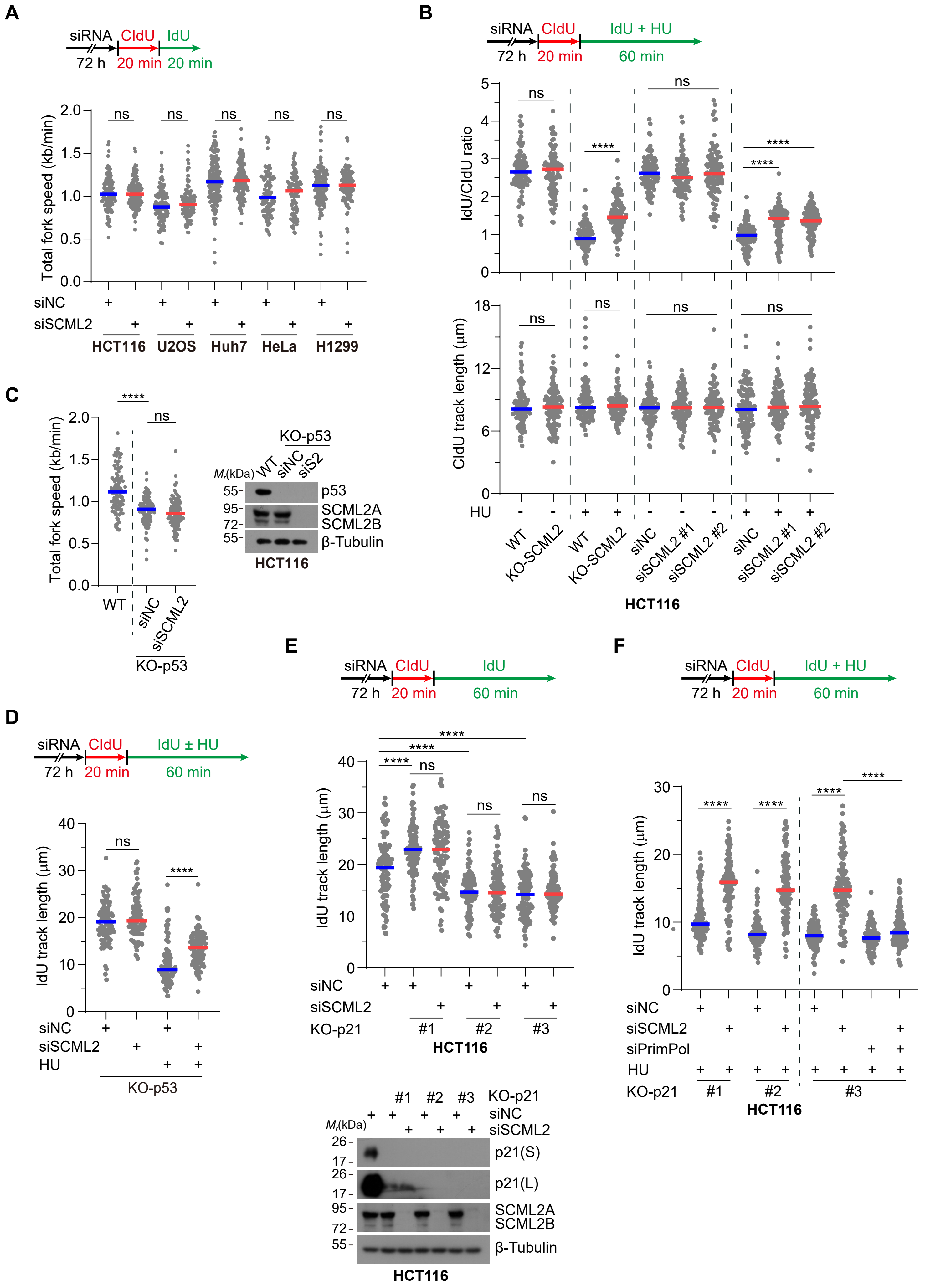

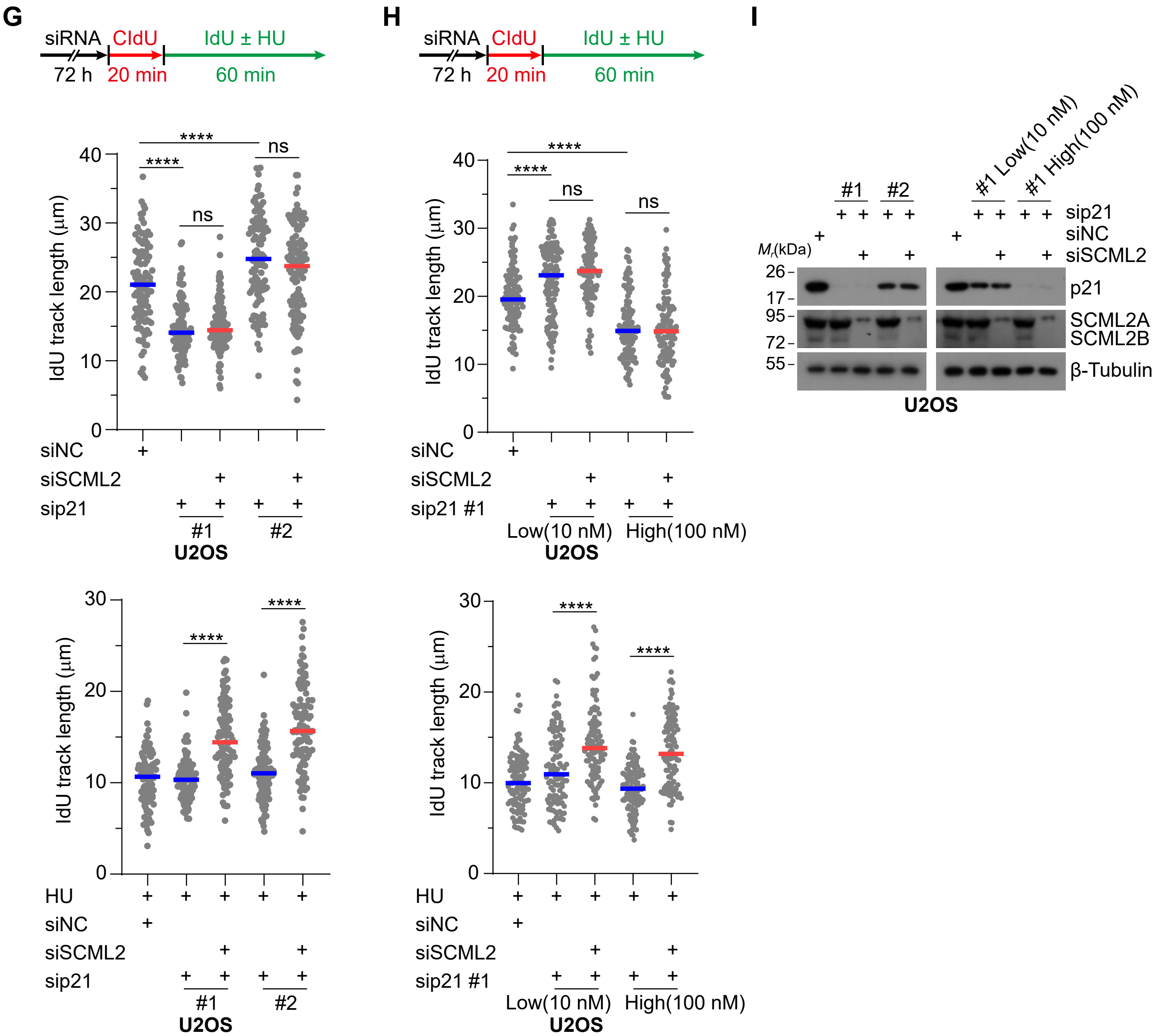
G
siRNA
72 h
CldU
20 min
IdU ± HU
60 min
IdU track length (μm)
****
****
ns
ns
siNC
siSCML2
sip21
#1
#2
U2OS
HU
****
****
H
sip21 #1
Low(10 nM)
High(100 nM)
I
#1 Low(10 nM)
#1 High(100 nM)
sip21
siNC
siSCML2
M_r(kDa)
26
17
95
72
55
p21
SCML2A
SCML2B
β-Tubulin

Supplemental Figure legends

Supplemental Figure 1. SCML2 depletion accelerates nascent DNA synthesis upon DNA replication stress

(A) Schematic of DNA fiber tract analysis (top). Dot plot and median of total fork speed in indicated cells treated with siRNA targeting SCML2 (bottom). n=3.

(B) Dot plot and median of ldU/CldU ratio and CldU track lengths in HCT116 cells subjected to the indicated experimental conditions corresponding to Figure 1A. n=3.

(C) Dot plot and median of total fork speed in HCT116 p53 KO cells treated with siRNA targeting SCML2 (Left). Levels of p53 and SCML2 were analyzed by western blot (Right). n=3.

(D) Dot plot and median of ldU track lengths in HCT116 p53 KO cells with SCML2 deficiency treated with or without 50 μM HU for 60 min (bottom). n=3.

(E and F) Schematic of DNA fiber tract analysis (top). Dot plot and median of ldU track lengths in HCT116 p21 KO cells with SCML2 deficiency treated with (F) or without (E) 50 μM HU for 60 min (middle). Levels of p21 and SCML2 were analyzed by western blot (bottom). n=3.

(G) Schematic of DNA fiber tract analysis (top). Dot plot and median of ldU track lengths in HCT116 cells transfected with siNC or two different sip21 (100 nM). Cells were either left untreated (middle) or exposed to 50 μM HU for 60 min (bottom). n=3.

(H) Schematic of DNA fiber tract analysis (top). Dot plot and median of ldU track lengths in HCT116 cells transfected with siNC or two different concentrations of sip21#1: low (10 nM) and high (100 nM). Cells were either left untreated (middle) or exposed to 50 μM HU for 60 min (bottom). n=3.

(I) Levels of p21 and SCML2 were analyzed by western blot (bottom). n=3.

A minimum of 100 DNA fibers were analyzed for each condition (n=3). Bar: median. p values were calculated by Mann-Whitney test (****$p < 0.0001$, ns, non-significant).

Supplemental Figure 2

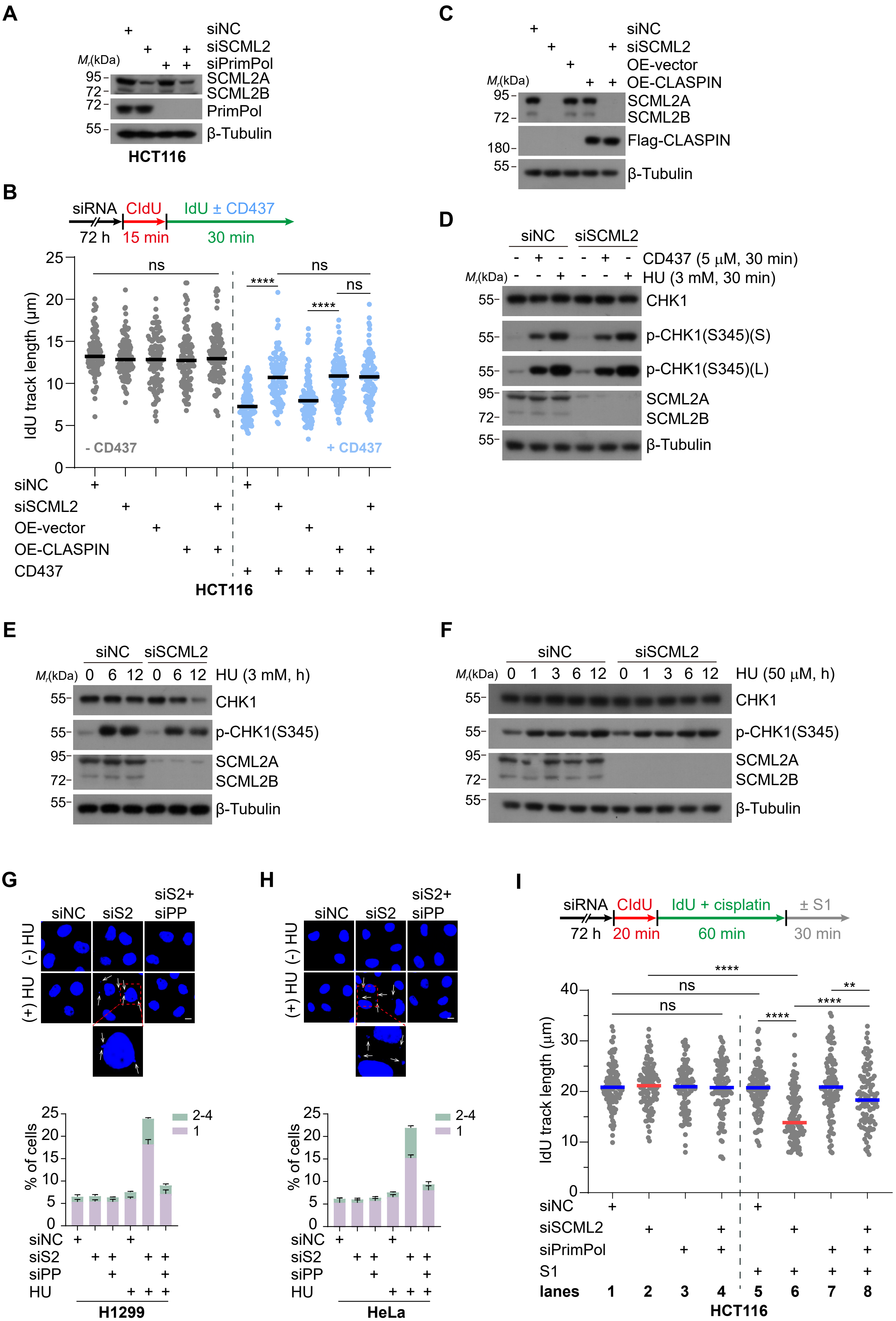

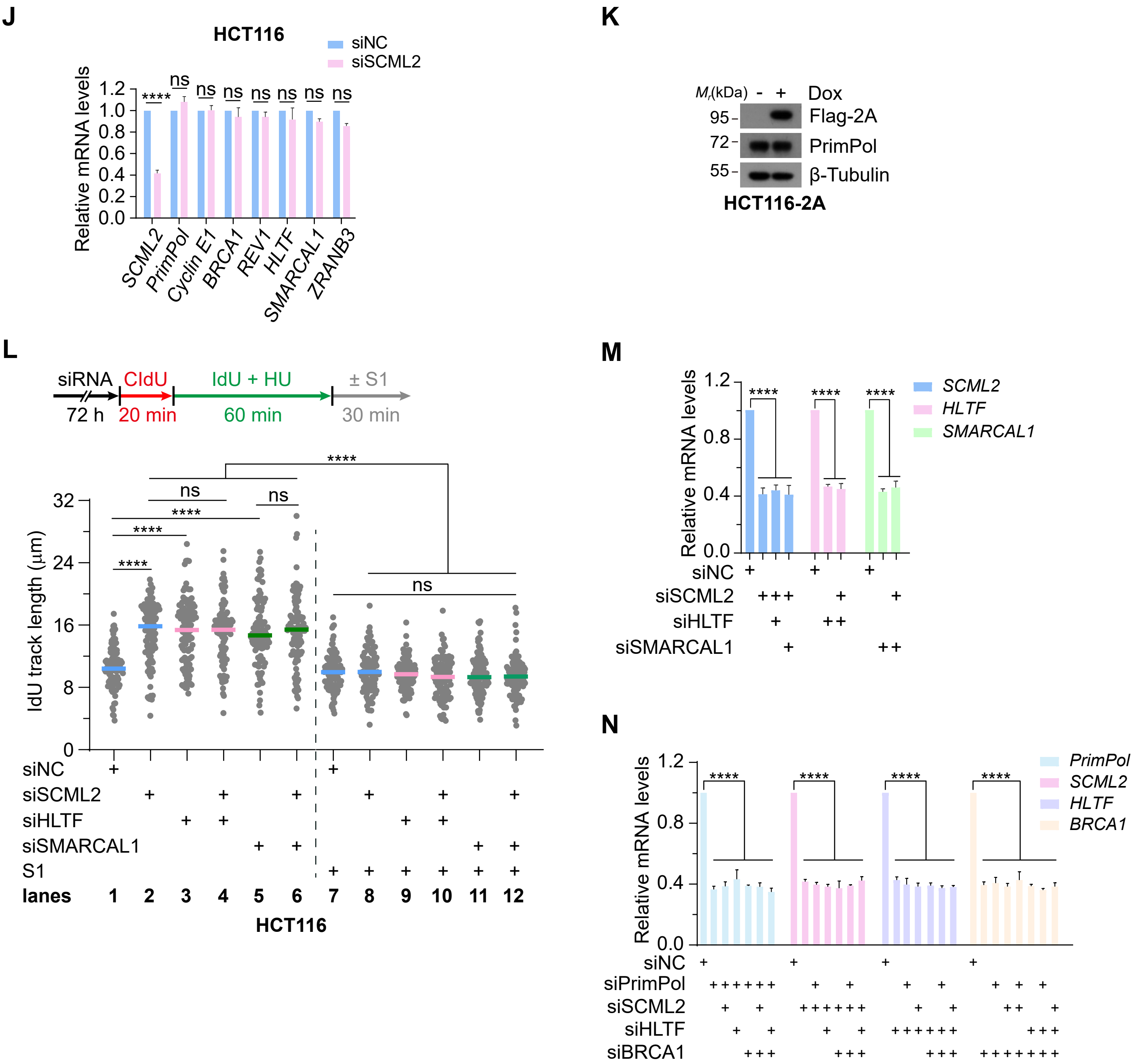
J
HCT116
siNC
siSCML2
Relative mRNA levels
1.2
1.0
0.8
0.6
0.4
0.2
0.0
****
ns
SCML2
PrimPol
Cyclin E1
BRCA1
REV1
HLTF
SMARCAL1
ZRANB3
K
M_r(kDa)
- +
Dox
95
Flag-2A
72
PrimPol
55
β-Tubulin
HCT116-2A
L
siRNA
CldU
IdU + HU
± S1
72 h
20 min
60 min
30 min
IdU track length (μm)
32
24
16
8
0
****
ns
siNC
siSCML2
siHLTF
siSMARCAL1
S1
lanes
1 2 3 4 5 6 7 8 9 10 11 12
HCT116
M
Relative mRNA levels
1.2
0.8
0.4
0.0
****
SCML2
HLTF
SMARCAL1
siNC
siSCML2
siHLTF
siSMARCAL1
N
Relative mRNA levels
1.2
0.8
0.4
0.0
****
PrimPol
SCML2
HLTF
BRCA1
siNC
siPrimPol
siSCML2
siHLTF
siBRCA1

Supplemental Figure 2. SCML2 depletion promotes PrimPol-mediated increase of DNA synthesis after DNA replication stress

(A) Levels of SCML2 and PrimPol were analyzed by western blot in HCT116 cells after treatment with the indicated siRNAs. n=3.

(B) Dot plot and median of ldU track lengths in HCT116 cells exposed to 5 μM CD437 for 30 min after treatment with siRNA targeting SCML2 or CLASPIN overexpression construct. n=3.

(C) Western blot analysis of SCML2 and CLASPIN protein levels in cells from Supplemental Figure 2B. n=3.

(D) Western blot analysis of CHK1 and p-CHK1 (S345) protein levels in cells treated with 5 μM CD437 or 3 mM HU for 30 min. n=3.

(E and F) Western blot analysis of CHK1 and p-CHK1 (S345) protein levels in cells treated with 3 mM HU (E) or 50 μM HU (F) for the indicated time points. n=3.

(G and H) Representative images of micronuclei staining in H1299 cells (G) and HeLa cells (H) exposed HU after treatment with the indicated siRNAs. Scale bar, 10 μm (top). Percentage of the cells containing 1 micronucleus or 2-4 micronuclei per nucleus (bottom). n=3.

(I) Schematic of the IdU/CldU pulse-labeling protocol with 150 μM cisplatin treatment followed by 20 U/mL S1 nuclease digestion (top). Dot plot and median of ldU tract lengths (μm) in HCT116 cells subjected to the indicated experimental conditions (bottom). n=3.

(J) The mRNA levels of the indicated genes in HCT116 cells after treatment with siRNA targeting SCML2. Statistical analysis was conducted by unpaired t test (****$p < 0.0001$, ns, non-significant). n=3.

(K) Levels of SCML2 and PrimPol were analyzed by western blot in HCT116-SCML2A Tet-on cell lines after treatment with 1μg/mL doxycycline (Dox) for 24 h. n=3.

(L) Schematic of the IdU/CldU pulse-labeling protocol with 50 μM HU treatment followed by 20 U/mL S1 nuclease digestion (top). Dot plot and median of ldU tract

lengths (μm) in HCT116 cells subjected to the indicated experimental conditions (bottom). n=3.

(M and N) The mRNA levels of the indicated genes in HCT116 cells from Supplemental Figure 2L (M) and Figure 2G (N). n=3.

A minimum of 100 DNA fibers were analyzed for each condition (n=3). Bar: median. p values were calculated by Mann-Whitney test (**p < 0.01, ****p < 0.0001, ns, non-significant).

Supplemental Figure 3

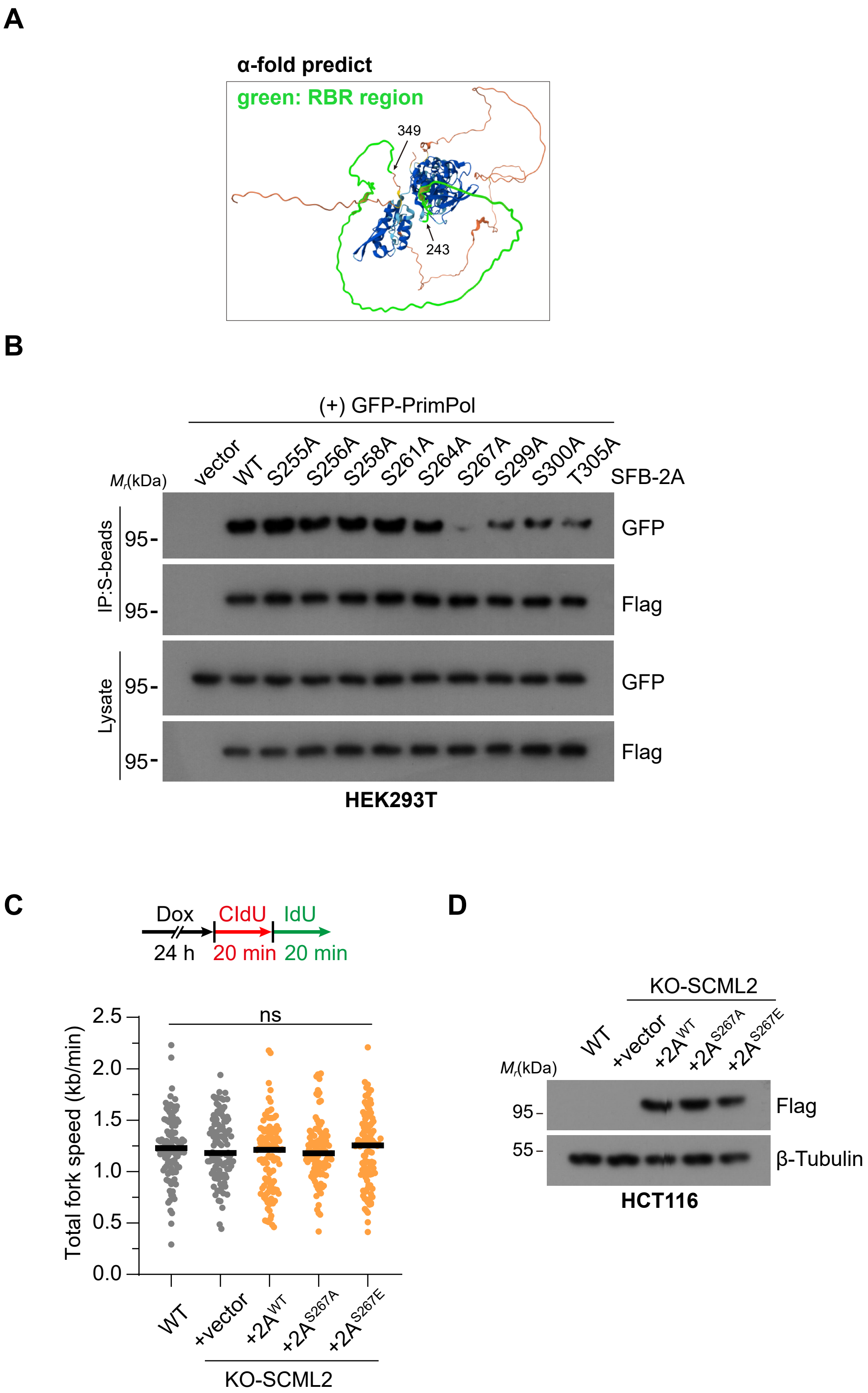

Supplemental Figure 3. SCML2 inhibits PrimPol through RBR domain-mediated interaction

(A) Diagram of predicted SCML2A protein structure from AlphaFold Protein Structure Database (https://alphafold.com/). The green area represents the RBR region.

(B) The interaction of GFP-PrimPol with SFB-SCML2A mutation as shown was detected by Co-IP in HEK293T cells. n=4.

(C) Schematic of DNA fiber tract analysis (top). Dot plot and median of total fork speed in HCT116 WT cells and KO-SCML2 cells overexpressing $SCML2A^{WT}$, $SCML2A^{S267A}$ or $SCML2A^{S267E}$ cells (bottom). n=3.

(D) Levels of SCML2A protein in cells as shown in Figure 3F and Supplemental Figure 3C were analyzed by western blot. n=3.

Supplemental Figure 4

A

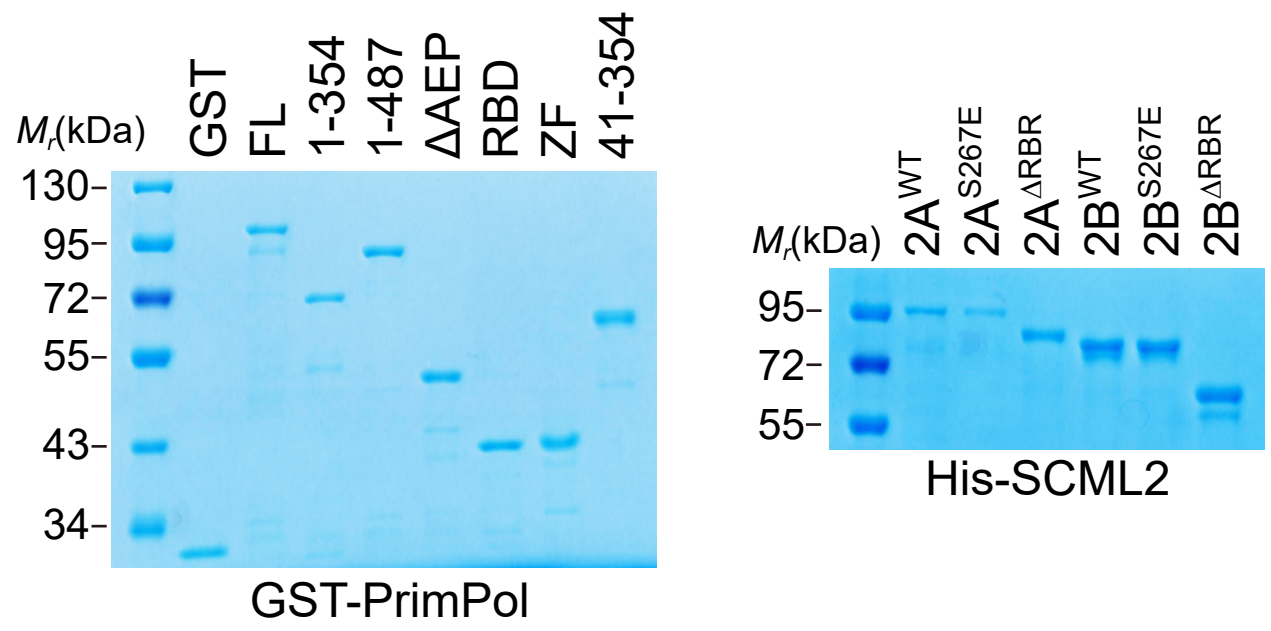

B

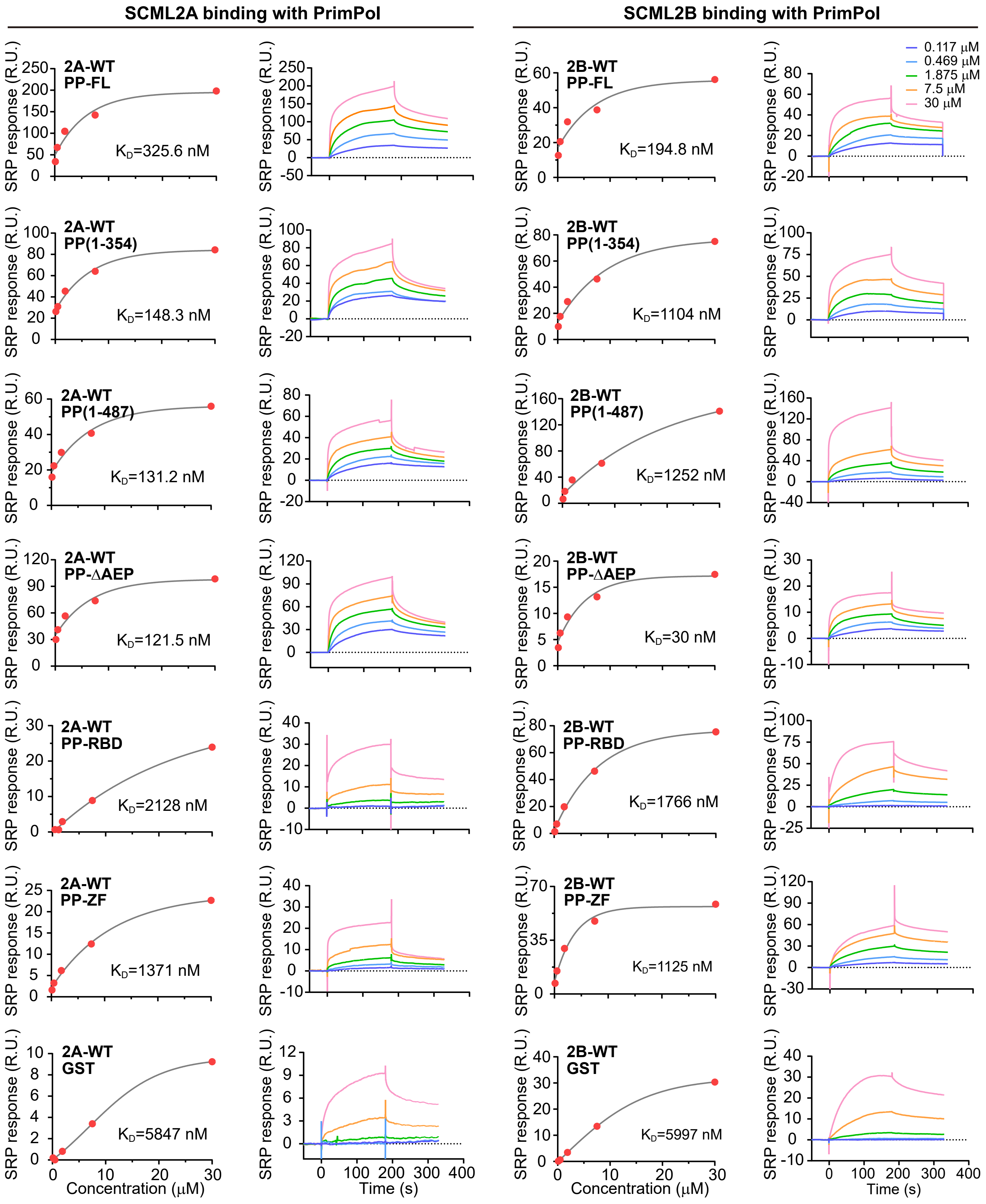

C

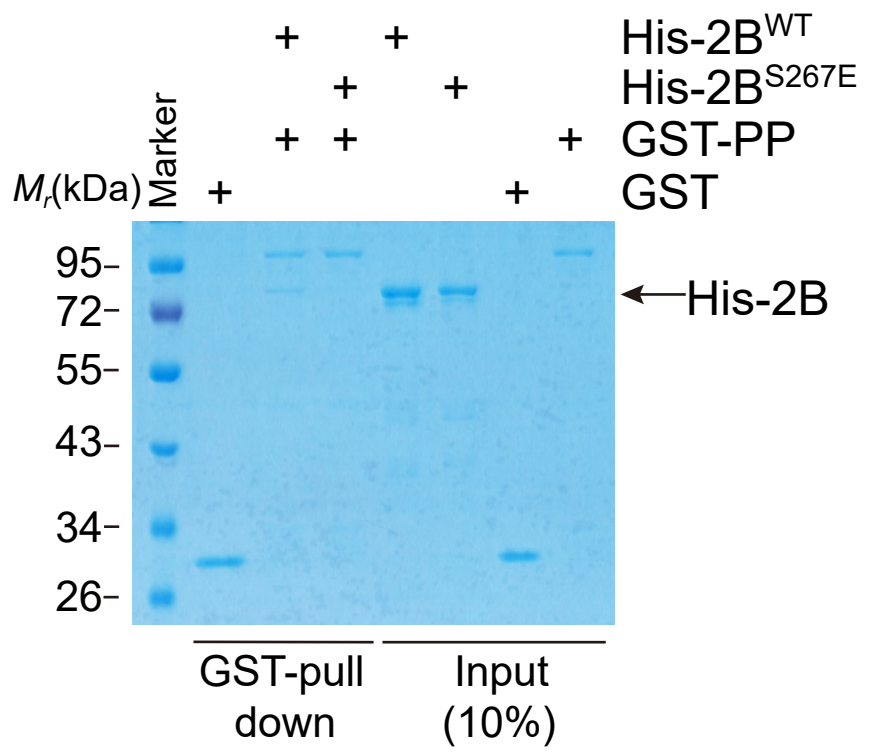

His-2B^WT
His-2B^S267E
GST-PP
GST
Marker
Mr(kDa)
95-
72-
55-
43-
34-
26-
His-2B
GST-pull down
Input (10%)

D

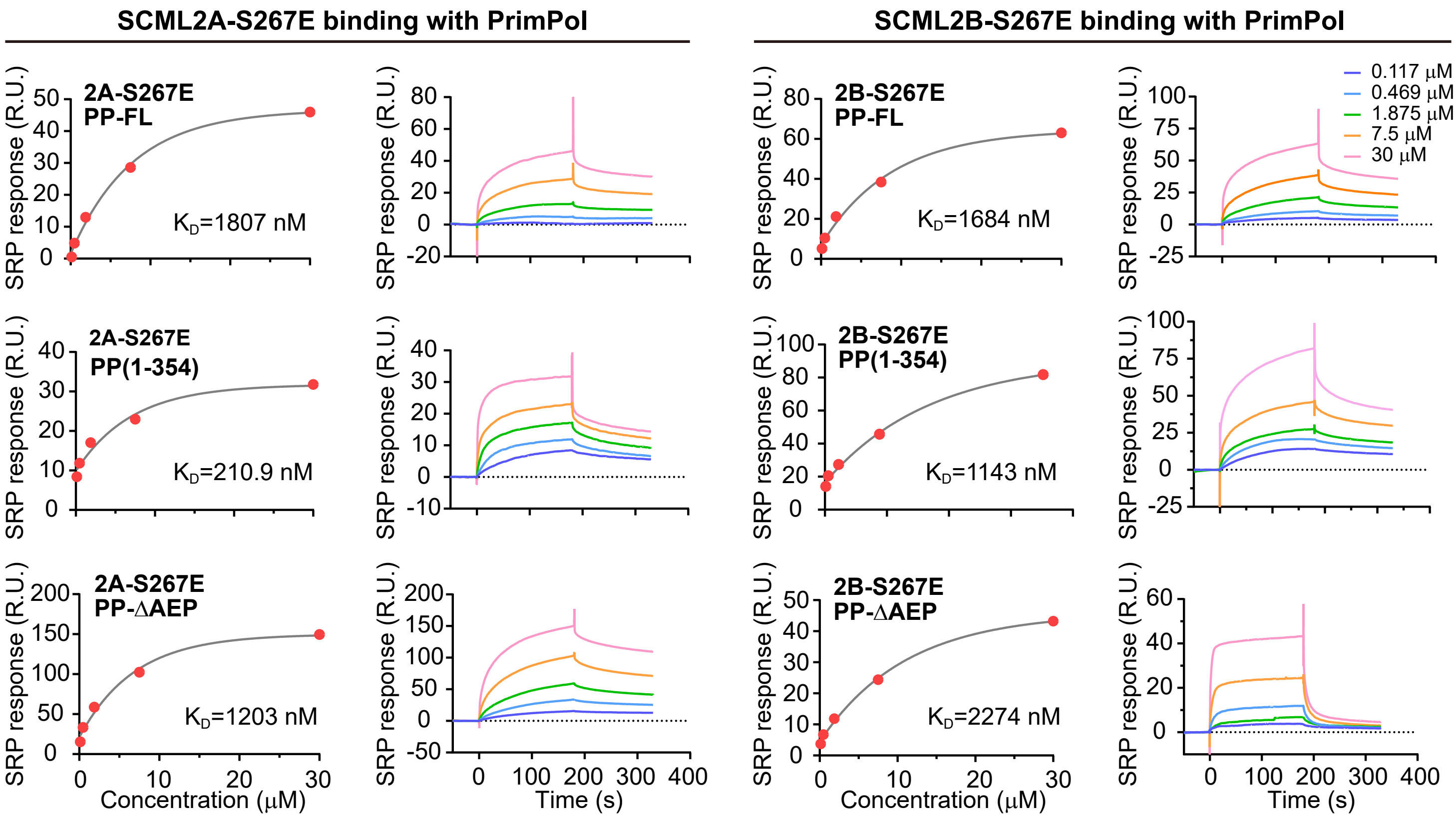

SCML2A-S267E binding with PrimPol
SCML2B-S267E binding with PrimPol
2A-S267E PP-FL
KD=1807 nM
2B-S267E PP-FL
KD=1684 nM
2A-S267E PP(1-354)
KD=210.9 nM
2B-S267E PP(1-354)
KD=1143 nM
2A-S267E PP-ΔAEP
KD=1203 nM
2B-S267E PP-ΔAEP
KD=2274 nM
0.117 μM
0.469 μM
1.875 μM
7.5 μM
30 μM
SRP response (R.U.)
Concentration (μM)
Time (s)

E

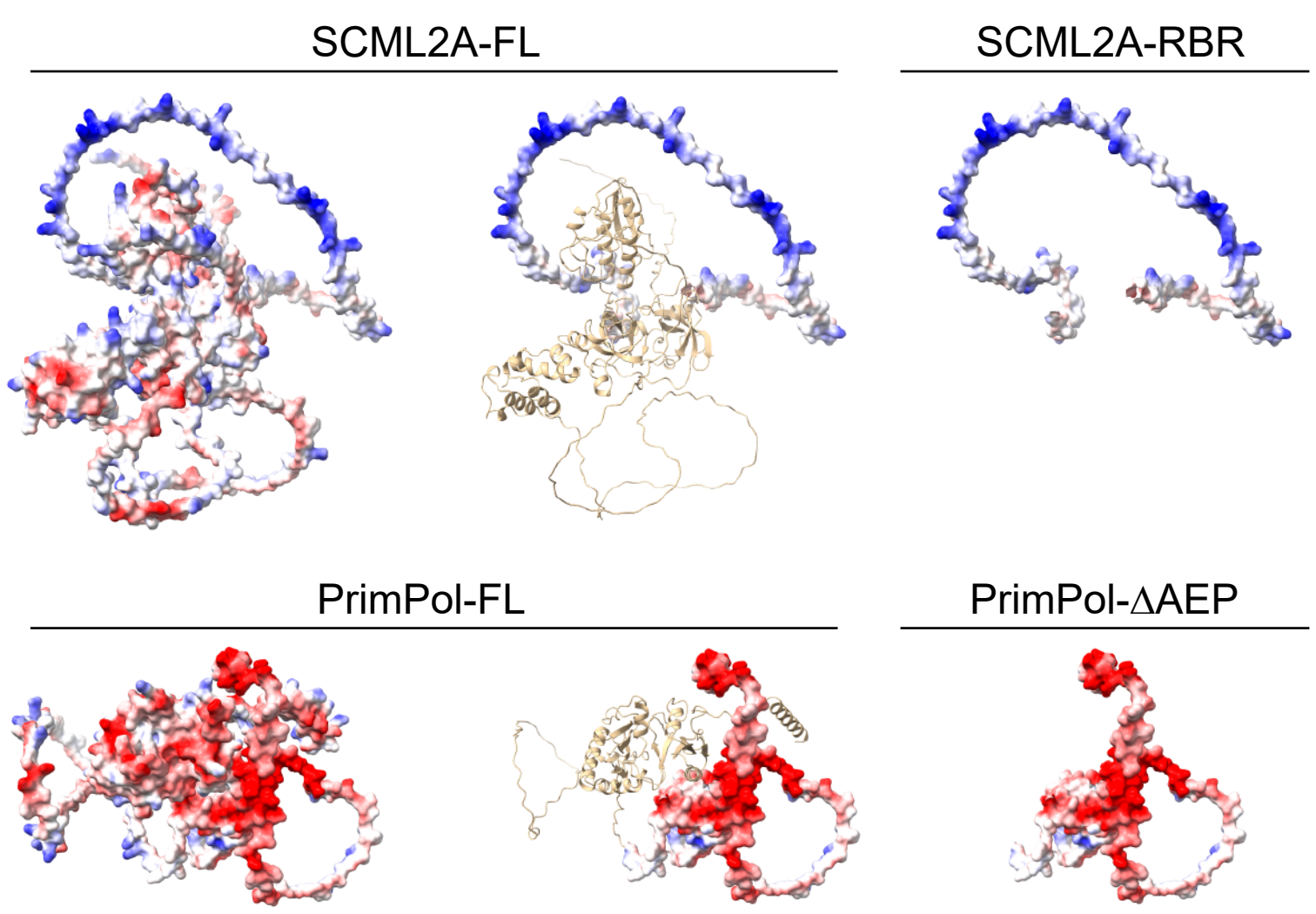

SCML2A-FL
SCML2A-RBR
PrimPol-FL
PrimPol-ΔAEP

F

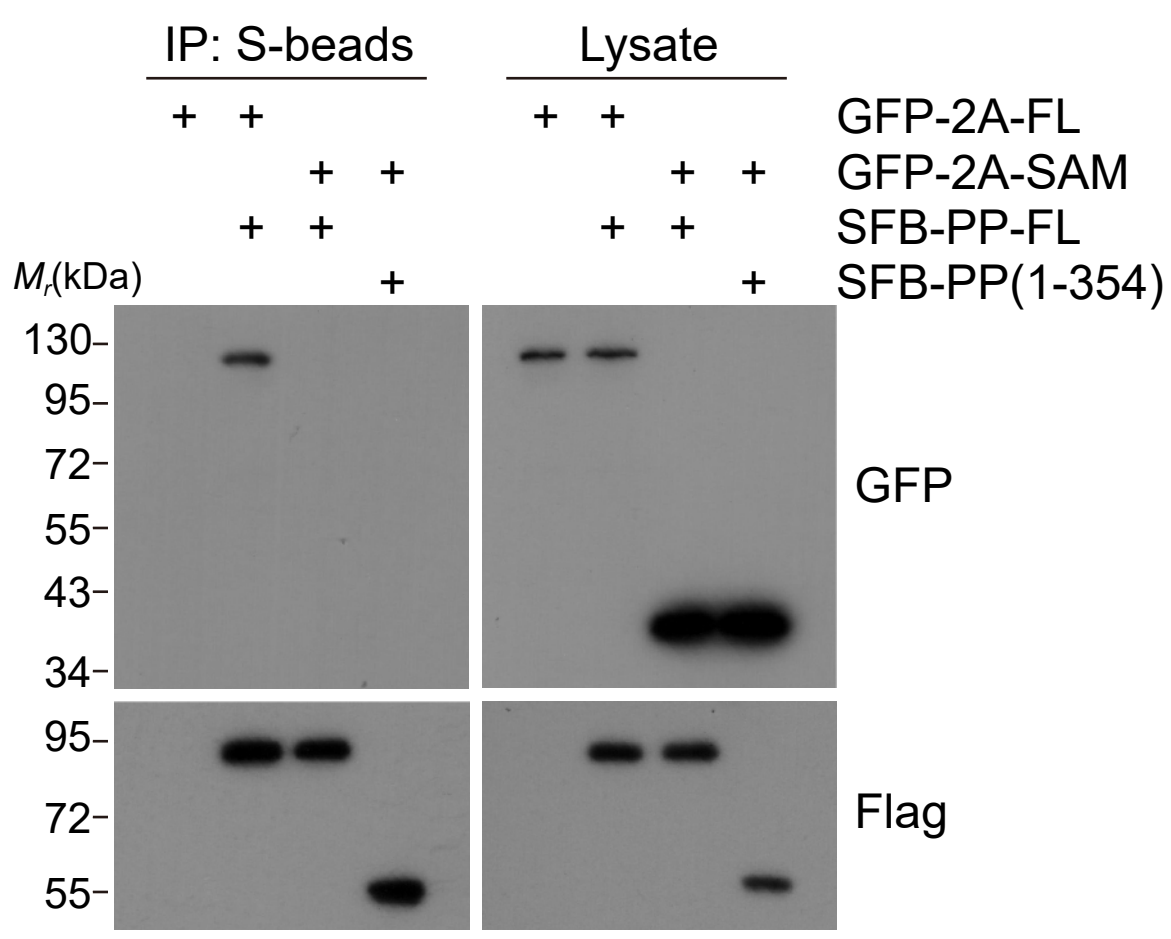

IP: S-beads
Lysate
GFP-2A-FL
GFP-2A-SAM
SFB-PP-FL
SFB-PP(1-354)
Mr(kDa)
130-
95-
72-
55-
43-
34-
95-
72-
55-
GFP
Flag

G

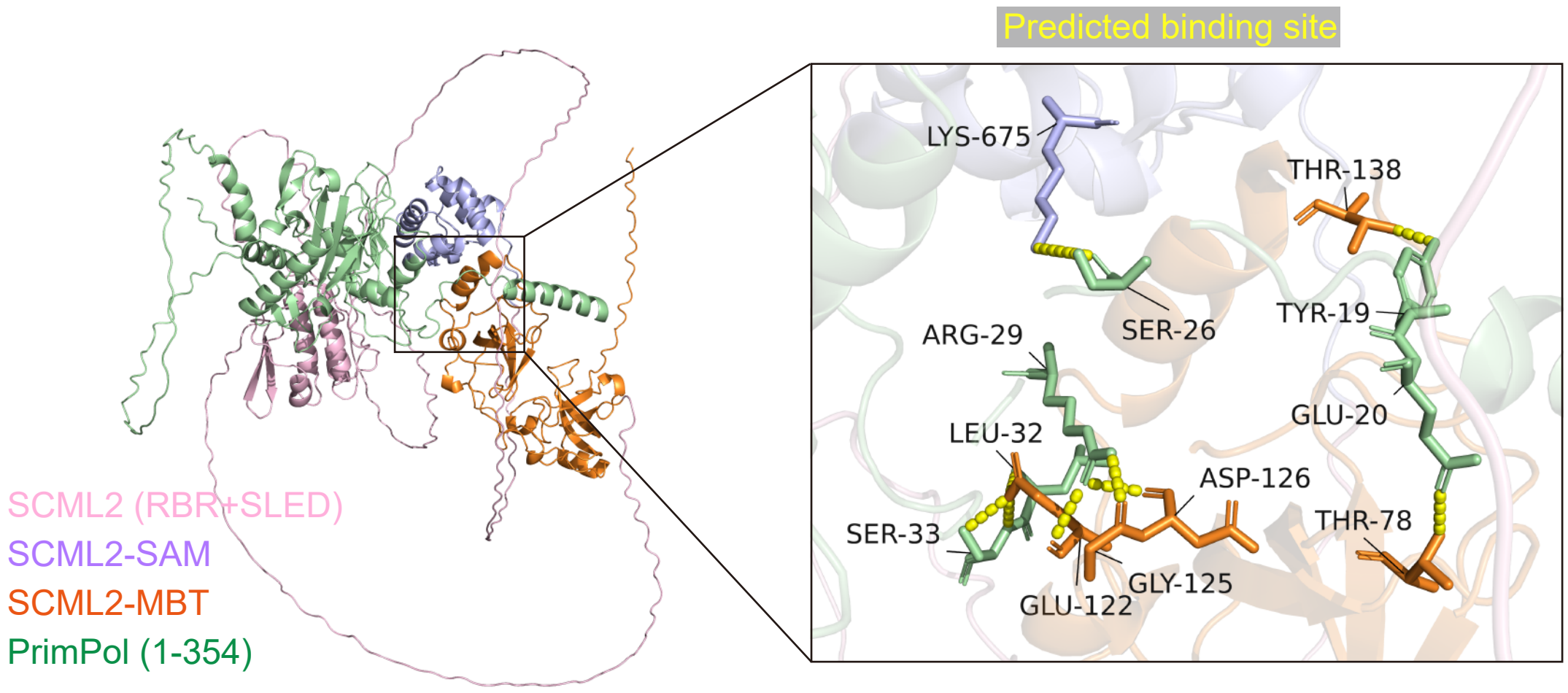
Predicted binding site
LYS-675
THR-138
SER-26
ARG-29
TYR-19
GLU-20
LEU-32
ASP-126
THR-78
SER-33
GLY-125
GLU-122
SCML2 (RBR+SLED)
SCML2-SAM
SCML2-MBT
PrimPol (1-354)

H

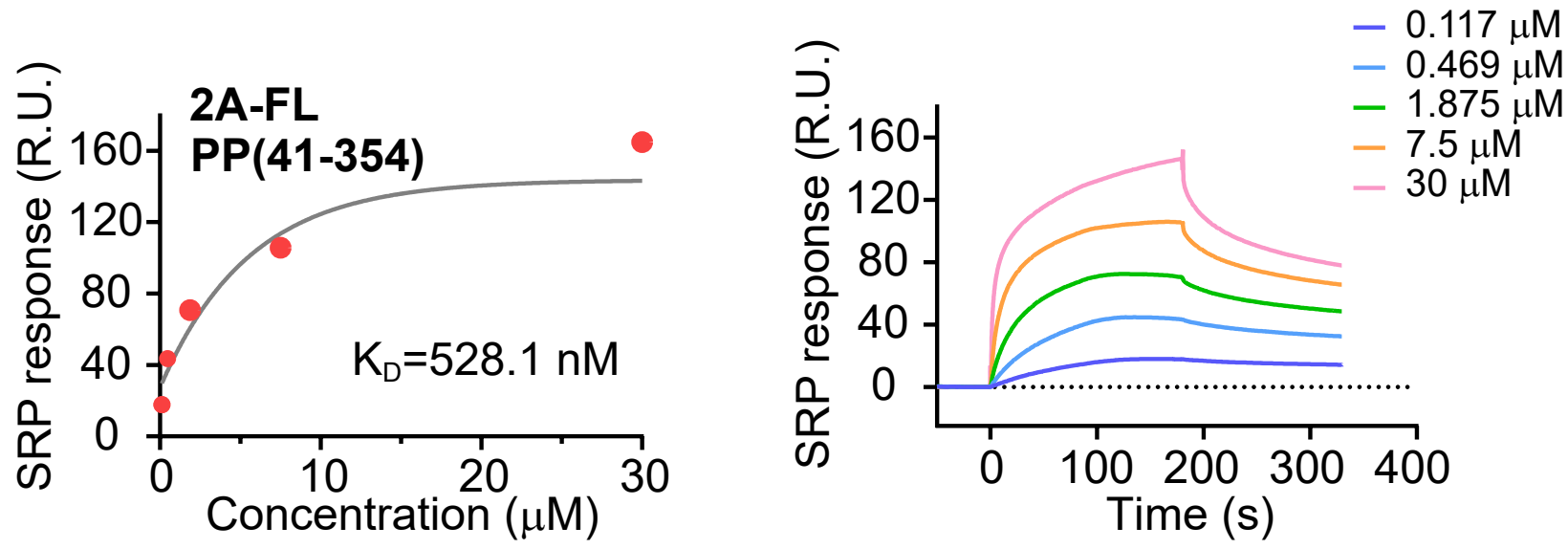
2A-FL
PP(41-354)
K_D=528.1 nM
SRP response (R.U.)
Concentration (μM)
0
10
20
30
0
40
80
120
160
0.117 μM
0.469 μM
1.875 μM
7.5 μM
30 μM
Time (s)
100
200
300
400

I

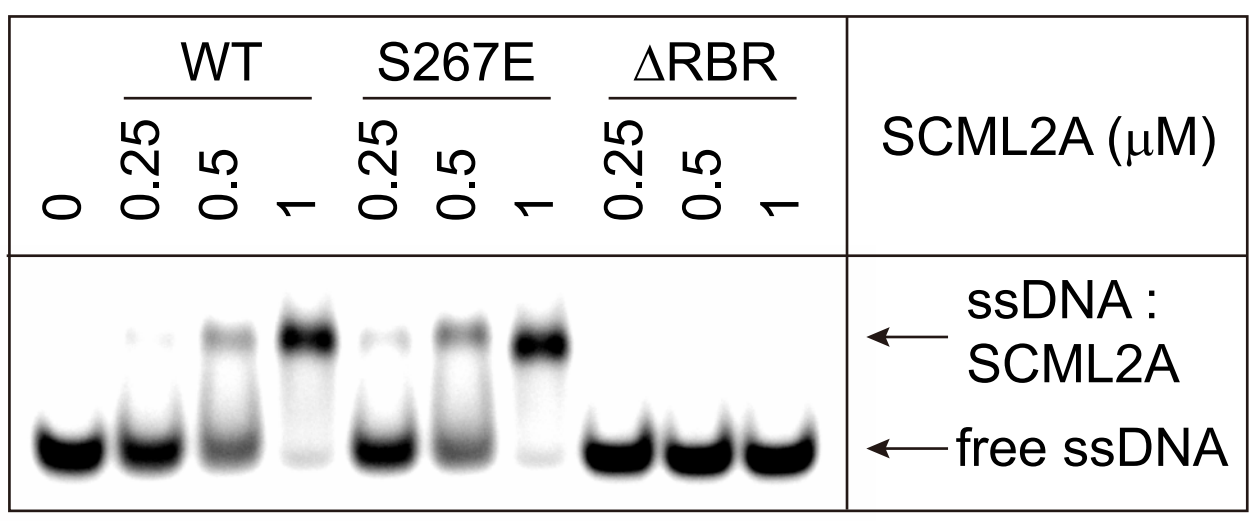
WT
S267E
ΔRBR
0
0.25
0.5
1
SCML2A (μM)
ssDNA : SCML2A
free ssDNA

K

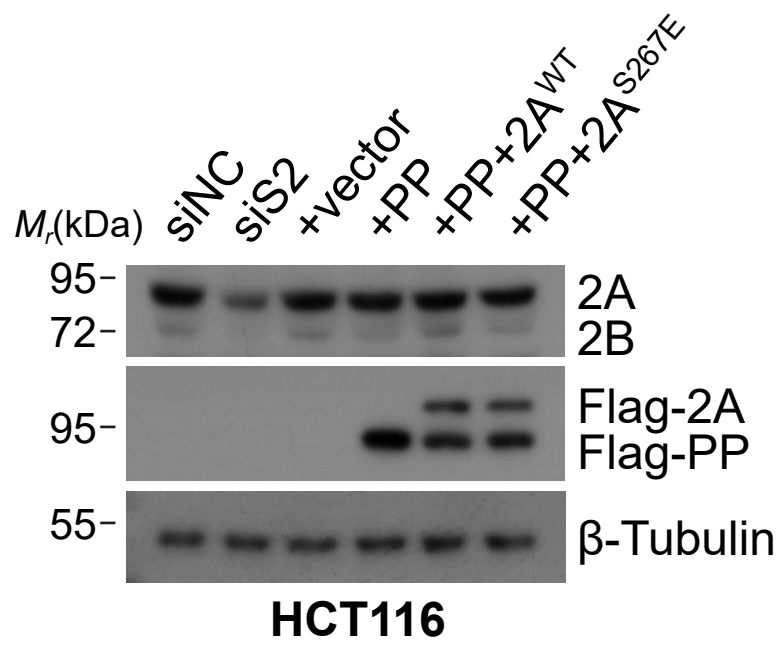
M_r(kDa)
siNC
siS2
+vector
+PP
+PP+2A^{WT}
+PP+2A^{S267E}
95
72
2A
2B
95
Flag-2A
Flag-PP
55
β-Tubulin
HCT116

J

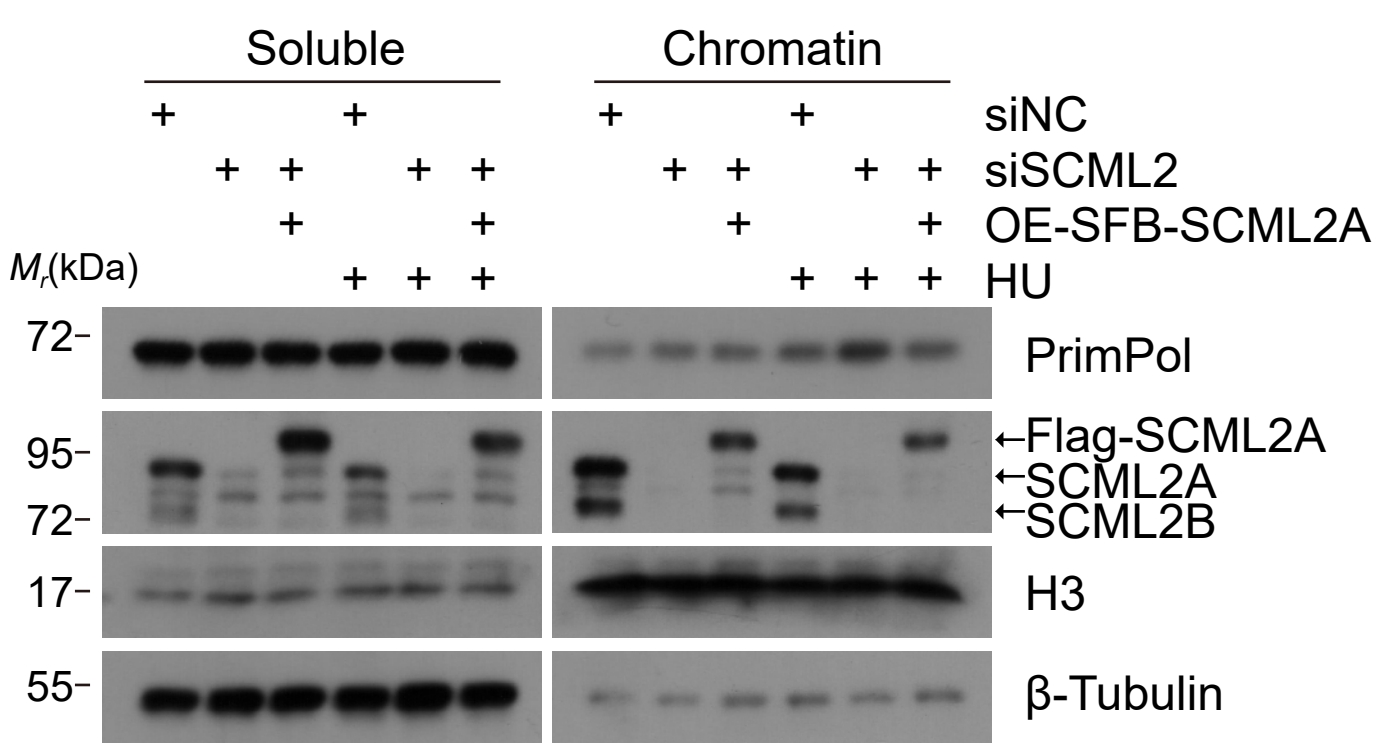
Soluble
Chromatin
siNC
siSCML2
OE-SFB-SCML2A
HU
M_r(kDa)
72
PrimPol
95
72
Flag-SCML2A
SCML2A
SCML2B
17
H3
55
β-Tubulin

Supplemental Figure 4. SAM domain of SCML2A is essential for its inhibition on PrimPol

(A) The Coomassie blue staining of the purified proteins used in the study. n=3.

(B) Biacore analysis of SCML2A or SCML2B binding to PrimPol truncations, representative of one out of three independent experiments shown in Figure 4B.

(C) GST-pull down assay using purified GST-PrimPol and His-SCML2B (wild-type or S267E mutant). n=3.

(D) Biacore analysis of $SCML2A^{WT}$, $SCML2A^{S267E}$, $SCML2B^{WT}$, or $SCML2B^{S267E}$ binding to PrimPol truncations, representative of one out of three independent experiments shown in Figure 4D.

(E) AlphaFold3 prediction of the protein charge of SCML2A and PrimPol.

(F) Co-IP of GFP-SCML2A-SAM with PrimPol truncations in HEK293T cells. n=4.

(G) AlphaFold3 prediction of the interaction between SCML2A and PrimPol (1-354).

(H) Biacore analysis of SCML2A binding to PrimPol (41-354), representative of one out of three independent experiments shown in Figure 4E.

(I) EMSA results showed that $SCML2A^{WT}$ and $SCML2A^{S267E}$, but not $SCML2A^{\Delta RBR}$, bind to the ssDNA. n=5.

(J) Immunoblot of SCML2 deficient or SCML2A-overexpressing cells after chromatin fractionation. Cells were treated with or without 50 μM HU for 1 h where indicated. n=3.

(K) Levels of SCML2 and PrimPol protein in cells as shown in Figure 4I were analyzed by western blot. n=3.

Supplemental Figure 5

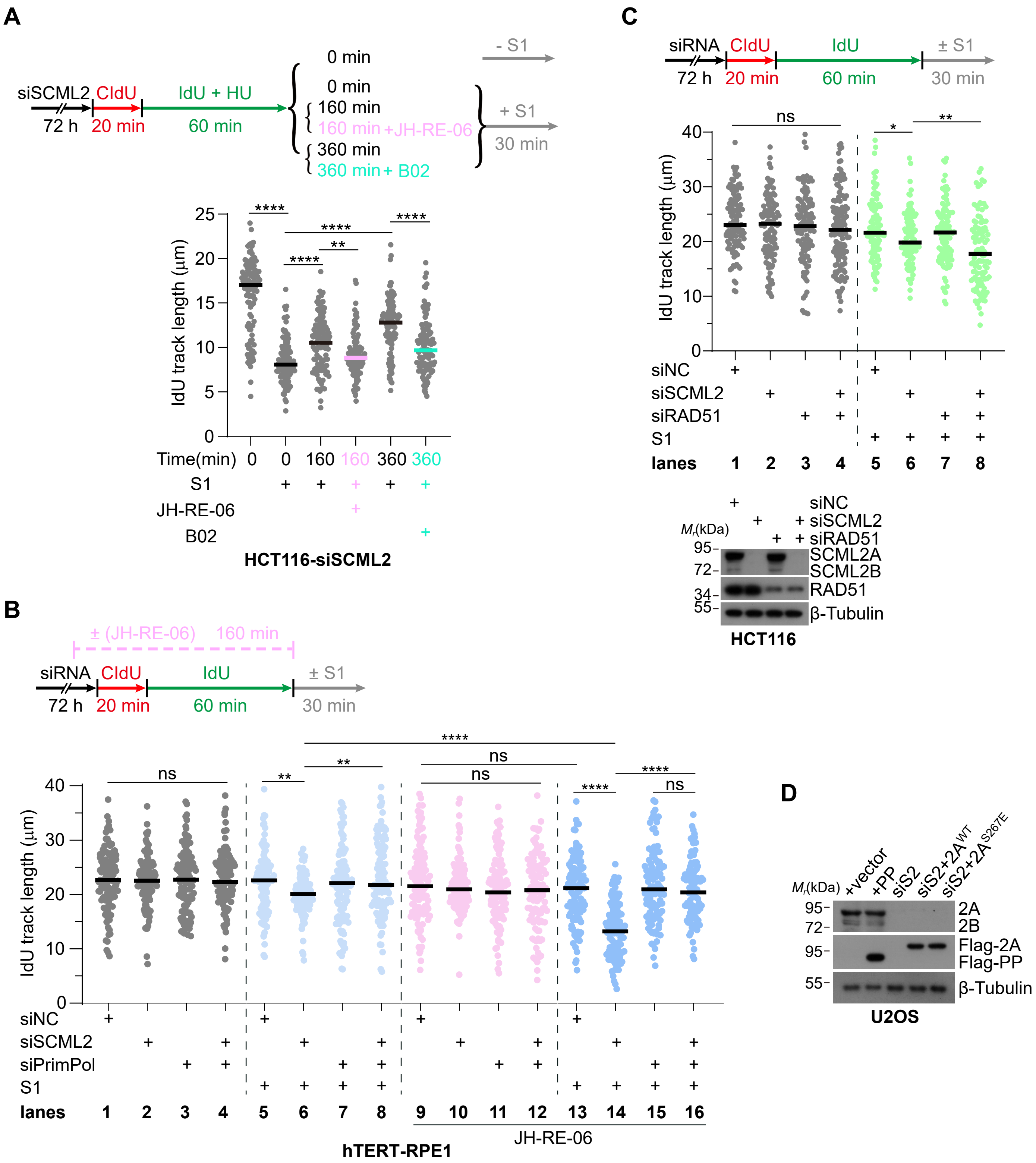

Supplemental Figure 5. SCML2 depletion promotes PrimPol engagement in cells without encountering any extra exogenous stress

(A) Experimental scheme for examining the roles of JH-RE-06 and B02 in gap repair. Following CldU/IdU labeling in the presence of 50 μM HU, SCML2-depleted cells were either subjected to S1 treatment immediately, or allowed to recover in fresh medium supplemented with 2.5 μM JH-RE-06 for 160 min or 25 μM B02 for 360 min prior to S1 treatment (top). Dot plot and median of ldU tract lengths (μm) in HCT116 SCML2-depleted cells subjected to the indicated experimental conditions (bottom). n=3.

(B) Schematic of DNA fiber tract analysis with 2.5 μM JH-RE-06 treatment followed by S1 nuclease digestion (top). Dot plot and median of ldU tract lengths (μm) in hTERT-RPE1 cells subjected to the indicated experimental conditions (bottom). n=3.

(C) Schematic of DNA fiber tract analysis (top). Dot plot and median of ldU tract lengths (μm) in HCT116 cells subjected to the indicated experimental conditions (middle). Levels of SCML2 and RAD51 were analyzed by western blot (bottom). n=3.

(D) Levels of SCML2 and PrimPol protein in cells as shown in Figure 5D were analyzed by western blot. n=3.

Supplemental Figure 6

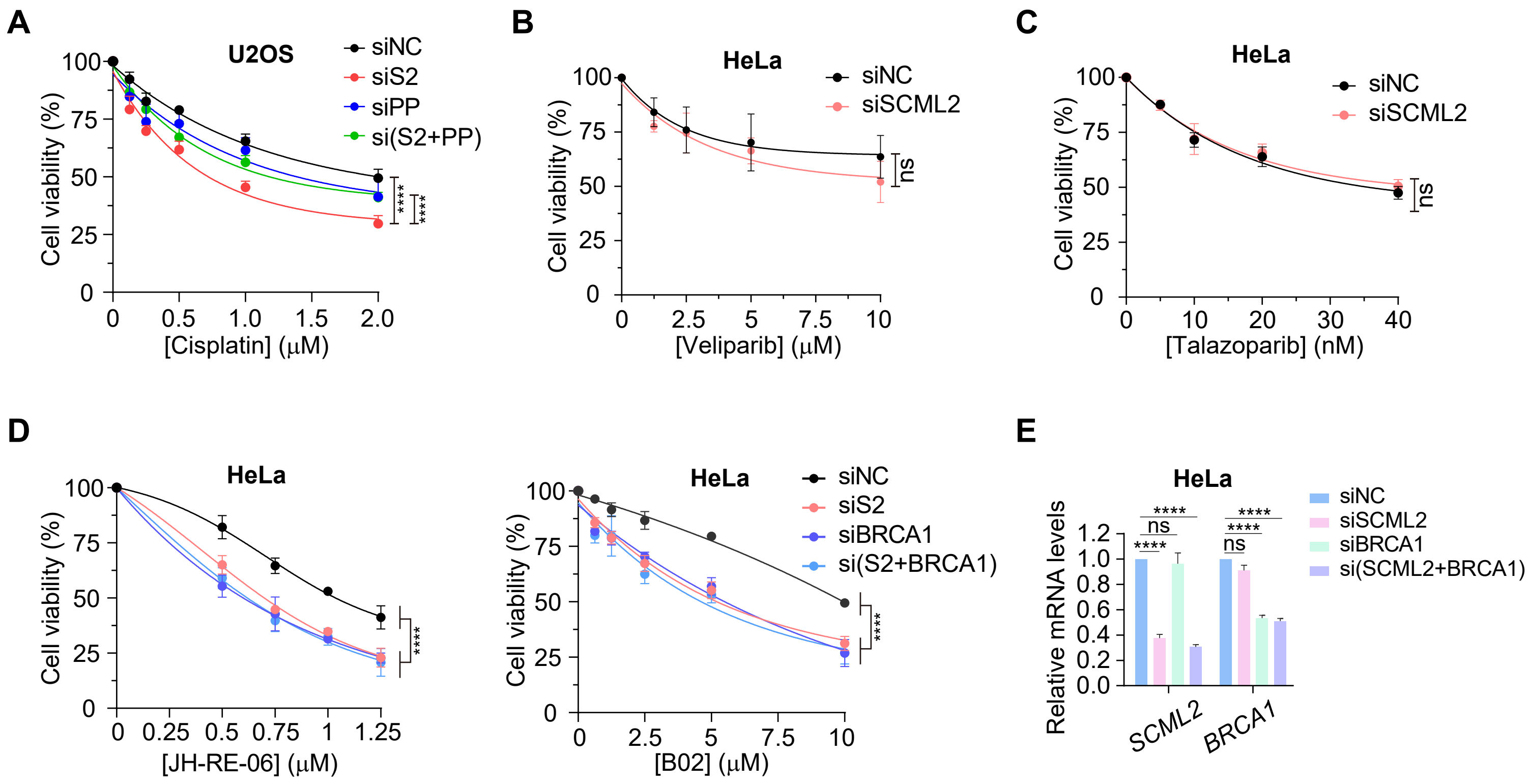

Supplemental Figure 6. SCML2 depletion potentiates JH-RE-06 or B02 cytotoxicity through PrimPol-mediated ssDNA gaps accumulation

(A) Viability of the U2OS cells transfected with the indicated siRNA after treatment with the cisplatin. n=3.

(B and C) Viability of the HeLa cells transfected with siRNA targeting SCML2 after treatment with veliparib (B) and talazoparib (C). n=3.

(D) Viability of the HeLa cells transfected with the indicated siRNA targeting SCML2 and BRCA1 after treatment with the JH-RE-06 or B02. The data in panels siNC and siS2 was the same as those in panels siNC and siS2 of Figure 6A. n=3.

(E) The mRNA levels of *SCML2* and *BRCA1* in HeLa cells after treatment with siRNA targeting SCML2 and BRCA1, as shown in supplemental figure 6D. n=3.

Data was shown as mean ± SD. The significance level was calculated by unpaired t test. **** $p<0.0001$, ns, non-significant.

Supplemental Figure 7

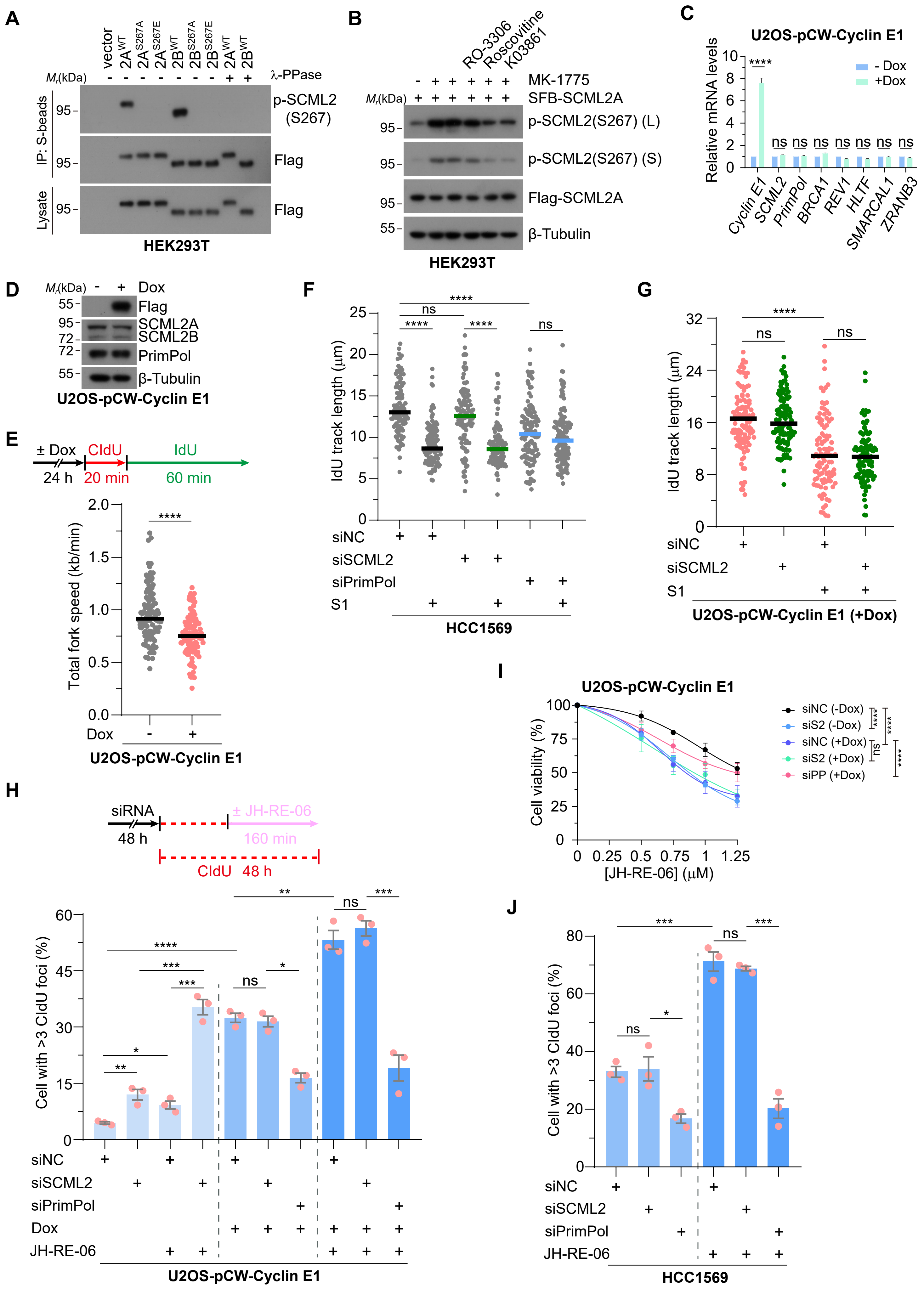

Supplemental Figure 7. CDK2-mediated phosphorylation of SCML2A Ser267 disrupts its inhibitory effect on PrimPol

(A) SCML2-Ser267 phosphorylation was examined in HEK293T cells expressing $SCML2^{WT}$, $SCML2^{S267A}$ or $SCML2^{S267E}$, analyzed by Western blotting. n=4.

(B) Following transient transfection of HEK293T cells with plasmid SFB-SCML2A for 24 hand subsequent treatment with 100 nM or 1 μM MK-1775 for 1 h, either alone or in combination with 1 μM RO-3306, 20 μM Roscovitine or 10 μM K03861 for 3 h, the phosphorylation level at site Ser267 of SCML2A was analyzed by Western blotting. n=3.

(C) The mRNA levels of the indicated genes in U2OS-Cyclin E1 Tet-On cell lines. n=3.

(D) Levels of Cyclin E1, SCML2 and PrimPol protein in U2OS-Cyclin E1 Tet-On cell lines were analyzed by western blot. n=3.

(E) Dot plot and median of total fork speed in U2OS-Cyclin E1 Tet-On cell lines. n=3.

(F and G) Dot plot and median of ldU tract lengths (μm) in HCC1569 cells (E) and U2OS-Cyclin E1 Tet-On cell lines (F) transfected with the indicated siRNA after treatment with or without S1. n=3.

(H) Schematic of non-denaturing CldU staining analysis with 2.5 μM JH-RE-06 (top). Percentage of U2OS-Cyclin E1 Tet-On cell lines containing >3 CldU foci per nucleus after treatment with the indicated siRNAs (bottom). n=3.

(I) Viability of the U2OS-Cyclin E1 Tet-On cell lines transfected with the indicated siRNA after treatment with the JH-RE-06. n=3.

(J) Percentage of HCC1569 cells containing >3 CldU foci per nucleus transfected with the indicated siRNA after treatment with or without 2.5 μM JH-RE-06. n=3.

*p<0.05, **p<0.01, ***p<0.001, ****p < 0.0001, ns, non-significant.

Table S1

Sequence of CRISPR-Cas9 guide-RNAs (gRNAs) and siRNA used in this study

Name	Sequence
SCML2#1(gRNAs)	TGCCGGCTCGCGCGATCGGT
SCML2#2(gRNAs)	GTGCCGGCTCGCGCGATCGG
SCML2#3(gRNAs)	GTCCCACCGATCGCGCGAGC
p53#1(gRNAs)	CAGAATGCAAGAAGCCCAGA
p53#2(gRNAs)	CCCCGGACGATATTGAACAA
p21#1(gRNAs)	GTCACCGAGACACCACTGGA
siNC	5'-UUCUCCGAACGUGUCACGUTT-3'
siSCML2#1	5'-CCAAACGAUCUCCUCAGCAAATT-3'
siSCML2#2	5'-CAGUAUGUAUUGCUACGGUUATT-3
siPrimPol#1	5'-GAGGAAACCGUUGUCCUCAGUGUAUTT-3'
siPrimPol#2	5'-AGAAAAGGCUACAGAGGAA-3'
siHLTF	5'-GGAAACAAUACCAGUGAAA-3'
siSMARCAL1	5'-GAAUCUCACUUCCUCAAAA-3'
siBRCA1	5'-GGAACCUGUCUCCACAAAG-3'
siRAD51	5'-UCAGAGAUCAUACAGAUUA-3'
sip21#1	5'-GGAACAAGGAGUCAGACAU-3'
sip21#2	(Ambion, s416)

Table S2

Primers for RT-qPCR

Name	Sequence (5' → 3')
SCML2-F	TCAGTCTCCCAGGATTTTTCTC
SCML2-R	GCTTCACTTTTCCTCTGCATTT
PrimPol-F	CCATTACCTGCTGAAGTATGTCTC
PrimPol -R	GCATCAGCAGATGCACCTGTT
HLTF-F	GTTCAAAGATTAATGCGCT
HLTF-R	AAAGACAGGAATGTTGTAAACTGAGA
ZRANB3-F	CAGCCAGGTAATGAACAGTGG
ZRANB3-R	AGCTGAAAGCTTGCTGGTAA
SMARCAL1-F	ACAGCATCAGAGGACTAGCTC
SMARCAL1-R	CACTGGCTTACAAGACTCCCT
BRCA1-F	ACCTTGGAACTGTGAGAACTCT
BRCA1-R	TCTTGATCTCCCACACTGCAATA
Cyclin E1-F	AAGGAGCGGGACACCATGA
Cyclin E1-R	ACGGTCACGTTTGCCTTCC
Rev1-F	GTCCAGCAAGCAGAGTCACA
Rev1-R	GGAAGGGCAGCAAATACCTC
Actin-F	CTCGCCTTTGCCGATCC
Actin-R	ATGCCGGAGCCGTTGTC

Table S3

KEY RESOURCES TABLE

REAGENT or RESOURCE	SOURCE	IDENTIFIER
**Antibodies**		
Rabbit polyclonal anti-SCML2	Proteintech	Cat#25544-1-AP; RRID: AB_2880127
Rabbit polyclonal anti-PrimPol	Proteintech	Cat#29824-1-AP; RRID: AB_2918349
Rabbit polyclonal anti-Flag	Sigma	Cat#F7425; RRID: AB_439687
Rabbit polyclonal anti-GFP	Sigma	Cat#SAB4301138; RRID: AB_ 2750576
Rabbit monoclonal anti-p21	ABclonal	Cat#A19094; RRID: AB_2862586
Rabbit polyclonal anti-p53	ABclonal	Cat#A11232; RRID: AB_2861528
Rabbit polyclonal anti-β-tubulin	Abcam	Cat#ab6046; RRID: AB_2210370
Rabbit polyclonal anti- Phospho -SCML2(Ser267)	This paper	N/A
Rat monoclonal [BU1/75 (ICR1)] to BrdU	Abcam	Cat#ab6326; RRID: AB_305426
Mouse Anti-BrdU clone B44	BD	Cat#347580; RRID: AB_10015219
Rabbit polyclonal anti-CHK1	ABclonal	Cat#A7653; RRID: AB_2768892
Rabbit monoclonal anti-Phospho-CHK1 (Ser345) (133D3)	Cell Signaling Technology	Cat#2348; RRID: AB_331212
Rabbit monoclonal [51-8] anti- Thiophosphate ester	Abcam	Cat#ab92570; RRID: AB_10562142
Rabbit polyclonal anti-RAD51	Proteintech	Cat#14961-1-AP; RRID: AB_2177083
Goat anti-Rat IgG (H+L) Cross-Adsorbed Secondary Antibody, DyLight™ 550	Invitrogen	Cat#SA5-10019; RRID: AB_2556599
Alexa Fluor® 488 AffiniPure Goat Anti-Mouse IgG (H+L)	Jackson	Cat#115-545-003; RRID: AB_2338840
**Bacterial and virus strains**		
E. Coli BL21	NEB	Cat#C2530H
**Chemicals, peptides, and recombinant proteins**		
Hydroxyurea (HU)	Sigma	Cat#H8627
Protease Inhibitor Cocktail	MCE	Cat#HY-K0010
JH-RE-06	MCE	Cat#HY-126214
Isopropyl-β-D-thiogalactopyranoside (IPTG)	Sigma	Cat#I6758
Doxycycline hyclate (dox)	Sigma	Cat#D9891
CD437	Sigma	Cat#C5865
CldU (5-chloro-2'-deoxyuridine)	Sigma	Cat#C6891
IdU (5-Iodo-2'-deoxyuridine)	Sigma	Cat#I7125
S1 nuclease	Thermo Fisher	Cat#EN0321
B02	Selleck	Cat#S8434
Talazoparib	Selleck	Cat#S7048
Veliparib	Selleck	Cat#S1004
cisplatin	Sigma	Cat#C2210000
K03861	Selleck	Cat#S8100

RO-3306	Selleck	Cat#S7747
Roscovitine	Selleck	Cat#S1153
MK-1775	Selleck	Cat#S1525
Cdk2/Cyclin E	Sigma	Cat#14-475
**Critical commercial assays**		
Lipofectamine RNAiMAX Reagent	Invitrogen	Cat#13778150
Lipofectamine 2000	Thermo Fisher	Cat#11668027
Lipofectamine 3000	Thermo Fisher	Cat#L3000008
BCA protein assay kit	Thermo Fisher	Cat#23225
CCK8 Counting Kit	Beyotime	Cat#C0038
Hiscript cDNA synthesis kit	Vazyme	Cat#R223-01
SYBR qPCR mix	Vazyme	Cat#Q311-02
**Experimental models: cell lines**		
HEK293T	ATCC	Cat#CRL-3216; RRID: CVCL_0063
U2OS	ATCC	Cat#HTB-96; RRID: CVCL_0042
U2OS-pCW-Cyclin E1	This paper	N/A
HCT116	ATCC	Cat# CCL-247; RRID: CVCL_0291
HCT116-SCML2$^{-/-}$	This paper	N/A
HCT116-SCML2$^{-/-+\text{FLAG-SCML2A}}$	This paper	N/A
HCT116-SCML2$^{-/-+\text{FLAG-SCML2A (S441A)}}$	This paper	N/A
HCT116-SCML2$^{-/-+\text{FLAG-SCML2A (S267A)}}$	This paper	N/A
HCT116-SCML2$^{-/-+\text{FLAG-SCML2A (S267E)}}$	This paper	N/A
HCT116-SCML2$^{-/-+\text{FLAG-SCML2B}}$	This paper	N/A
HCT116-p53$^{-/-}$	This paper	N/A
HCT116-p21$^{-/-}$	This paper	N/A
NCI-H1299	ATCC	Cat#CRL-5803; RRID: CVCL_0060
Huh7	JCRB	Cat#JCRB0403
HeLa	ATCC	Cat#CRM-CCL-2; RRID: CVCL_0030
HCC1569	ATCC	Cat#CRL-2330; RRID: CVCL_1255
hTERT-RPE1	ATCC	Cat#CRL-4000; RRID: CVCL_4388
**Recombinant DNA**		
pMD2.G	Addgene	Cat#1864; RRID: Addgene_1864
psPAX2	Addgene	Cat#12259; RRID: Addgene_12259
pCW-cas9-puro	Addgene	Cat#50661; RRID: Addgene_50661
pCW-cas9-blast	Addgene	Cat#83481; RRID: Addgene_83481
pCW-cas9-puro-SCML2A	This paper	N/A
pCW-cas9-puro-SCML2A(S441A)	This paper	N/A
pCW-cas9-puro-SCML2B	This paper	N/A
pCW-cas9- puro-cyclin E1	This paper	N/A
pCW-cas9- blast-SCML2A	This paper	N/A

pCW-cas9- blast-SCML2A(S441A)	This paper	N/A
pCW-cas9- blast-SCML2A(S267A)	This paper	N/A
pCW-cas9- blast-SCML2A(S267E)	This paper	N/A
pCW-cas9- blast-SCML2B	This paper	N/A
SFB-vector	This paper	N/A
SFB-SCML2A	This paper	N/A
SFB-SCML2B	This paper	N/A
SFB-SCML2A-ΔMBT	This paper	N/A
SFB-SCML2A-ΔRBR	This paper	N/A
SFB-SCML2A-ΔSLED	This paper	N/A
SFB-PrimPol	This paper	N/A
SFB-PrimPol (1-354aa)	This paper	N/A
pEGFP-C1	This paper	N/A
GFP- PrimPol	This paper	N/A
GFP- PrimPol (1-354aa)	This paper	N/A
GFP- PrimPol (1-487aa)	This paper	N/A
GFP- PrimPol-ΔAEP	This paper	N/A
GFP- SCML2A	This paper	N/A
GFP- SCML2A-SAM	This paper	N/A
pGEX-4T-1	Addgene	Cat#52301; RRID: Addgene_52301
GST-PrimPol	This paper	N/A
GST-PrimPol (1-354aa)	This paper	N/A
GST-PrimPol (ΔAEP)	This paper	N/A
GST-PrimPol-RBD	This paper	N/A
GST-PrimPol (1-487aa)	This paper	N/A
GST-PrimPol-ZF	This paper	N/A
GST-PrimPol (41-354aa)	This paper	N/A
pET-28a	Addgene	Cat#42631; RRID: Addgene_42631
His-SCML2A	This paper	N/A
His-SCML2A (S267E)	This paper	N/A
His-SCML2A-ΔRBR	This paper	N/A
His-SCML2B	This paper	N/A
His-SCML2B (S267E)	This paper	N/A
His-SCML2B-ΔRBR	This paper	N/A
**Software and algorithms**		
GraphPad Prism	GraphPad Software	https://www.graphpad.com
ImageJ	ImageJ	https://imagej.net/Downloads

Figure 1

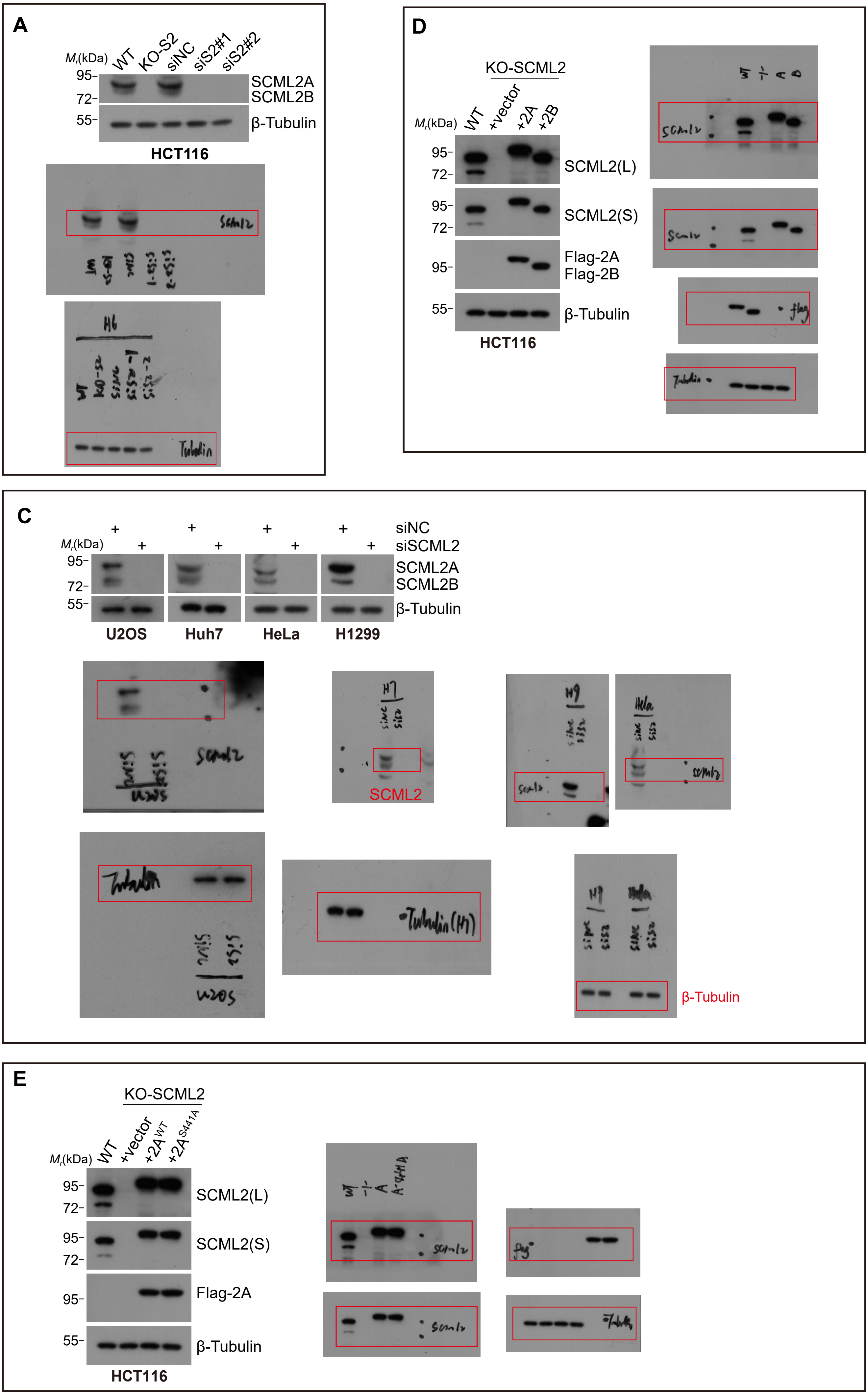
A
Mr(kDa)
WT
KO-S2
siNC
siS2#1
siS2#2
95-
72-
55-
SCML2A
SCML2B
β-Tubulin
HCT116
D
KO-SCML2
Mr(kDa)
WT
+vector
+2A
+2B
95-
72-
55-
SCML2(L)
SCML2(S)
Flag-2A
Flag-2B
β-Tubulin
HCT116
C
Mr(kDa)
siNC
siSCML2
95-
72-
55-
SCML2A
SCML2B
β-Tubulin
U2OS
Huh7
HeLa
H1299
SCML2
β-Tubulin
E
KO-SCML2
Mr(kDa)
WT
+vector
+2A WT
+2A S441A
95-
72-
55-
SCML2(L)
SCML2(S)
Flag-2A
β-Tubulin
HCT116

Figure 3

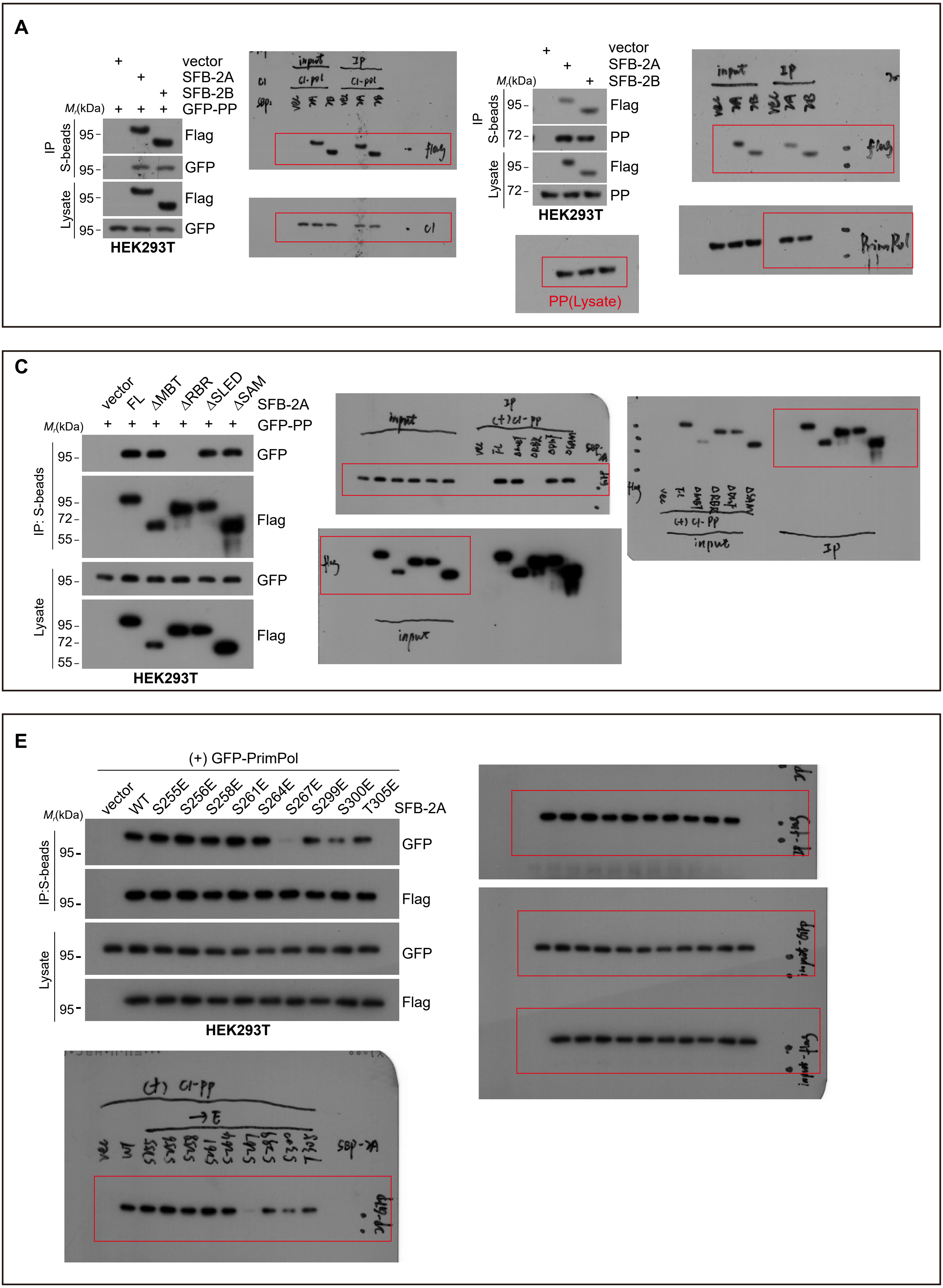
A
vector
SFB-2A
SFB-2B
GFP-PP
M_r(kDa)
IP S-beads
Lysate
95
Flag
GFP
HEK293T
72
PP
PP(Lysate)
C
vector
FL
ΔMBT
ΔRBR
ΔSLED
ΔSAM
SFB-2A
GFP-PP
IP: S-beads
Lysate
55
HEK293T
E
(+) GFP-PrimPol
WT
S255E
S256E
S258E
S261E
S264E
S267E
S299E
S300E
T305E
IP:S-beads
HEK293T

Figure 4

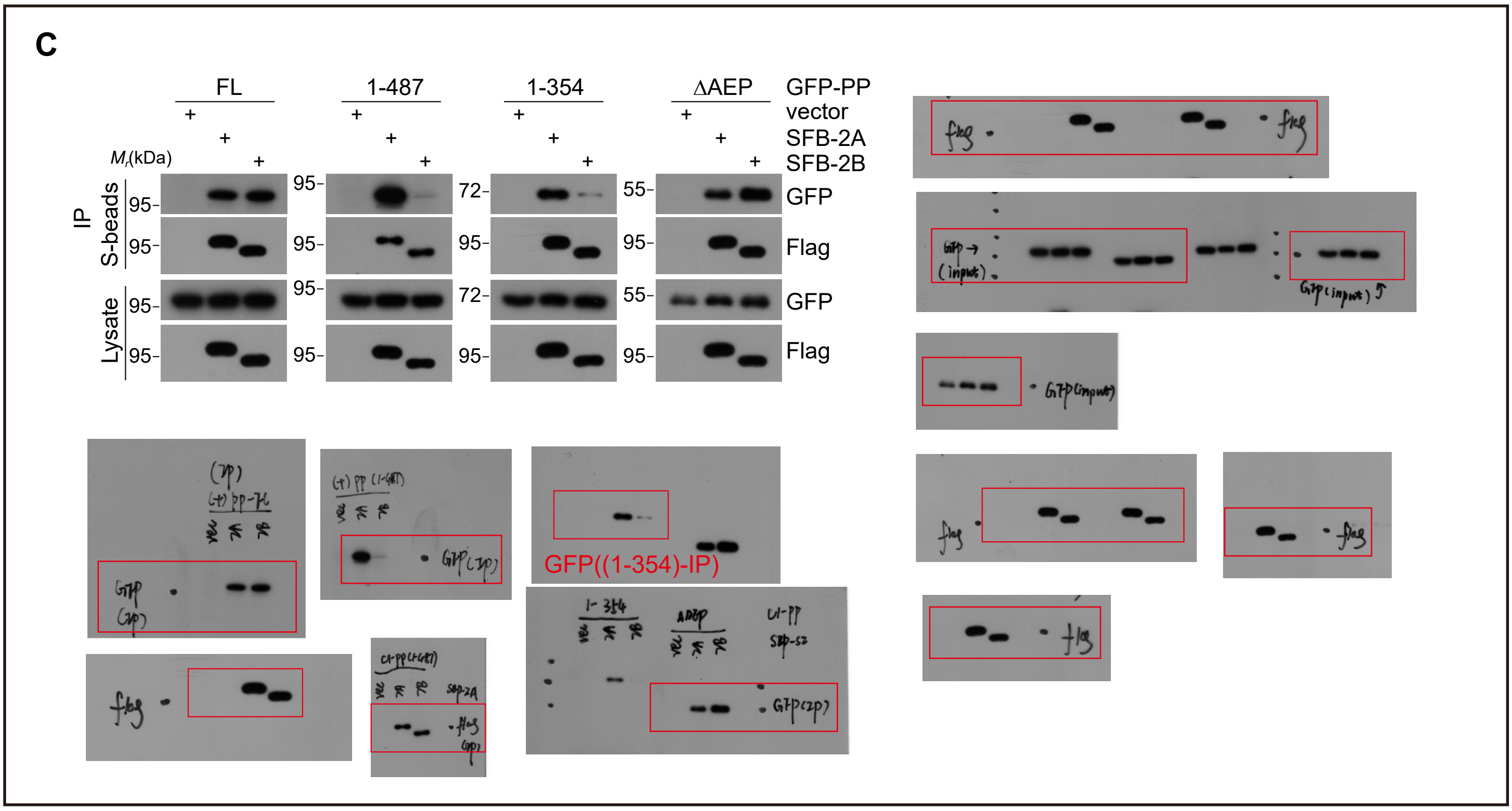

Figure 7

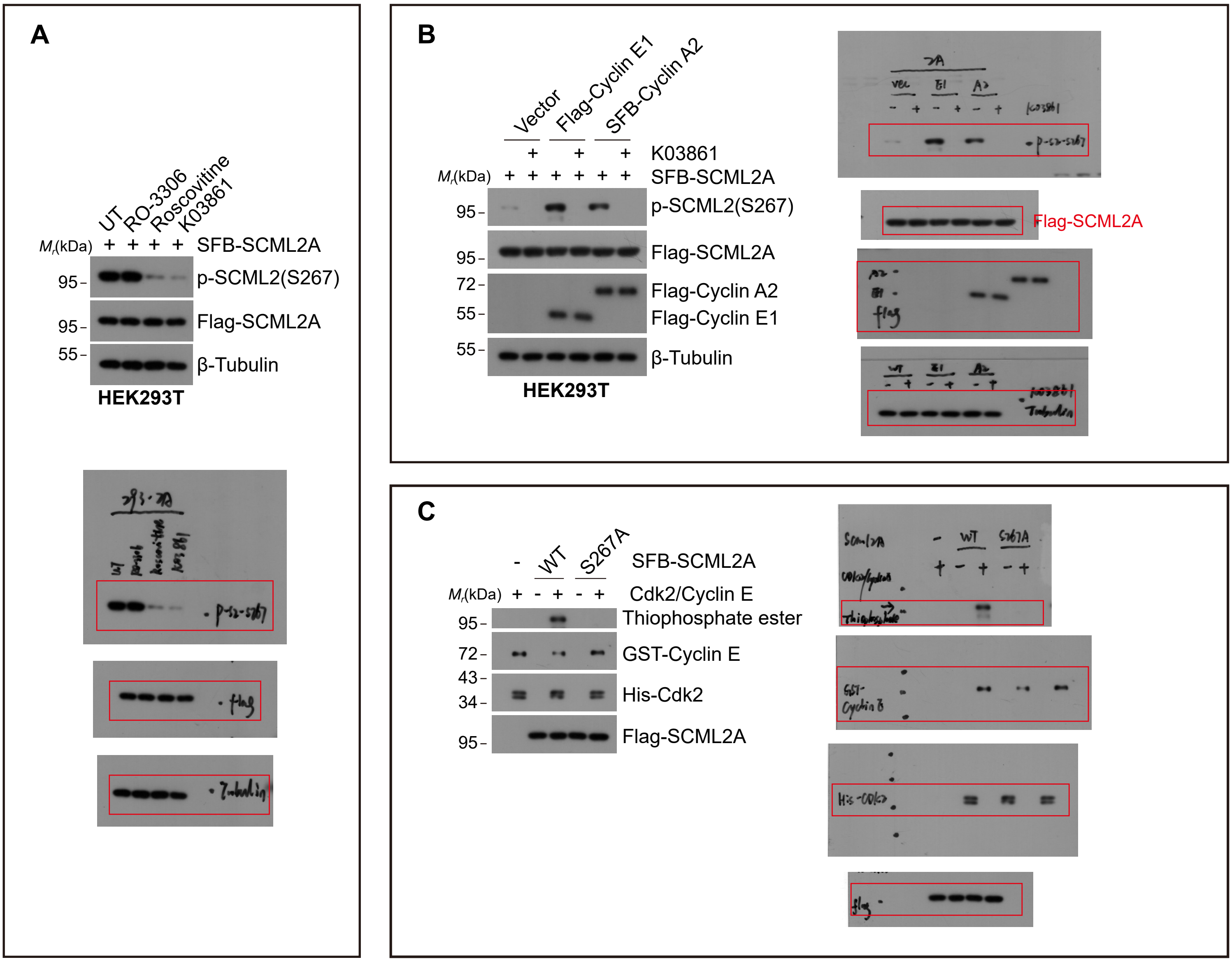

Figure 7

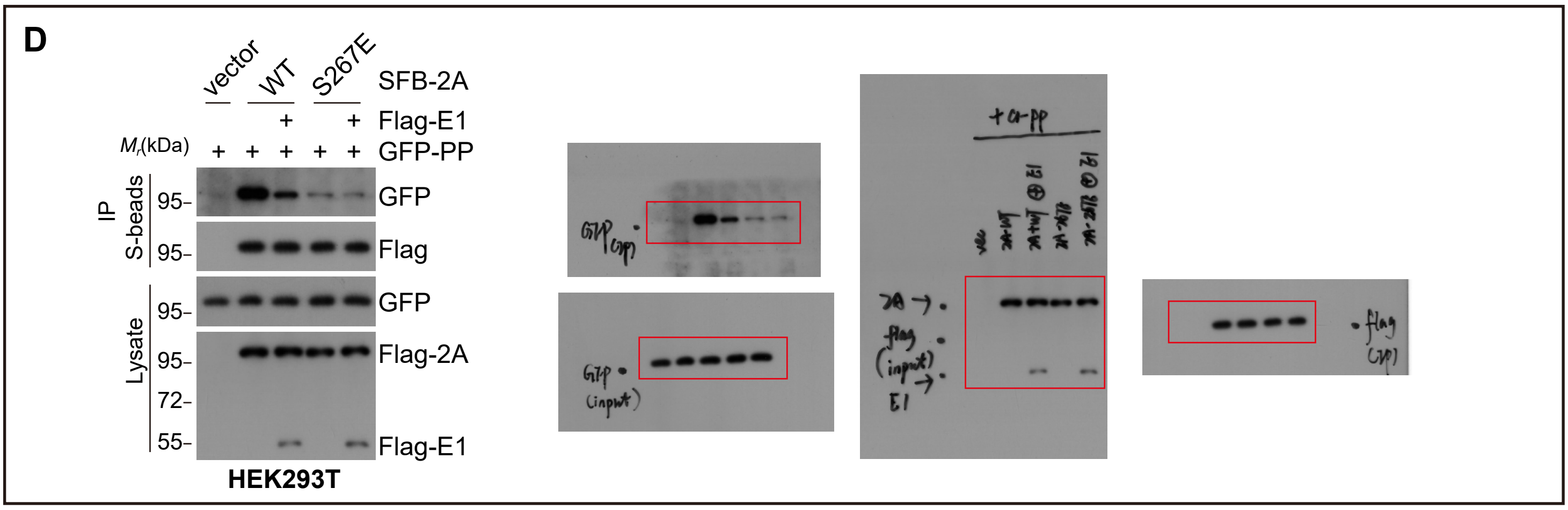

Supplemental Figure 1

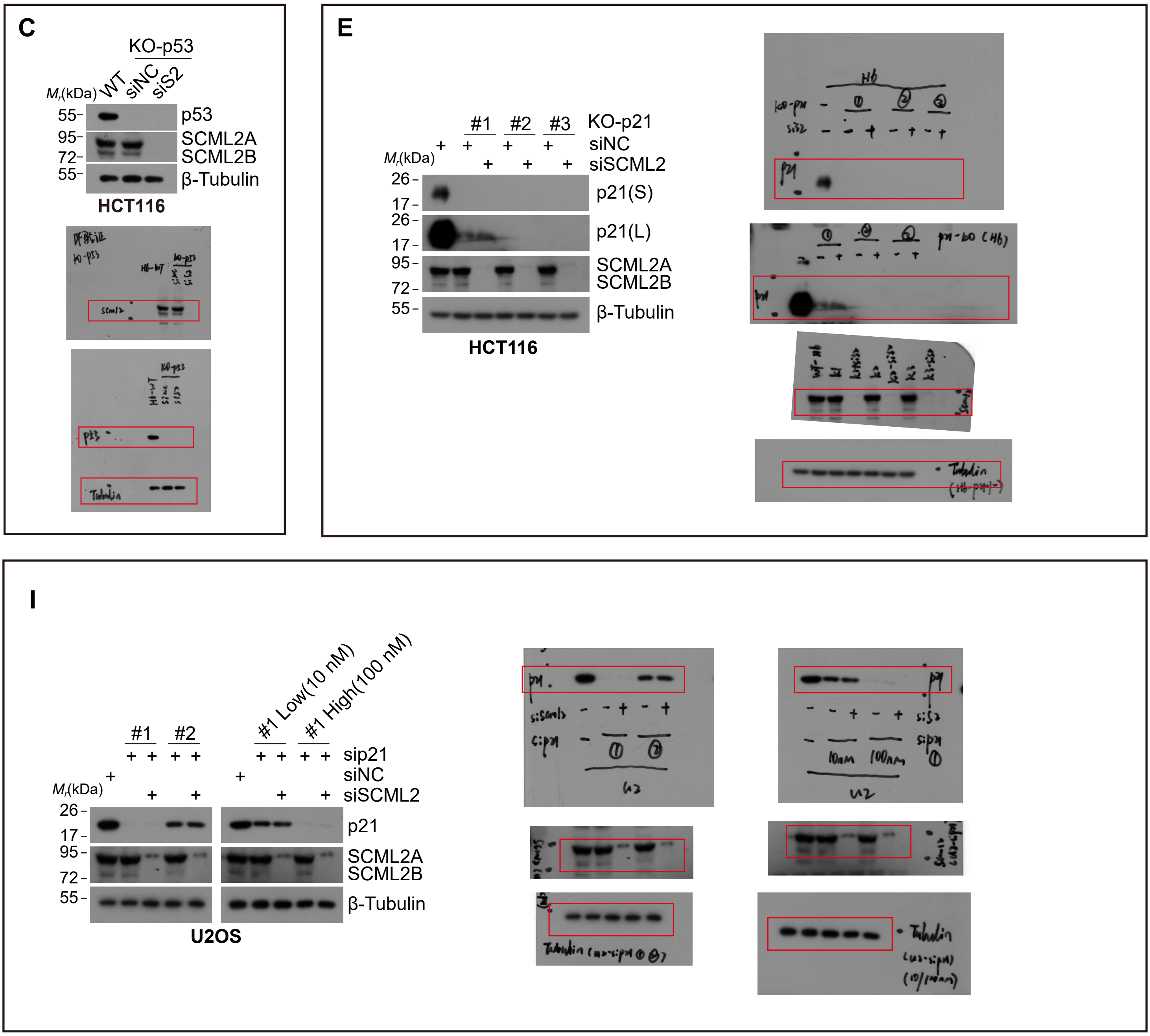

Supplemental Figure 2

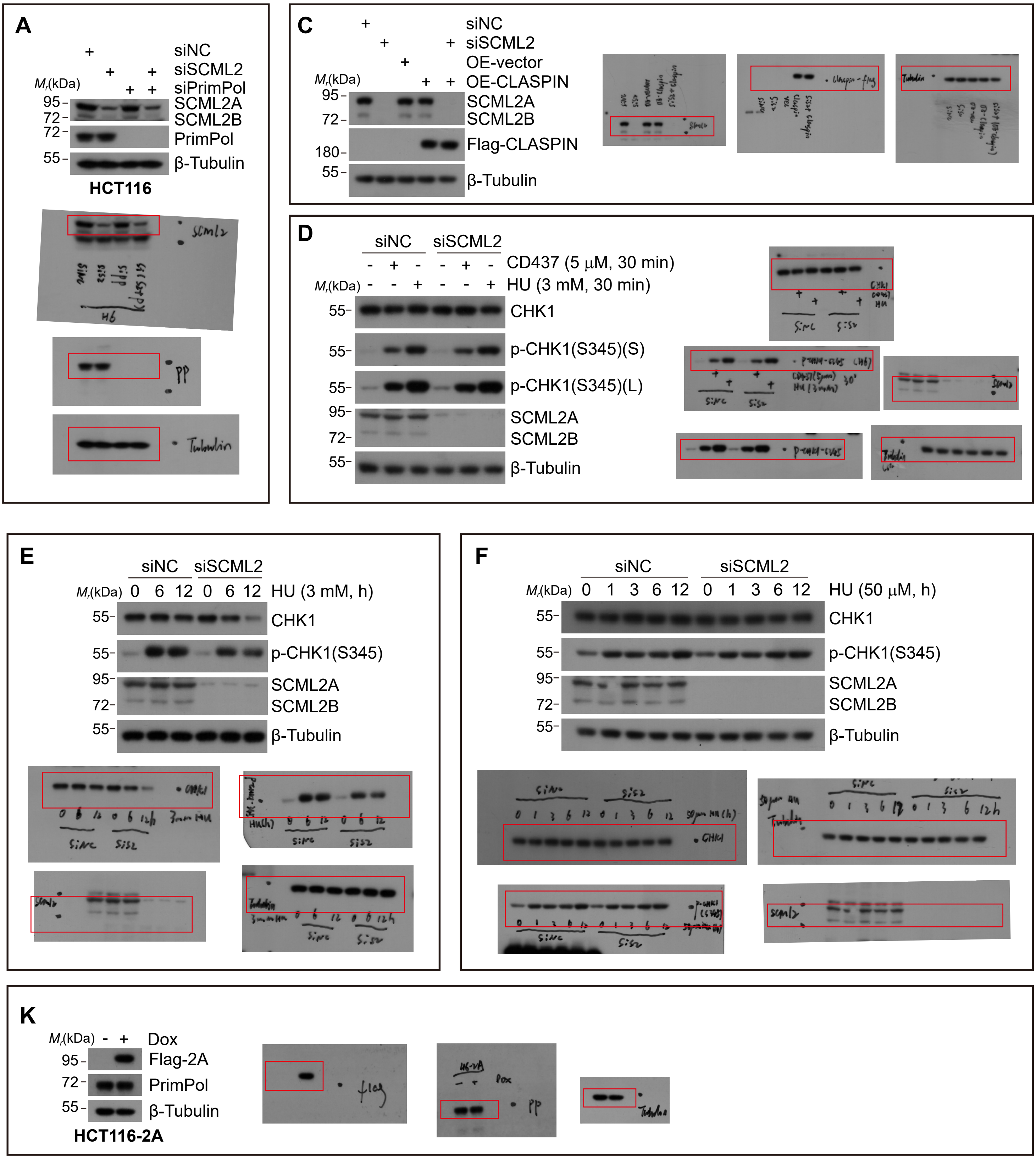

Supplemental Figure 3

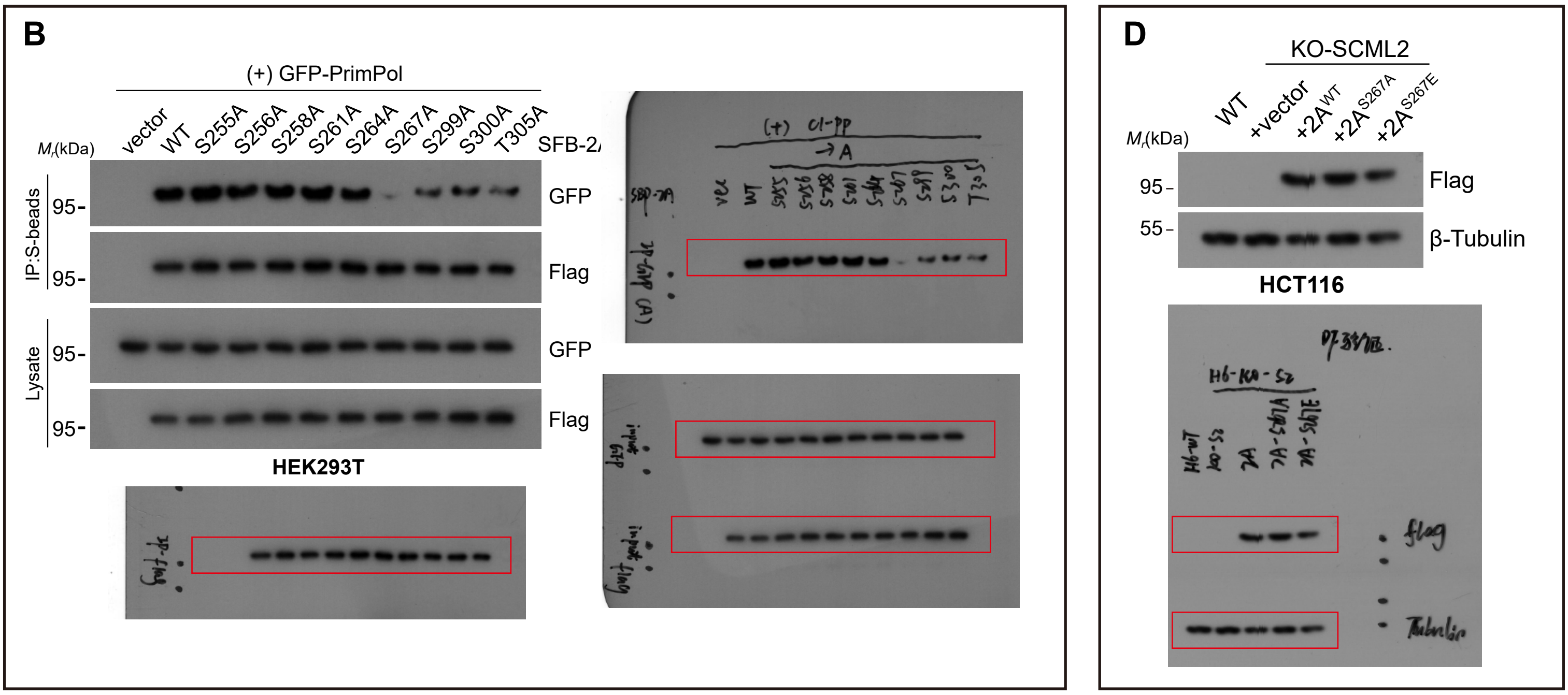

Supplemental Figure 4

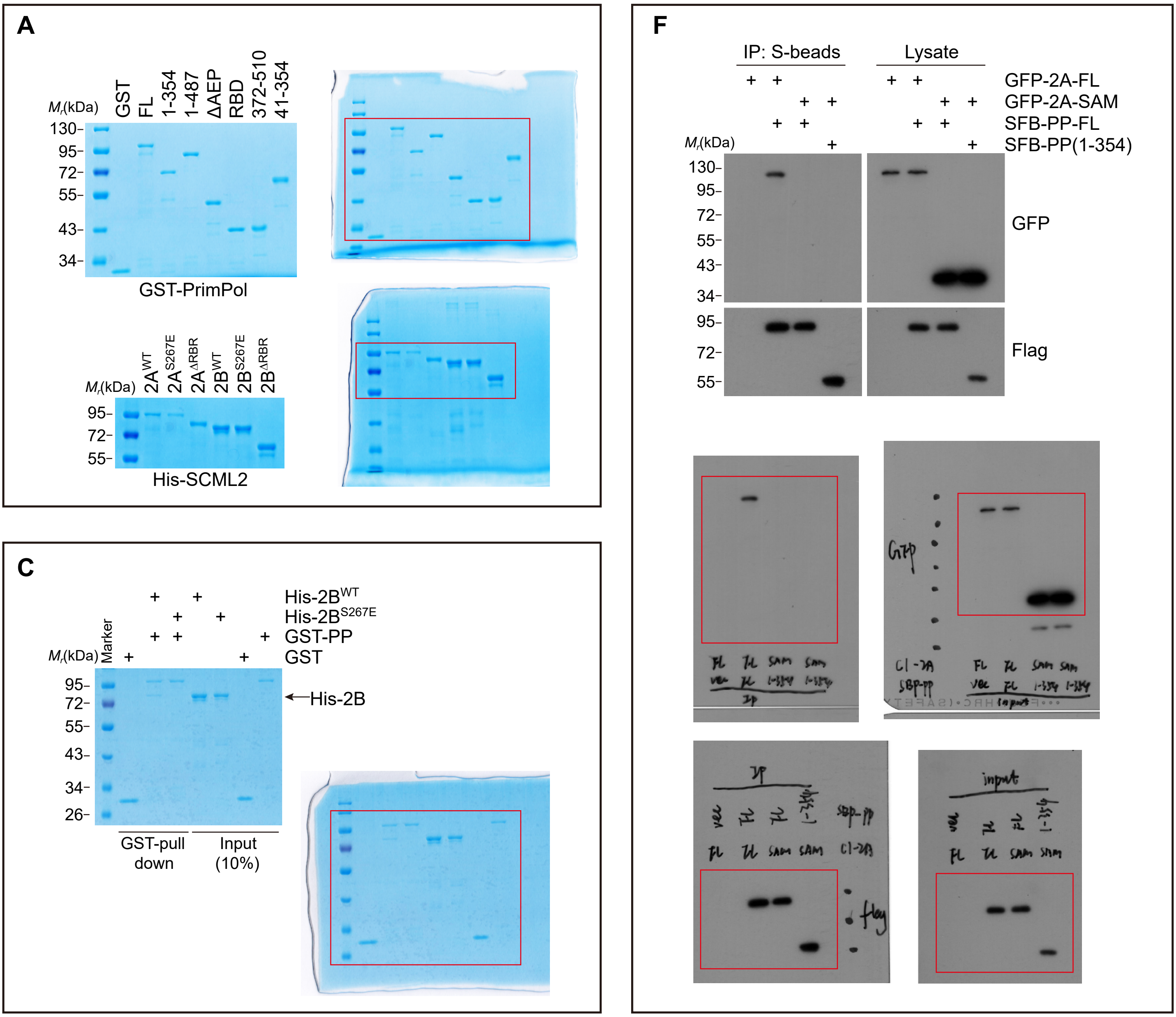

Supplemental Figure 4

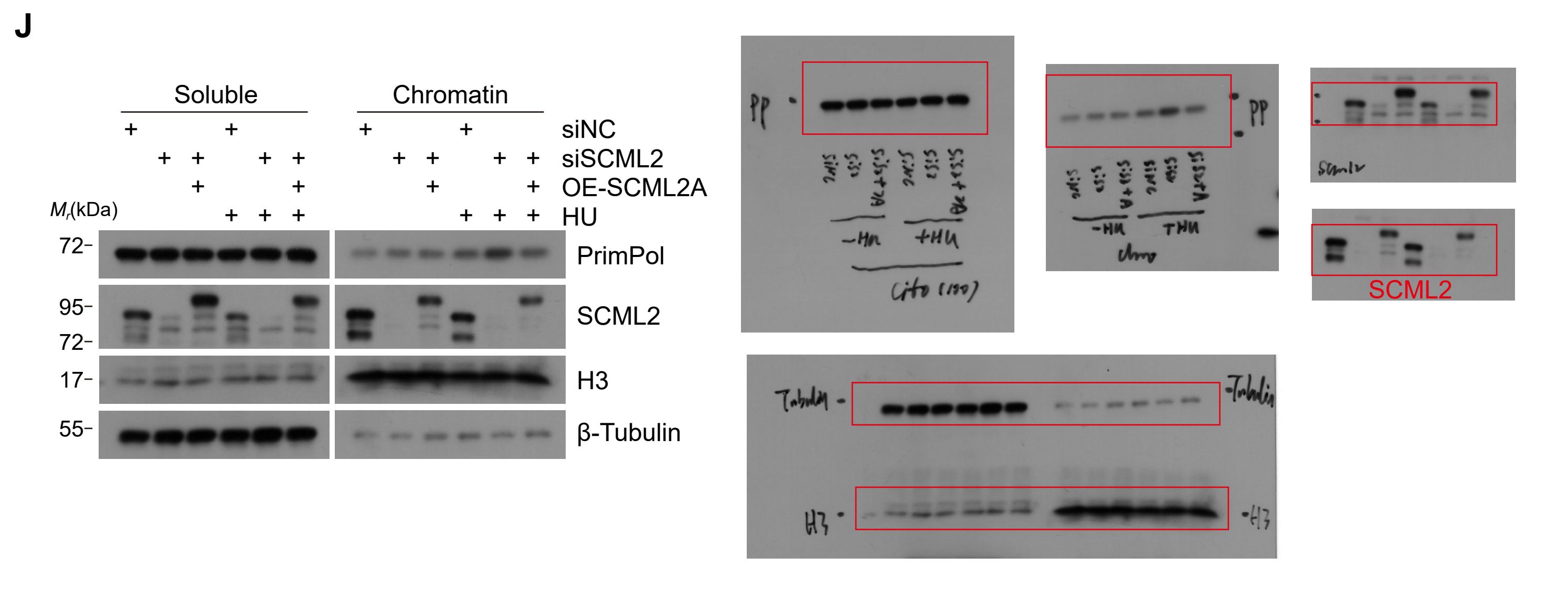

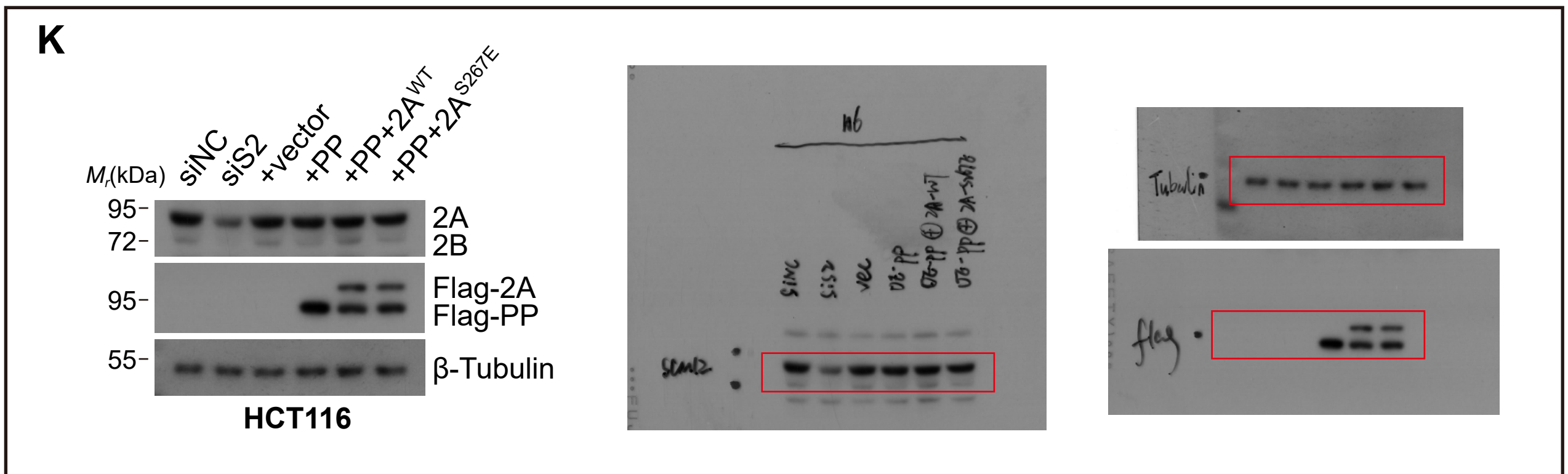

Supplemental Figure 5

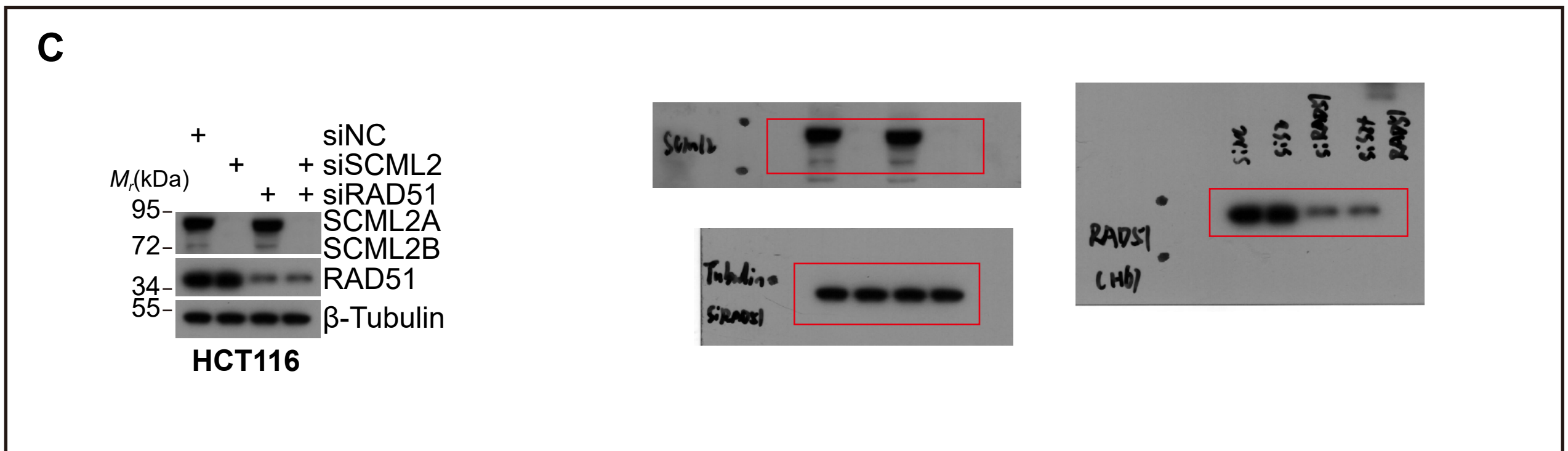

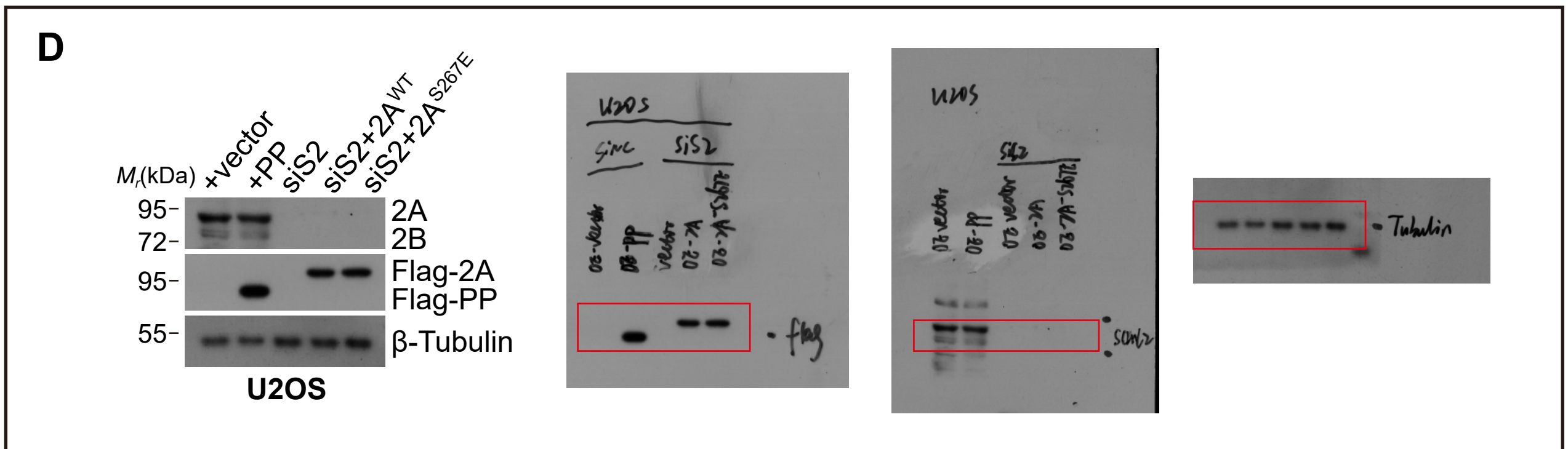

Supplemental Figure 7

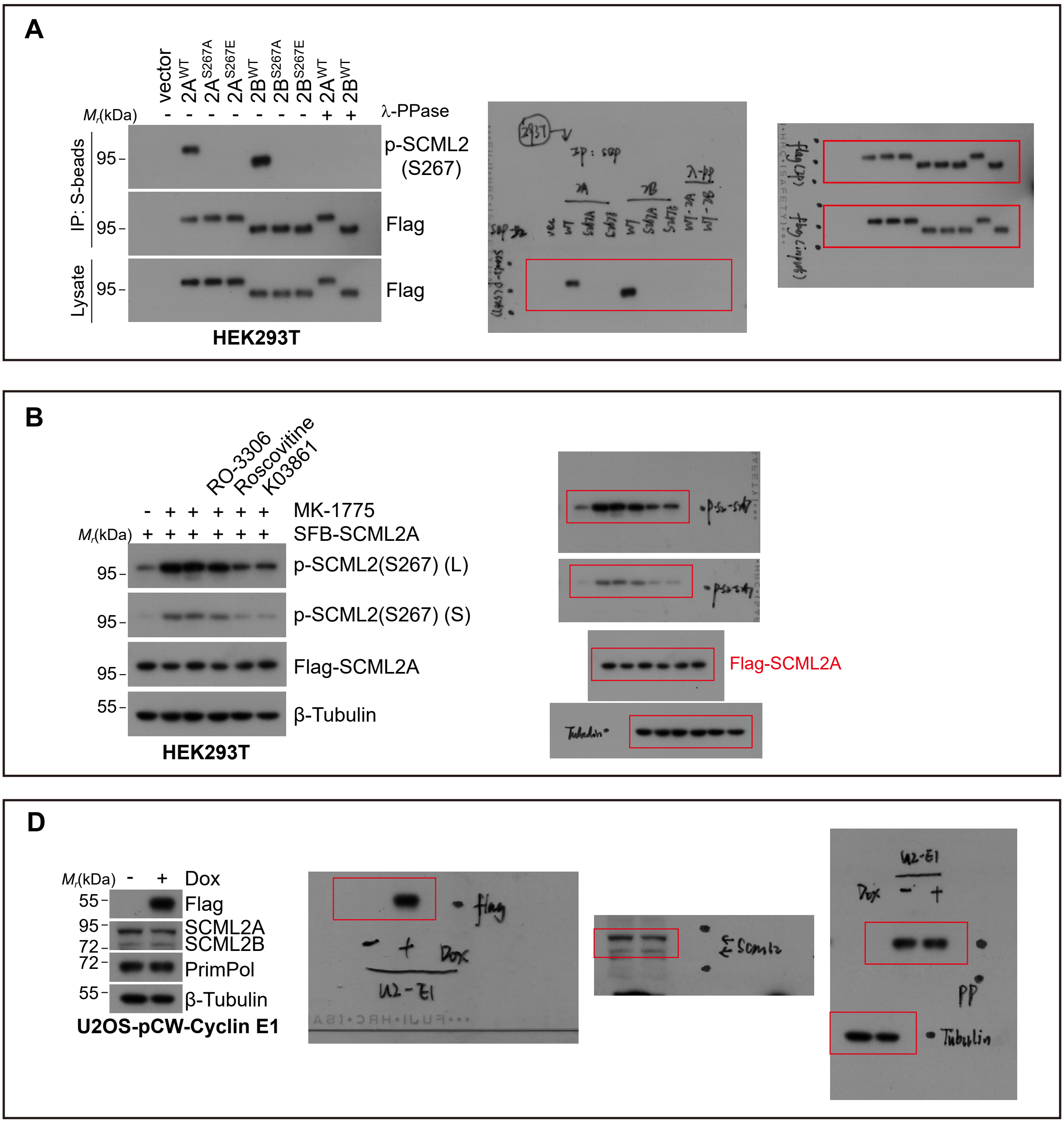

Supplement: Supplementary file 1 [file sciadv.aee4738_sm.pdf]
